# A Primer on Constructing Plasticity Phenotypes to Classify Experience-Dependent Development of the Visual Cortex

**DOI:** 10.3389/fncel.2020.00245

**Published:** 2020-08-27

**Authors:** Justin L. Balsor, Dezi Ahuja, David G. Jones, Kathryn M. Murphy

**Affiliations:** ^1^McMaster Integrative Neuroscience Discovery and Study (MiNDS) Program, McMaster University, Hamilton, ON, Canada; ^2^Department of Psychology, Neuroscience & Behavior, McMaster University, Hamilton, ON, Canada; ^3^Pairwise Affinity Inc., Dundas, ON, Canada

**Keywords:** high-dimensional analysis, development, human, cluster, fluoxetine, amblyopia, translation, synaptic plasticity

## Abstract

Many neural mechanisms regulate experience-dependent plasticity in the visual cortex (V1), and new techniques for quantifying large numbers of proteins or genes are transforming how plasticity is studied into the era of big data. With those large data sets comes the challenge of extracting biologically meaningful results about visual plasticity from data-driven analytical methods designed for high-dimensional data. In other areas of neuroscience, high-information content methodologies are revealing more subtle aspects of neural development and individual variations that give rise to a richer picture of brain disorders. We have developed an approach for studying V1 plasticity that takes advantage of the known functions of many synaptic proteins for regulating visual plasticity. We use that knowledge to rebrand protein measurements into plasticity features and combine those into a plasticity phenotype. Here, we provide a primer for analyzing experience-dependent plasticity in V1 using example R code to identify high-dimensional changes in a group of proteins. We describe using PCA to classify high-dimensional plasticity features and use them to construct a plasticity phenotype. In the examples, we show how to use this analytical framework to study and compare experience-dependent development and plasticity of V1 and apply the plasticity phenotype to translational research questions. We include an R package “PlasticityPhenotypes” that aggregates the coding packages and custom code written in RStudio to construct and analyze plasticity phenotypes.

## Introduction

Development of the primary visual cortex (V1) is regulated by many neurobiological mechanisms that form a complex set of cellular and molecular states to enhance or reduce experience-dependent plasticity. Often, studies of V1 development and plasticity focus on just a few of those mechanisms. However, the field is rapidly moving to large-scale studies that measure tens to thousands of plasticity-related markers to understand how V1 develops (Nowakowski et al., 2017; Siu et al., 2017; Lehallier et al., 2019) and changes in response to disease (Smith et al., 2016, 2019) or visual experience (Majdan and Shatz, 2006; Tropea et al., 2006; Beston et al., 2010; Dahlhaus et al., 2011; Balsor et al., 2019b). The complexity of the data is posing new challenges for understanding the molecular mechanisms (proteins or genes) that underpin experience-dependent development and plasticity of vision. Nevertheless, big-data studies of protein or gene expression hold the potential of revealing subtle aspects of V1 development and plasticity that might affect visual function. Those studies can also enhance translation from animal models to humans by measuring the same plasticity features in different species. The idea of a plasticity phenotype is one way to tackle those challenges. Here, we present a primer for discovering collections of plasticity-related proteins, rebranding those into plasticity features, combining features to construct a plasticity phenotype, and using the phenotype to classify normal and abnormal development of V1.

The term plasticity phenotype has been used to describe the waxing and waning of gene expression during the critical period (CP) (Smith et al., 2019). It has also been used as a tool to rebrand high-dimensional patterns of protein expression into a reduced set of plasticity-related features (Balsor et al., 2019b). In this paper, we take advantage of insights gained from previous studies that identified the role of various neural proteins in V1 development and plasticity, especially glutamatergic and GABAergic receptor subunits, to select the set of proteins used for the examples in this primer (Froemke et al., 2005; Hensch, 2005; Philpot et al., 2007; Maffei and Turrigiano, 2008; Yashiro and Philpot, 2008; Cho et al., 2009; Smith et al., 2009; Heimel et al., 2011; Turrigiano, 2011; Cooke and Bear, 2012; Durand et al., 2012; Hensch and Quinlan, 2018). Furthermore, because the approach uses protein expression, the same method can be applied to study translational aspects of V1 plasticity in animal models and humans. The heuristic that we describe, a plasticity phenotype, will help for exploring and comparing neurobiological features that regulate experience-dependent development and plasticity of V1. The goal of constructing a plasticity phenotype is to take the unique computational signature obtained from high-dimensional analyses of proteins and transform it into a biologically interpretable plasticity phenotype for V1.

We describe a workflow with example code for constructing and using a plasticity phenotype by illustrating the steps in the statistical software R. The steps include a visualization tool that enhances the exploration of the data. The data sets used in the examples are from studies by our laboratory of V1 development and plasticity in cats (Beston et al., 2010; Balsor et al., 2019b), rats (Beshara et al., 2015) and humans (Murphy et al., 2005; Pinto et al., 2010, 2015; Williams et al., 2010; Siu et al., 2017). The examples address how to construct plasticity phenotypes for different experiments by discovering biological features in the data and using them to identify plasticity mechanisms that underpin differences among ages or rearing conditions.

### Contributions of This Paper

• We demonstrate how to combine measurements of plasticity-related proteins to construct and visualize a plasticity phenotype.
• We illustrate how to use the plasticity phenotype to rebrand the data to discover biologically meaningful interpretations of the data.
• We show how to use the plasticity phenotype to identify biological features that change during development, after different types of visual experience or after drug treatment.
• We aggregated all of the R code used in this paper into an R package “PlasticityPhenotypes” that is available for download using the devtools function: install_github(”visualneurosciencelab/PlasticityPheno types”).

The paper is organized as follows. First, we review some of the high-dimensional data analysis methods that have been used in recent papers studying cortical development. Next, we introduce the workflow to construct a plasticity phenotype and demonstrate how to use it with three examples: characterizing and comparing the development of V1 in cats and humans; classifying the effects of different types of treatment for abnormal early visual experience; identifying the effects of fluoxetine on adult rat V1. Finally, we provide a summary and discussion. This manuscript and a previous version have been released as Pre-Prints at bioRxiv (Balsor et al., 2019a, 2020).

### Past Work Using High-Dimensional Analysis

#### Principal Component Analysis

The most commonly used high-dimensional analysis for exploring gene or protein expression in the brain has been principal component analysis (PCA) (Hotelling, 1933; Jolliffe and Cadima, 2016). PCA transforms the data, which is likely to include correlated genes or proteins, into a linear set of uncorrelated principal components that capture successively less of the variance in the data. Thus, individual cases can be visualized and analyzed in the transformed lower-dimensional space, and that is often helpful for identifying clusters in the data. For example, a recent survey of human brain development used PCA to reduce the dimensionality of the protein and gene expression to identify differences among brain regions (Carlyle et al., 2017). That analysis separated cerebellar samples from other clusters, but the unitless dimensions of the PCA components made it hard to determine which biological features partitioned the samples into various clusters.

A different approach to using PCA takes advantage of known plasticity functions for a set of synaptic proteins. It uses the basis vectors for each component (the weights for each protein) to attach biological significance to otherwise unitless dimensions (Jones et al., 2007). For example, the information from the basis vectors may reflect biological features such as sums of proteins, balances between pairs of proteins or maturational states of protein families that are known to affect plasticity (Beston et al., 2010). The current workflow builds on that approach to using PCA.

#### t-Distributed Stochastic Neighbor Embedding

Another popular method for transforming and visualizing high-dimensional data is t-Distributed Stochastic Neighbor Embedding (t-SNE; Maaten and van der Hinton, 2008). tSNE measures the shortest distance between pairs of data points then calculates pairwise probability estimates of similarity across *all* dimensions. Often, these estimates are mapped onto a 2-dimensional (2D) space by scaling the distance between data points and positioning similar data points closer together. The new mapping preserves local and global patterns, thereby representing the relationships among data points to highlight clusters in the data. The artificial scaling makes it easier to identify clusters by either color-coding points based on a known attribute (e.g., cortical area), or by applying a clustering method to the tSNE XY coordinates. Furthermore, the unsupervised nature of tSNE is particularly useful when exploring data without strong *a priori* knowledge of the biological features that may differ among the conditions.

A recent study of single-cell mRNA expression in the developing human brain analyzed the data using a combination of PCA and tSNE (Nowakowski et al., 2017). In that example, PCA was used to reduce the dimensionality of the data and tSNE to reduce the dimensions further and visualize clusters. This has become a common workflow for analyzing and visualizing complex gene or protein data about brain development. Care is needed, however, when using the output from PCA as the input to a clustering algorithm because the orthogonal principal components (PC) may not contain the information needed to partition the data into clusters (Chang, 1983).

Whether clustering is done with PCA, tSNE or some other method, the same challenge remains for studying brain development and plasticity – how to link a holistic exploration of the data with the plasticity-related biological features that differentiate the clusters. The task of pinning down biological features is often done by presenting a large number of plots and univariate analyses aimed at finding individual proteins or genes that are over- or under-expressed in a cluster (Carlyle et al., 2017; Luo et al., 2017). That approach, however, loses sight of differences that arise from higher-order combinations of proteins or genes. The workflow presented here was developed to address that problem by using a series of steps for discovering combinations of proteins that represent high-dimensional features and a heuristic for analyzing the features that we call a plasticity phenotype. While the idea of brain phenotypes is not new (Cody et al., 2002), it has most often been used with brain imaging data, and the term plasticity phenotype has been used as a descriptor of gene changes during the CP (Smith et al., 2019). Our approach aims to construct a plasticity phenotype from neural protein expression data and use it to classify developmental and experience-dependent changes in V1.

## Methods and Results

### Note About the Preparation of the Data

Before beginning the analyses described in this paper, it is important to inspect and organize the raw data set. For example, if using Western blotting data, ensure that the quantification of the bands did not include artifacts (e.g., bubbles, spots) or poorly labeled bands that could skew the results. Those data points should be omitted, and the missing data can be filled by imputation. A variety of imputation functions have been implemented in R, and a package *impute* was developed for microarray data to impute missing gene or protein expression data using a nearest-neighbor analysis (Hastie et al., 2020).

### Description of the Example Data Sets

The data sets used for the examples in this paper come from our studies of V1 development and plasticity in cats (Beston et al., 2010; Balsor et al., 2019b), rats (Beshara et al., 2015) and humans (Murphy et al., 2005; Pinto et al., 2010, 2015; Williams et al., 2010; Siu et al., 2017). The workflow was tested on three different study designs including, small-N cross-sectional development studies, a small-N exploratory study of treatments after MD and a larger-N study examining the effects of fluoxetine on adult rats V1.

The cat data^1^ set has a maximum *nxp* matrix size of *n* = 768 rows of observations (24 cases X 16 tissue samples X 2 replications) and *p* = 7 columns of protein variables (Table 1). There were, however, some missing data, so the observed matrix had 3,906 data points, and the average protein expression from the western blotting runs was used, resulting in a matrix with 1,953 cells.

**TABLE 1:** Experiment observations (n) and variables (p).

**Data set**	**Categories**	**Specific**	**Total**
**Experiment observations (*n*)**			
Cat	Age (wks)	Normal (9): 2wk (1), 3wk (1), 4wk (1), 5wk (1), 6wk (1), 8wk (1), 12wk (1), 16wk (1), 24wk (1) Monocular Deprivation (8): 4wk (1), 5wk (2), 6wk (2), 9wk (2), 24wk (1) Treatment (7): Reverse Occlusion (RO) (1), Binocular Deprivation (BD) (1), Binocular Vision [Long-term, LT 1d, 2d or 4d, (3); Short-term, ST 1hr or 6hrs, (2)] (5)	24
	Regions	Central (2 or 3), Peripheral (6 to 11), Monocular (2)	10–16
	WB Runs	1, 2	2
		Sum	768
Human	Age (years)	0.05, 0.24, 0.26, 0.27, 0.33, 0.33, 0.36, 0.37, 0.75, 1.34, 2.16, 2.21, 3.34, 4.56, 4.71, 5.39, 8.14, 8.59, 9.13, 12.45, 13.27, 15.22, 19.21, 22.98, 32.61, 50.43, 53.90, 69.30, 71.91, 79.5	30
	WB Runs	1,2,3	2 or 3
		Sum	90
Rat	Rearing condition	Normal (6), 1wk MD (6), Fluoxetine +1wk MD (8), fluoxetine (8)	28
	WB Runs	1,2, 3	2 or 3
		Sum	84
**Variables (*p*)**			
Cat	Protein	Synapsin (Syn), GluN1, GluN2A, GluN2B, GluA2, GABA_*A*_α1, GABA_*A*_α3	7
Human	Protein	Synapsin (Syn), GluN1, GluN2A, GluN2B, GluA2, GABA_*A*_α1, GABA_*A*_α3	7
Rat	Protein	GluA2, GluN1, GluN2A, GluN2B, GABA_*A*_α1, GABA_*A*_α3, Gephyrin, PSD95, VGLUT1, VGAT	10

The human data^2^ set has a maximum *nxp* matrix size of *n* = 90 (30 cases X 3 replications) rows of observations and *p* = 7 columns of protein variables that are the same as the cat data set (Table 1). The average protein expression from the western blotting runs was used, resulting in a matrix with 210 data points.

The rat data^3^ set has a maximum *nxp* matrix size of *n* = 840 (28 animals total X 3 replications) rows of observations from the contralateral hemisphere and *p* = 10 columns of protein variables (Table 1). Some of the data points were omitted because of poor labeling resulting in a matrix with 770 data points.

### Constructing Plasticity Phenotypes to Describe V1 Development

**FIGURE 1:**
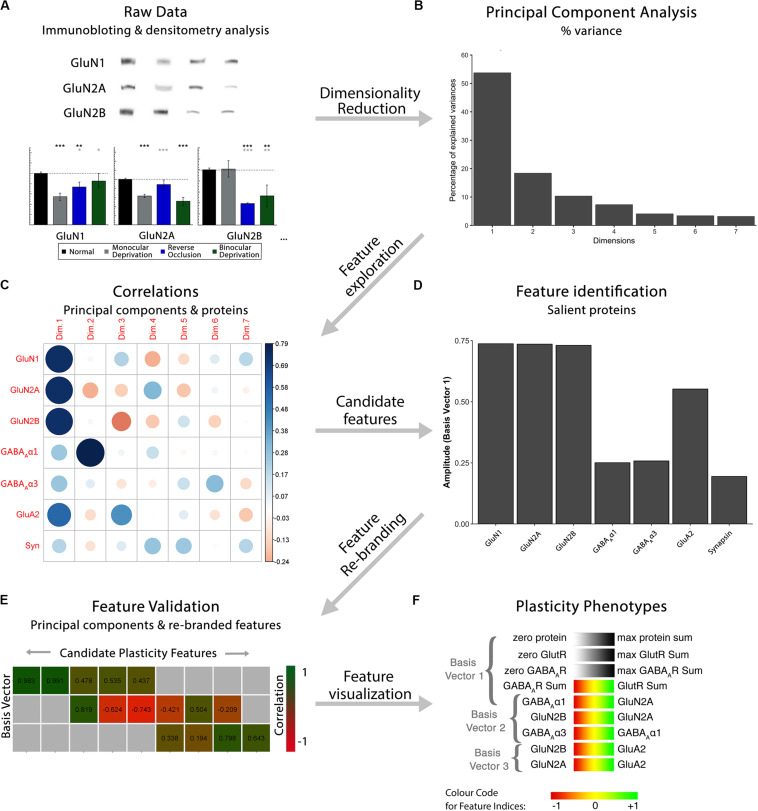
Analysis workflow to construct plasticity phenotypes. The analysis workflow for the data analysis described in the study [adapted from Balsor et al. (2019b), used with permission]. **(A)**. Immunoblots were quantified using densitometry, then comparisons among rearing conditions were made. Next, a series of steps were used to explore the data in a high dimensional space and create plasticity phenotypes. First, **(B)** dimensionality reduction (principal component analysis) was done on the centered data, followed by **(C)** feature exploration (correlations between principal components and proteins), **(D)** identification of candidate features (saliency), **(E)** feature rebranding (Correlation between principal components & features) and **(F)** ending with visualization of those features by creating plasticity phenotypes.

The development of plasticity mechanisms in V1 is often described using scatterplots and curve fitting to capture the trajectory of protein or gene expression changes with age. For example, with the current cat data set, that approach leads to 21 scatterplots and curves representing the 7 proteins and 3 sampling regions in V1. Even with that relatively small number of proteins, the number of possible trajectories multiplies, making it difficult to describe an overall pattern for the development of V1. Furthermore, that approach does not realize the potential of high-dimensional data since it does not include the full repertoire of proteins. Instead, holistic approaches that examine all proteins can identify patterns in the data that suggest how the biological functions might change. To address this combinatorial problem, we developed a workflow that reduces the dimensionality of the data set (Figures 1A,B), explores and identifies biological features contributing to variance in the data (Figures 1C,D), validates the features (Figure 1E) and uses those features to construct a plasticity phenotype (Figure 1F). First, the workflow is described using data for the development and recovery of cat V1 (Beston et al., 2010; Balsor et al., 2019b) and then extended to data for the development of human V1 (Murphy et al., 2005; Pinto et al., 2010, 2015; Williams et al., 2010; Siu et al., 2017) and fluoxetine induced changes in adult rat V1 (Beshara et al., 2015).

### Dimension Reduction Using PCA

**FIGURE 2:**
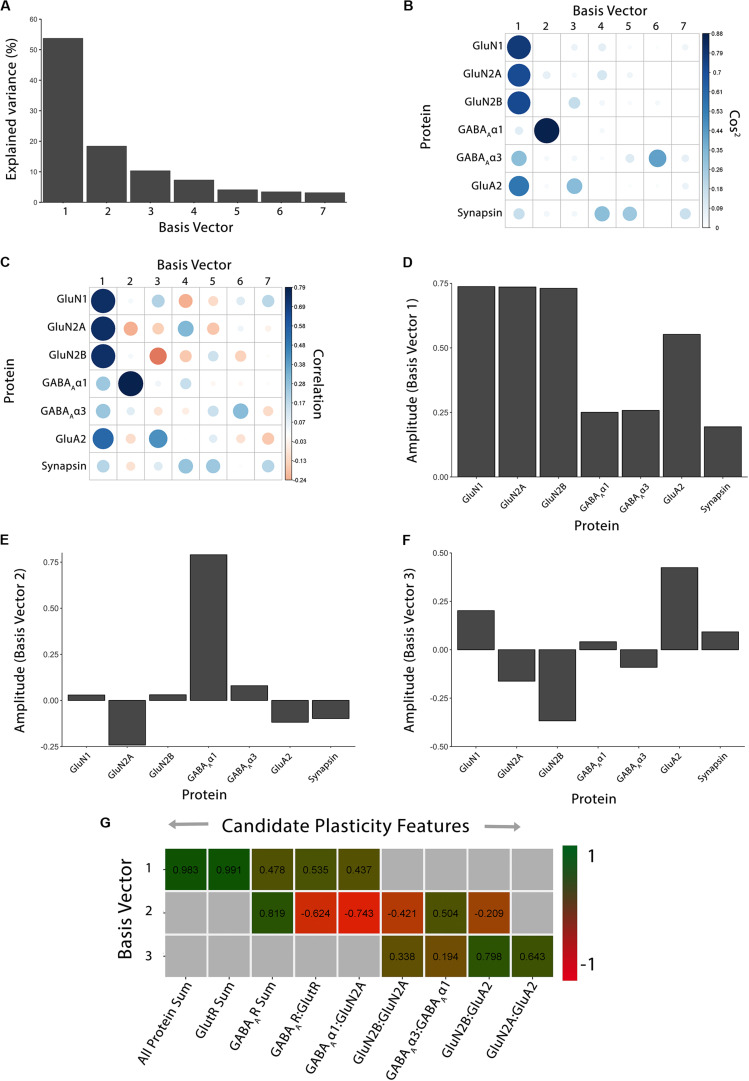
Using principal component analysis to identify candidate plasticity features. **(A)** The explained variance captured by each principal component after the singular value decomposition (SVD). The first three principal components capture 54, 18, and 10% of the variance, respectively, totaling >80% and thus representing the significant dimensions. **(B)** The quality of the representation, cos^2^, for the proteins is plotted for each dimension (small/white: low cos^2^; large/blue: high cos^2^). **(C)** The strength (circle size) and direction (blue-positive, red-negative) of the correlation (*R*^2^) between each protein and the PCA dimensions. **(D–F)** The basis vectors for dimensions 1–3 showing the amplitude of each protein in the vector. **(G)** Correlations between the plasticity features (columns) identified using the basis vectors (see Results) and PCA dimensions 1–3. Filled cells are significant, Bonferroni corrected correlations (green = positive, red = negative). Adapted from Balsor et al. (2019b), used with permission.

The first step in the workflow uses PCA to explore the high-dimensional nature of the data. We implemented a two-step procedure that reduced the dimensionality of the data and then used the basis vectors for those dimensions to identify candidate biological features that capture the variance in the data. Figure 2 illustrates the workflow using the cat data set (Beston et al., 2010; Balsor et al., 2019b), and the R code can be found in the Markdown file Cat&Human_Markdown in the Supplementary Material.

To start the analysis, load relevant packages and data files (Cat&Human_Markdown, section “Load Cat and Human Data,” lines 5–186) including installing the package *PlasticityPhenotypes* from the Visual Neuroscience Lab GitHub repository (Cat&Human_Markdown, section “Install the package, ‘PlasticityPhenotypes.’,” lines 8–9).

devtools::install_github(”visualneuro sciencelab/PlasticityPhenotypes”)

Next, load the package into the library (Cat&Human_Markdown section “Load the package, ‘PlasticityPhenotypes.’,” lines 10–54).

library(PlasticityPhenotypes)

Then, import the data sets of interest by setting the destination file paths for the CSV files (Cat&Human_Markdown section “Store the cat and human file paths in unique objects.”, lines 55–60).

raw.cat.dev <-
’https://osf.io/45rjy//?action=download’
raw.cat.tsne <-
’https://osf.io/59yu6//?action=download’
raw.hum.imputed <-
’https://osf.io/6pbgr//?action=download’
raw.hum.non-imputed <-
’https://osf.io/azdv8//?action=download’

Finally, import the CSV files from the Visual Neuroscience Lab Open Science Framework (OSF) repository for the cat (Cat&Human_Markdown section “Import the necessary cat data CSVs from OSF.”, lines 61–127) and human data (Cat&Human_Markdown section “Import the necessary human data CSVs from OSF.”, lines 128–186).

#### Cat Data Files

filename <- ‘cat_proteins.csv’
GET(raw.cat.dev, write_disk(filename, overwrite = TRUE))
raw.data <- read.csv(filename)
filename <- ‘cat_tsne.csv’
GET(raw.cat.tsne, write_disk(filename, overwrite = TRUE))
tsne.raw.data <- read.csv(filename)

#### Human Data Files

filename <- ‘human_imputed.csv’
GET(raw.hum.imputed, write_disk(filename, overwrite = TRUE))
raw.data.imputed <- read.csv(filename)

filename <- ‘human_non-imputed.csv’
GET(raw.hum.non-imputed, write_disk(filename, overwrite = TRUE))
raw.data.non-imputed <- read.csv(filename)

### Cat Analysis

Analysis of the cat data (Cat&Human_Markdown, Cat Analysis lines 187–557) began with the PCA (Cat&Human_Markdown, section “Cat: Dimension Reduction Using PCA – Additional Processing & Analysis,” lines 188–310). The column headers that include special characters were renamed, so those characters could be used in the figures (Cat&Human_Markdown section “Rename columns in the ‘raw.data’ object…,” lines 190–222). Here, the subscript (_A_) and Greek letter alpha (α) were represented with Unicode text in GABA_*A*_α1 and GABA_*A*_α3.

colnames(raw.data)[13:14] <- c(’GABA\u1D00\u03b11’, ‘GABA\u1D00\u03b13’)

The original data frame (Cat&Human_Markdown section “Assign ‘raw.data’ to the object ‘my.data.’,” lines 223–256) was duplicated to preserve the raw data set (raw.data), and have a second set of the raw data (my.data) to be preprocessed for PCA (see below).

my.data<- raw.data

Note: many implementations of PCA do not work well when there are empty cells in the matrix. There are a variety of approaches that can be used, including imputation to fill in the empty cells, removing runs with missing data, or averaging across runs. In this example, protein expression was averaged across multiple runs of western blotting, so there were no missing values.

This section is not an overview of PCA, and we encourage readers to go to online tutorials to learn more about the application of PCA to biological data. It is important, however, to emphasize that our use of PCA is a data-driven approach to understanding V1 development because *only* protein expression was used, and no categorical information such as treatment condition, cortical area, or age were included in the PCA.

The first step for performing the PCA was to center the data, but the data were not scaled. It is important to note that for many applications, the data are centered and scaled so that genes or proteins with abundant expression do not obscure the contribution of those with less expression but a significant variation. When scaling is added, the data will have a standard deviation of ± 1, and a mean of zero.

Centering the data in R was done with the base scale function and stored in the new data frame my.data.scaled (Cat&Human_Markdown section “Centre (but do not scale) the protein columns.”, lines 273–277).

my.data.scaled <- scale(my.data[,10:16],
center = TRUE,
scale = F)

There are a variety of PCA packages in R, and here, we used the *PCA* function from the *FactoMineR* package (Lê et al., 2008; Husson et al., 2020). The *PCA* function produces eigenvalues and a large set of visualization tools to aid the exploration of the data set and identification of plasticity features described in the next section.

PCA was run on the data set my.data.scaled, and the results were saved as the object pca.scaled (Cat&Human_Markdown section “Perform a PCA on ‘my.data.scaled’,” lines 278–282).

pca.scaled <- PCA(my.data.scaled,
ncp=ncol(my.data.scaled),
scale.unit=FALSE,
graph = FALSE)

Principal components returned by that function are the set of orthogonal vectors in the object pca.scaled that identify the variance in my.data.scaled. The eigenvalues represent the magnitude of the variance captured by each PC vector, and eigenvalues are largest for PC1 and successively less for each subsequent PC. An in-depth explanation of PCA and eigenvectors can be found here (Jolliffe and Cadima, 2016).

Dimension reduction began by identifying how much variance was captured by each PC, then ranking the PCs from largest to smallest, and lastly, retaining the set of PCs that captured a significant amount of the variance (>80%). The scree plot represents the amount of variance explained by each of the PC dimensions. The following code (Cat&Human_Markdown section “Construct scree plot,” lines 283–298) was used to consult the pca.scaled object to create a scree plot.

fviz_eig(pca.scaled,
addlabels = T,
ylim = c(0, 60),
xlim = c(0.5,7.5),
ncp = 7,
barfill = “gray”,
barcolor = “gray”,
geom = “bar”)+
scale_y_continuous(expand = c(0,0))+
scale_x_discrete(expand = c(0,0))+
theme(axis.line.y = element_line(),
axis.line.x = element_line(),
panel.grid = element_blank())

The scree plot (Figure 2A) showed the decreasing magnitude of the variance explained by the seven PC vectors. A variety of methods have been used to identify significant dimensions (Hoyle, 2008), and here, we used the simple rule to retain successive dimensions until the cumulative variance explained was ≧ 80%. In this example, Dim1-3 explained 82% of the variance and those were used in the next steps to identify candidate plasticity features.

A custom function cum_var was used to calculate the number of significant basis vectors for the subsequent analyses and that function is included in the *PlasticityPhenotypes* package (Cat&Human_Markdown section “Calculate how many components are required to maintain 80% of the total variance…,” lines 299–310).

pca.scaled$eig[,3]
## comp 1 comp 2 comp 3 comp 4 comp 5 comp 6 comp 7
## 53.66586 72.00511 82.27410 89.51460 93.55403 96.92361 100.00000
cum.var <- cum_var(pca.eig.3 = pca.scaled$eig[,3], # “pca.scaled$eig[,3]” is contained within object “pca” thresh = 80)
# Custom threshold value
cum.var
## [1] 3

### Identifying Candidate Plasticity Features

The three significant PC vectors represent the weighted contribution from each of the seven proteins to the variance in the data, and that information was visualized by plotting the cos^2^, correlations or individual basis vectors (Figures 2B–F; Cat&Human_Markdown section “Cat: Identifying candidate plasticity features,” lines 311–373). Those analyses identified the proteins that drove the variance in the data, and those data were stored as XY coordinates in the pca.scaled object. Candidate plasticity features were identified using the output from the PCA, starting with the cos^2^ metric to assess the quality of the representation for each protein on the dimensions (Cat&Human_Markdown section “Construct plot of Cos^2^ data …,” lines 312–329).

corrplot(pca.scaled$var$cos2,is.corr
= FALSE)

For the cat V1 data set, basis vector 1 had strong cos^2^ representations for the glutamatergic proteins GluN1, GluN2A, GluN2B and GluA2, and moderate for GABA_A_α3, and basis vector 2 had strong cos^2^ representation for GABA_A_α1 (Figure 2B). However, synapsin was weakly represented by all of the basis vectors (Figure 2B).

Next, the correlations between individual proteins and the basis vectors were plotted to visualize the strength and direction of the impact of the proteins on the vectors (Figure 2C; Cat&Human_Markdown section “Construct plot of coord (correlation) data…,” lines 330–348).

corrplot(pca.scaled$var$coord,
is.corr = F)

Lastly, amplitude plots for the three significant PC dimensions were plotted to visualize the weight and direction that each protein contributed to the basis vectors (Figures 2D–F). The amplitude plots present the same data as the correlation plot but make it easier to assess the amplitude of each protein. The custom function amplitude_plots included in the *PlasticityPhenotypes* package produces a series of plots demonstrating the amplitude of the variables (e.g., proteins) about each basis vector (Cat&Human_Markdown section “Create PCA amplitude plots…,” lines 349–373).

pca.scaled$var$coord
amplitude_plots(cum.var = cum.var,
# Output of ”cum_var()” function
pca.var.coord = pca.scaled$var$coord)
# ”pca.scaled $var$coord” is contained within object “pca”

### Heuristics for Identifying Candidate Plasticity Features

Candidate plasticity features were found by inspecting the basis vectors and applying three heuristics that combine information from PCA and *a priori* knowledge about the functions of the proteins in regulating plasticity. First, using cos^2^ and the basis vectors, we identified combinations of proteins representing the largest amount of variation. Second, *a priori* knowledge of V1 development and plasticity was used to find pairs of proteins (e.g., GluN2A:GluN2B, GluN2B:GluA2, GABA_A_α1:GABA_A_α3 (Chen et al., 2001; Philpot et al., 2001; Fagiolini et al., 2004; Hall and Ghosh, 2008; Smith et al., 2009) or classes of proteins (e.g., glutamatergic or GABAergic) that regulate plasticity by shifting the excitatory:inhibitory balance (e.g., E:I)(Fagiolini and Hensch, 2000; Hensch, 2004, 2005). Finally, novel pairs of proteins with large amplitudes that point in opposite directions (e.g., GABA_A_α1:GluN2A) were identified because the opposite directions suggest a potential functional link where one protein increases as the other decreases. Features were then made by summing proteins with high amplitudes or calculating difference indices for the pairs of proteins.

The supervised approach described here can be adapted to select appropriate features for new experiments by working through steps to develop a new set of heuristics. Alternatively, an unsupervised method, such as minimum Redundancy Maximum Relevancy (mRMR) (Ding and Peng, 2005), could be developed to find candidate features. That approach, however, would be strictly data-driven and may not select features most relevant to the neurobiological questions being addressed.

The following steps were used to identify candidate plasticity features for the cat V1 data set. Using the heuristics to identify proteins or combinations of proteins representing the largest amount of variance, we noted that on PC1, all of the proteins had positive weights and the glutamatergic receptor subunits had the largest amplitudes. Also, on PC2, GABA_A_α1 had the strongest representation. Together, that information suggested three candidate features: the sum of all seven proteins, the sum of the four glutamatergic proteins, and the sum of the two GABAergic proteins.

Next, applying the *a priori* knowledge heuristic, GluN2A and GluN2B, and GluN2B and GluA2 had opposite directions on PC2 and GABA_A_α1 and GABA_A_α3 on PC3. Besides, glutamatergic proteins were strongest on PC1 and GABAergic on PC2, suggesting orthogonal contributions to the variance in the data. Together, that information suggested four more candidate features: GlutR:GABA_A_R, GluN2A:GluN2B, GluN2B:GluA2, GABA_A_α1:GABA_A_α3.

Finally, novel pairs of proteins from PC2 and PC3 were identified. On PC2, GluN2A and GABA_A_α1 had the largest amplitudes and pointed in opposite directions. Across PC2 and PC3, GluN2A and GluA2 had the largest amplitudes pointing in opposite directions. That information suggested two candidate features: GABA_A_α1:GluN2A, GluN2A:GluA2.

### Candidate Plasticity Features for Cat V1

Applying the heuristics identified 9 candidate features (3 protein sums and 6 protein indices) (Cat&Human_Markdown section “Cat: Application of the Heuristics to Identify Candidate Plasticity Features for Cat V1” Lines 374–502). Those features were calculated and stored as separate columns in the NewFeatures data frame (Cat&Human_Markdown section “Calculate plasticity features,” lines 380–433).

The features were validated by determining the correlation between each of the nine candidate features and the three PC dimensions (Figure 2G; Cat&Human_Markdown section “Cat: Validating Candidate Plasticity Features,” lines 440–502). That was done by calculating the nine candidate features for all of the samples using the protein expression data (NewFeatures) and correlating those with the eigenvalues (coordinates) for the three dimensions (PCA.scores).

Bonferroni corrected Pearson’s correlations between the candidate features, and PC dimensions were calculated (note: False Discovery Rate could be used to adjust for multiple comparisons) (Cat&Human_Markdown section “Perform a Bonferroni-corrected, pairwise Pearson’s correlation against PCA scores and plasticity features.”, lines 443–457).

corr.scores.bf <- corr.test(PCA.scores[,1:cum.var], NewFeatures,
use = “pairwise”,
method = “pearson”,
adjust = “bonferroni”)

The correlation coefficients (Cat&Human_Markdown section “Store a matrix of correlation coefficients…,” lines 458–475) and p-values (Cat&Human_Markdown section “Store a matrix of adjusted *p*-values.”, lines 476–494) were stored as separate objects.

corr.scores.rval <- corr.scores.bf$r
corr.scores.bfpval <- corr.scores.bf$p

Finally, the significant correlations were visualized with a custom 2D matrix created using the custom function feature_matrix included in the *PlasticityPhenotypes* package. The function was used to visualize the significant correlations between the original PC basis vectors (PCA.scores) and the matrix of new features (NewFeatures) in a heatmap (Figure 2G, Cat&Human_Markdown line 502) (Cat&Human_Markdown section “Construct plasticity feature matrix.”, lines 495–502).

feature_matrix(
corr.scores.pval = corr.scores.bfpval,
# Matrix of adjusted p-values
corr.scores.rval = corr.scores.rval,
# Matrix of correlation coefficients thresh = 0.05)
# Significance threshold (acceptable values range from 0 – 1)

Figure 2G identified the validated features based on having a significant correlation between a candidate feature and one of the PC dimensions. Those features will be used in the next section to construct the plasticity phenotype. In the cat V1 example, all candidate features were correlated with at least one dimension, but none were correlated with all three dimensions.

### Using Plasticity Features to Visualize and Analyze the Plasticity Phenotype

The collection of plasticity features was combined to construct the *plasticity phenotype* (Cat&Human_Markdown sections “Cat: Using Plasticity Features to Construct a Plasticity Phenotype – Data Processing,” Lines 503–557; and “Cat & Human: Using Plasticity Features to Construct a Plasticity Phenotype – Creating Phenotypes,” Lines 741–812).

The first section of the R code (Cat&Human_Markdown sections “Cat: Using Plasticity Features to Construct a Plasticity Phenotype – Data Processing,” lines 503–557) prepared the data for normal cat V1 development to be used for constructing the plasticity phenotype. That included subsetting the normal data from the complete cat data set (lines 513–518), calculating the median feature value for each age and storing in a new data frame cat.dev (lines 523–528), ordering the rows sequentially by age (lines 529–540) and renaming and assigning the row names (lines 541–557).

merged.data <- subset(merged.data_all,
merged.data_all$Condition = = 1)

**FIGURE 3:**
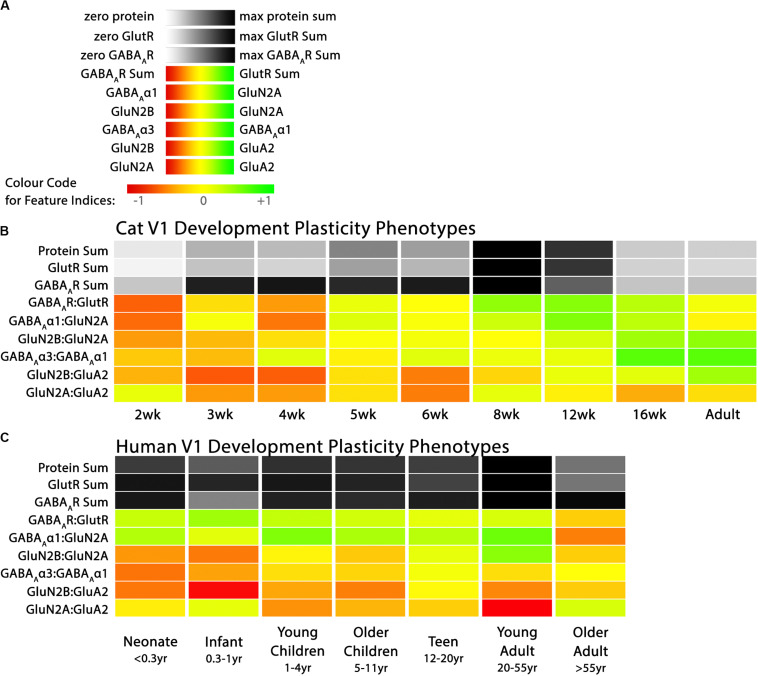
Plasticity phenotypes for cat and human V1 development. **(A)** The color-code for each of the nine plasticity features. **(B)** The developmental sequence of plasticity phenotypes in cat V1 was visualized as a stack of color-coded horizontal bars. The three gray-level bars represent the protein sums, and the six red-green color-coded bars represent the protein indices identified by the PCA. The plasticity phenotypes were calculated for each stage of cat V1 development and ordered from youngest to oldest [panels **A, B** reproduced from Balsor et al. (2019b), used with permission]. **(C)** The developmental sequence of plasticity phenotypes in human V1 was visualized as a stack of color-coded horizontal bars. The plasticity phenotypes were calculated for each stage of human V1 development and ordered from youngest to oldest. The color-coding conventions are the same as Figure 3B.

Figure 3 presents data for both cat and human V1 development, and the plasticity phenotypes for both were visualized using the R code in the Markdown section “Create Cat & Human Phenotypes” (lines 740–812).

First, the data were put into a list (df_list) that combined both the cat and human data (Cat&Human_Markdown section “Combine the plasticity feature columns in the human and cat data frames into a list…,” lines 743–746). Then the custom function plasticity_phenotype in the *PlasticityPhenotypes* package was called to process the data and make the phenotype visualization (Cat&Human_Markdown Section “Construct a plasticity phenotype …,” lines 747–812). That code made the sequence of V1 development phenotypes for both cats and humans (Figure 3).

plasticity_phenotype (df_list = df_list,
first_index_column = 4,
group_label = c(’\nAge Bins (Years)’,
’\nAge (Weeks)’),
translation = ‘absolute’)

**FIGURE 4:**
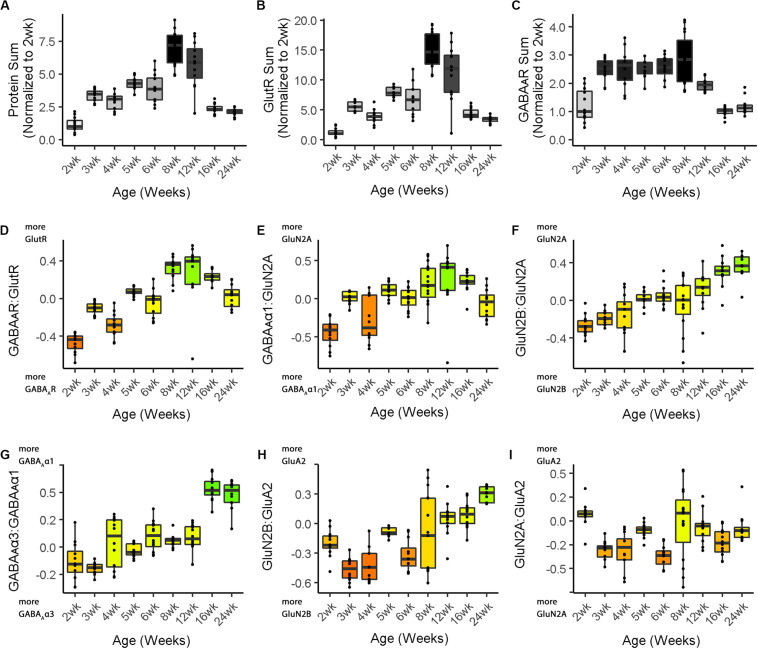
Boxplots of features included in the plasticity phenotypes for cat V1 development. The boxplots show the developmental progression for the nine plasticity features (**A–C**, protein sums) (**D–I,** indices) in cat V1. The color of each boxplot was set to match the corresponding feature bar in the cat V1 development plasticity phenotype (Figure 3B). Adapted from Balsor et al. (2019b), used with permission.

In addition to the phenotype visualization, boxplots were made to show the developmental changes for each feature (Cat&Human_Markdown section “Create Cat Boxplots,” lines 817–937) (Figure 4). The data were processed before creating the boxplots by parsing it to include the age identifiers (feats_df$Case) and normalizing the three protein sum features to the median of the youngest age. The boxplots present the changes relative to the youngest age (Cat&Human_Markdown section “Divide all data points in a ‘feats_df’ sum by the median value.”, lines 854–867).

sum1 <- feats_df[,’Protein Sum’]/
cat.dev[cat.dev$Group.1 = = “2wk”, ‘Protein Sum’]
sum2 <- feats_df[,’GlutR Sum’]/
cat.dev[cat.dev$Group.1 = = “2wk”, ‘GlutR Sum’]
sum3 <- feats_df[,’GABA\u1D00R Sum’]/
cat.dev[cat.dev$Group.1 == “2wk”,
’GABA\u1D00R Sum’]

The boxplots were created using the custom function phenotype_boxplots included in the *PlasticityPhenotypes* package (Cat&Human_Markdown section “Create individual boxplots color-coded according to the cat developmental phenotype.”, lines 886–928). The color-code from the phenotype visualization was used for the boxplots to facilitate comparison between the two ways of presenting the features.

phenotype_boxplots(feature_df = feats_df2,
# Boxplot data frame phenotype_cols = cat.cols,
# Phenotype colour-code data frame first_index_column = 4,
# Index number of first index column in “feats_df2” (indexes begin at 0) group_label = “\nAge (Weeks)”,
# X-axis label point_size = 0.6,
# Desired size of geom_jitter points point_alpha = 1,
# Desired transparency of geom_jitter points aspect_ratio = 5/7,
# Desired aspect ratio of each boxplot text_size = 8)
# Desired X- & Y-axis text size for each boxplot

Finally, the boxplots were arranged in a 3 × 3 grid (Cat&Human_Markdown section “Create a 3 × 3 matrix.”, lines 929–937).

ggarrange(plotlist = plot_list,
nrow = 3, ncol = 3,
labels = LETTERS[1:9],
font.label = list(size = 10),
vjust = 1)

### Translational Research Using Plasticity Phenotypes

One of the goals for developing the plasticity phenotype was to provide a common framework for comparing development and experience-dependent changes in V1 among species. Each of the features used to construct the plasticity phenotype from the cat data was derived from measurements of protein expression that can be readily measured in animal models as well as human postmortem tissue. As a result, the features can be standardized and compared among species. This section extends the analysis to compare the development of cat and human V1 and illustrates the application for translational research.

Using human V1 postmortem tissue samples, the nine plasticity features identified for the cat plasticity phenotype were applied to describe human V1 development. The human phenotype analysis used the code presented in the Cat&Human_Markdown (Supplementary Material). The CSV file with the Ages, Age bins and the seven proteins from the human V1 samples were loaded into the data frame object my.data (Cat&Human_Markdown section “Import the necessary CSVs from OSF,” lines 128–186).

Those data were used to analyze the same set of nine plasticity features identified for the cat (Cat&Human_Markdown section “Human: Analysis,” lines 558–739) and store them in the data frame NewFeatures.

The median values for each feature and age bin were determined (Cat&Human_Markdown section “Calculate the median of each plasticity feature…,” lines 708–714) and stored in hum.dev to be used as the input to the plasticity_phenotype function.

The plasticity phenotypes for human V1 development were created using the custom plasticity_phenotype function (Cat&Human_Markdown section “Create Cat & Human Phenotypes,” lines 740–812).

Comparisons of the cat and human development was facilitated by using the same red-yellow-green color map for both data sets. The numeric range for the color map was set using the “absolute” method (Markdown line 753) in the plasticity_phenotype function (see package documentation), that finds the largest absolute value using the medians from all of the age groups and assigns that value to green if positive and red if negative. For example, if the largest absolute value was +0.5, then the color scale will span from green = +0.5 to red = −0.5. That step ensured that zero on the indices was yellow and that the variations in green and red hues across the cat and human phenotypes represented the same numeric values on the indices.

Boxplots were made to show the development of the features for human V1 and each boxplot was color-coded using the same color map (phenotype_cols = hum.cols) as the phenotype visualization (Cat&Human_Markdown section “Create Human Boxplots…,” lines 938–1045).

phenotype_boxplots (feature_df = feats_df3,
# Boxplot data frame phenotype_cols = hum.cols,
# Phenotype colour-code data frame first_index_column = 4,
# Index number of first index column in “feats_df4” (indexes begin at 0) group_label = “\nAge Bins (Years)”,
# X-axis label point_size = 0.7,
# Desired size of geom_jitter points point_alpha = 1,
# Desired transparency of geom_jitter points aspect_ratio = 5/7)
# Desired aspect ratio of each boxplot

**FIGURE 5:**
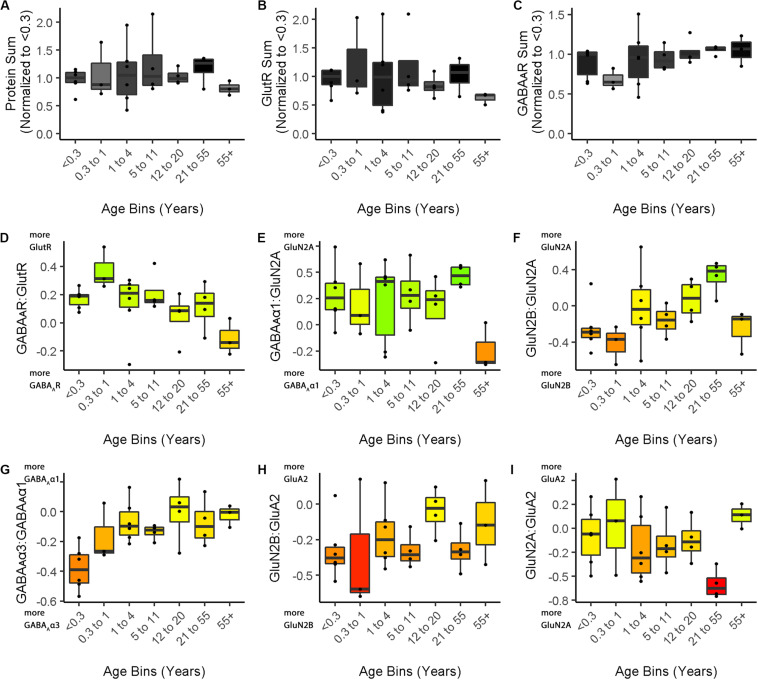
Boxplots of features included in the plasticity phenotypes for human V1 development. The boxplots show the developmental progression for the nine plasticity features (**A**–**C**, protein sums) (**D–I**, indices) in human V1. The color of each boxplot was set to match the corresponding feature bar in the human V1 development plasticity phenotype (Figure 3C).

The nine boxplots were arranged in a grid to create the multi-paneled Figure 5 (Cat&Human_Markdown section “Create a 3 × 3 matrix displaying all color-coded boxplots.”, lines 1037–1045).

ggarrange(plotlist = plot_list,
nrow = 3,
ncol = 3,
labels = LETTERS[1:9],
font.label = list(size = 12),
vjust = 1)

### Comparing Plasticity Phenotypes Between Species

Figure 3 illustrates the postnatal progression of plasticity phenotypes for cat (Figure 3B) and human V1 (Figure 3C). The boxplots show additional details about the magnitude of the nine features during development of cat (Figure 4) and human V1 (Figure 5). The set of gray-level and color-coded bands in the figures helped to identify sets of plasticity features with similar or different patterns of developmental change between cats and humans. Here, we provide a descriptive comparison for some of the similarities and differences in the pattern of the plasticity phenotypes because a comprehensive computational analysis is beyond the scope of this primer.

Differences in the maturity of V1 were apparent by looking at the plasticity phenotypes and comparing the three protein sum features represented by the gray-level bands. No doubt, the light-gray color of those three features in V1 of young kittens (2–4 weeks) was a reflection of the immaturity of the cortex at those ages. Neurons are still migrating into layers 2/3 of cat V1, thalamic afferents are just beginning to invade the cortical plate, and the layers have not fully differentiated (Luskin and Shatz, 1985; Shatz and Luskin, 1986). In contrast, those aspects of V1 development occur prenatally for humans (Flower, 1985; Kostovic and Rakic, 1990; Bhardwaj et al., 2006; Rakic, 2006; Clowry et al., 2010) leading to substantially more expression of synaptic proteins and the dark-gray color of the three protein sum features in the neonate and infant phenotypes.

The two indices that reflect balances between glutamatergic and GABAergic proteins (GlutR:GABA_A_R, GluN2A:GABA_A_α1) also suggest different patterns of development in cats and humans. In the kitten, those indices favor GABAergic proteins until ∼5 weeks of age when there was a shift to glutamatergic proteins. In neonates, however, those indices already favored glutamatergic proteins. The different maturational stages of the top five features suggest that human V1 is more mature than the young kitten and more similar to that of cats at the peak of the critical period (4–6 weeks of age). Examination of the next four features, however, suggests a different interpretation.

The next set of features appeared similar (orange and yellow hues) in young kittens and neonates and developed correspondingly. Those four features represent receptor subunit balances that are known to be affected by visual experience where the onset of vision drives the shift from more GluN2B to 2A (Quinlan et al., 1999a,b; Philpot et al., 2003; Beston et al., 2010; Jaffer et al., 2012; Balsor et al., 2019b), from more GABA_A_α3 to α1 (Huntsman et al., 1994; Hornung and Fritschy, 1996; Chen et al., 2001; Beston et al., 2010; Balsor et al., 2019b) and from more NMDARs to AMPARs (Rumpel et al., 1998; Beston et al., 2010; Funahashi et al., 2013; Balsor et al., 2019b). Those subunit balances also regulate a range of types of experience-dependent plasticity including cooperative, competitive, spike-time dependent, homeostatic and metaplasticity (Fagiolini et al., 2004; Hensch and Fagiolini, 2005; Philpot et al., 2007; Yashiro and Philpot, 2008; Cho et al., 2009; Gainey et al., 2009; Smith et al., 2009; Kubota and Kitajima, 2010; Larsen et al., 2010; Levelt and Hübener, 2012; Lambo and Turrigiano, 2013; Cooke and Bear, 2014; Guo et al., 2017; Hensch and Quinlan, 2018). The alignment of the bottom four features between young kittens and human neonates suggests that the onset of vision may initiate the maturation of some but not all experience-dependent plasticity mechanisms in V1. The trajectories, however, differ between the species with a range of developmental timescales for human V1 from early maturation for some features to much more prolonged development of other features stretching out across the lifespan. In contrast, the features in cat V1 matured at the peak of the critical period for ocular dominance plasticity.

### Clustering of Experience-Dependent Changes in V1 Using a Plasticity Phenotype

**FIGURE 6:**
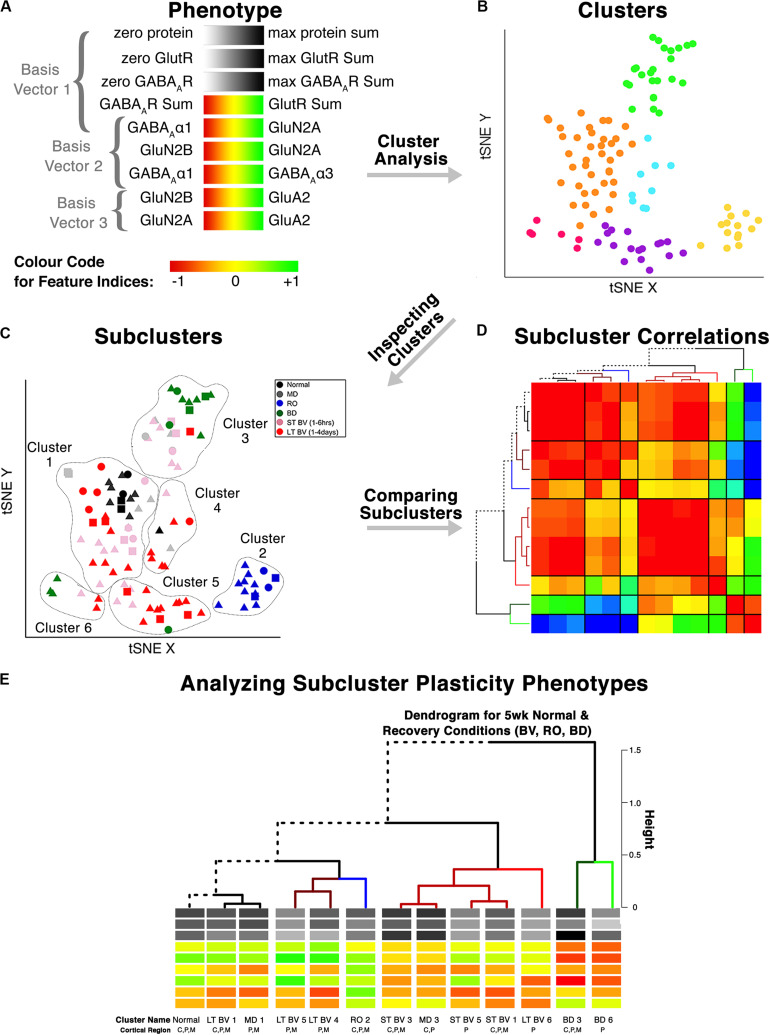
Analysis workflow to use plasticity phenotypes in cluster analysis. The analysis workflow is shown for using the plasticity phenotypes to identify clusters and subclusters [adapted from Balsor et al. (2019b), used with permission]. **(A)** The color-code for each of the nine plasticity features. The nine plasticity features were used as the input for the tSNE analysis, and k-means clustering was applied to the transformed data. **(B)** The tSNE identified clusters are color-coded. **(C)** The composition of the clusters was inspected by coding the rearing conditions using different colored symbols. **(D)** Samples from the same rearing condition and cluster are divided into subclusters, and the strength of the pairwise correlations among the plasticity phenotypes is shown using a correlation matrix. **(E)** The plasticity phenotypes for each subcluster were displayed at the ends of the dendrogram that ordered the subclusters **(D)**.

In this section, we extend the phenotype workflow to clustering V1 protein expression from the cat data set for animals reared with different types of visual experience (Figure 6). The approach is useful when using a data-driven approach to address questions about experience-dependent changes in V1 because only the protein or gene expression is used for the clustering. Here, we illustrate the steps using protein expression data from cat V1 (three regions: central, peripheral, monocular) of animals that had different types of visual manipulations (reverse occlusion RO, binocular deprivation BD or binocular vision BV) to promote recovery from early monocular deprivation (MD) (Balsor et al., 2019b). We selected this data set because the three different types of treatments lead to only two visual outcomes. Treatment with either RO or BD leads to very poor visual acuity in both eyes (Murphy and Mitchell, 1986, 1987; Duffy et al., 2015). In contrast, merely opening the deprived eye and allowing binocular vision can lead to the recovery of good acuity in the deprived eye with minimal effect on the acuity of the non-deprived eye (Murphy et al., 2015; Williams et al., 2015). Our goal for using cluster analysis with these data was to reduce the sample space and determine which recovery paradigms clustered with or near normally reared animals.

This application of the plasticity features and phenotypes used tSNE analysis to partition the data into clusters. tSNE preserves both the global and local arrangement of the plasticity features and is a good way to visualize clusters because it artificially scales the distance between data points with similar patterns of features. There are, however, many other clustering methods that could be used for this step, and the selection of the most appropriate clustering algorithm will depend on the structure of the data.

**FIGURE 7:**
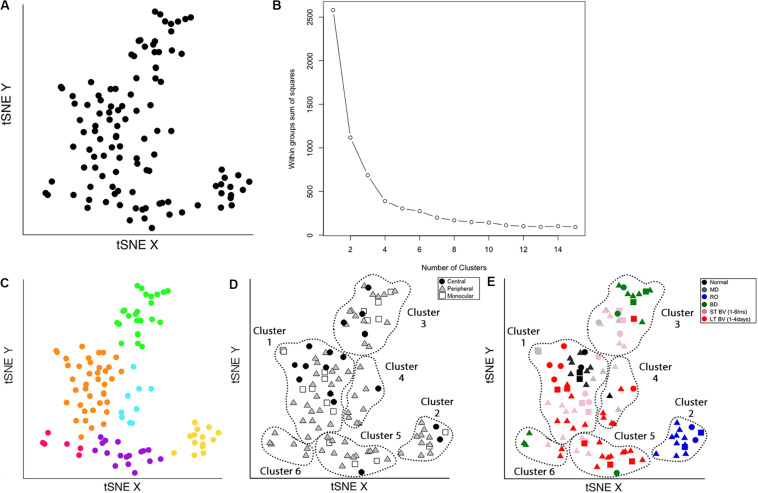
Identifying clusters from plasticity phenotypes using t-Distributed Stochastic Neighbor Embedding (t-SNE) and *k*-means clustering. **(A)** An XY representation of tSNE transformed data. **(B)** The optimal number of clusters was chosen by measuring the within-groups sum of squares, and selecting the inflection point (*k* = 6). **(C)** The six clusters are represented as different colored dots. **(D)** The content of each cluster was visualized for the three cortical regions (central, peripheral, monocular) and **(E)** six rearing conditions (normal, MD, RO, BD, ST-BV, LT-BV). Panels **(C–E)** reproduced from Balsor et al. (2019b), used with permission.

The Rtsne function from the *Rtsne* package was used to do the analysis (Krijthe and van der Maaten, 2018). However, Rtsne uses a new random seed each time it is run, so the code in the Markdown Cat_tSNE_plots_Figure 7 reproduces the plots for the tSNE analysis in Figure 7 by loading the file cat_subcluster.csv and storing it as tsne.raw.data. (Cat_tSNE_plots_Figure 7, section “Load relevant packages and data files,” lines 6–108).

The following section of the R code provides an overview of the steps for the tSNE dimension reduction and cluster analysis of the cat recovery data.

First, the nine validated plasticity features were subset from the data frame (merged.data_all) to remove all of identifying information such as the cortical region, rearing condition, or age and the new data frame (merged.treatment.data) was used as the input to the tSNE analysis (Figure 7A).

merged.treatment.data <- subset(merged.data_all,
merged.data_all$Condition == 3)
### Use Rtsne function on the subsetted data to reduce the dimensionality of the data
tsne<- Rtsne(merged.treatment.data [,10:ncol(merged.treatment.data)],dims = 2, perplexity=25, verbose=TRUE, max_iter = 5000)
### Save the object tsne$Y as d_tsne_1 It contains two columns, an X and a Y, for the unitless tSNE dimensions that are stored in a new data frame (d_tsne_1) to be consulted for plotting.
d_tsne_1 <- as.data.frame(tsne$Y)
### Create the data frame called d_tsne_2 that has the identifier information and the new tsne X Y coordinates ready for making plots.
d_tsne_2 <- data.frame(merged.treatment. data[,1:9],d_tsne_1)

The XY coordinates from the tSNE analysis were used as the input to the next clustering steps. Both K-means and hierarchical clustering algorithms require the number of clusters (k) as a parameter. A good method for choosing the number of clusters is to measure the within-groups sum of squares (WSS) for a range of *k*, plot that information and determine the inflection point. There were nine rearing conditions in the cat V1 recovery data (e.g., normal, monocular deprivation, etc.), so a range for *k* of 2–15 clusters was used to encompass the number of conditions.

# Calculate the WSS from the d_tsne_1
data frame to be used as the input
to the elbow analysis for
selecting the number of clusters.
wss <- (nrow(d_tsne_1)-1)^∗^sum(apply(d_tsne_1,2,var))
for (i in 2:15) wss[i] <- sum(kmeans(d_tsne_1,
centers = i)$withinss)

The optimal number of clusters was selected by fitting an exponential decay curve to the WSS data then finding the number of clusters corresponding to the point where the curve plateaued (4τ) (*k* = 6). This approach is called the “elbow method,” where 4τ is the point of inflection, or elbow, of the curve (Figure 7B).

Next, K-means clustering for *k* = 6 was done on the output (d_tsne_1) from the tSNE analysis.

# Use kmeans to partition the data in d_tsne_1 data into 6 clusters.
kmeans.clusters = kmeans(d_tsne_1,6)
Cluster.Number <- as.factor(kmeans. clusters$cluster)
# Create the data frame called d_tsne_3 that adds the cluster identifier to the information in d_tsne_2 (tsne XY coordinates, treatment & region).
d_tsne_3 <- data.frame(d_tsne_2, Cluster.Number)

The clusters were identified in the tSNE plot with different colors (Cat_tSNE_plots_Figure 7 section “Cluster-Coded: Create a tSNE scatter plot where points are colored according to their cluster membership.”, lines 165–191) (Figure 7C). Some clusters (green and yellow) were spatially separated on the tSNE plot, while others (e.g., orange and blue) were adjacent.

Other characteristics of the data (e.g., V1 region, treatment condition) were also used to interrogate the clusters (Figures 7D,E). First, the samples in the tSNE XY plot were coded by the V1 region (Cat_tSNE_plots_Figure 7 section “Region-Coded: Create a tSNE scatter plot.”, lines 192–216) (Figure 7D). Then the samples were coded by the treatment condition and V1 region (Cat_tSNE_plots_Figure 7 section “Condition- & Region-Coded: Create a tSNE scatter plot.”, lines 217–247) (Figure 7E).

### Cluster Composition and Subcluster Identification

The number of samples in each cluster ranged from 5 (magenta) to 38 samples (orange). Each sample was annotated using the visual cortical region (central, peripheral, or monocular) (Figure 7D) and rearing condition (Figure 7E) to analyze cluster composition and determine if the clustering reflected one of those parameters. For example, cluster 2 contained samples from only one rearing condition (reverse occlusion), and cluster 1 contained almost all of the normally reared cases. Still, it also had samples from other rearing conditions. Thus, this step identified clusters and provided some evidence that the rearing condition was driving changes in the plasticity phenotypes. However, the tSNE clustering did not reveal which features from the phenotypes separated the samples into different clusters or grouped them into the same cluster.

Cluster composition was done by annotating each sample in the cluster using the sample ID (e.g., visual cortical region, and rearing condition). That step identified the rearing conditions and V1 regions that were partitioned into the six tSNE clusters and revealed subclusters based on the rearing condition and cortical regions (Supplementary Table 1).

A final note: In this workflow, dimensionality reduction and feature selection were performed before tSNE analysis and clustering. Although this is a common approach for analyzing high-dimensional data in neuroscience, it is important to remember that PCA preserves features with variance that is aligned with the orthogonal dimensions. Thus, features with more subtle but important variance away from the PCA dimensions will not be included in subsequent clustering (Chang, 1983).

### Exploring Subclusters Using the Plasticity Phenotype

In this section, we describe a method for analyzing and visualizing the subclusters defined by the rearing condition and V1 region using the features that comprise the plasticity phenotypes.

**FIGURE 8:**
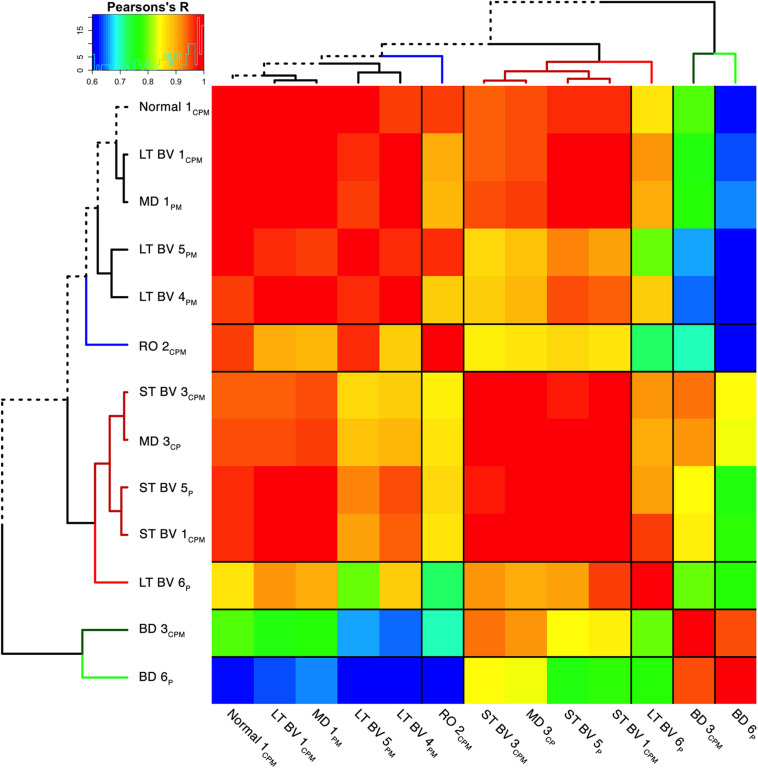
Visualizing pairwise correlations between recovery treatment subclusters. The matrix shows the strength of correlation (color) between the pairs of subclusters identified in the previous step [reproduced from Balsor et al. (2019b), used with permission]. Subclusters were ordered using hierarchical clustering to position subclusters with stronger correlations nearby in the matrix. This reordering identified five groups based on the height of the dendrogram branches and marked by different colored lines in the surrounding dendrogram. The solid black lines in the matrix mark the groupings of subclusters.

The nine plasticity features and tSNE results were combined in the file cat.correlation.csv that was loaded into the Markdown Cat_CorrHeatmap_Figure 8 (lines 75–111). Those data were used to calculate a Pearson’s correlation matrix using the subclusters (Cat_CorrHeatmap_Figure 8 section “Exploring Subclusters using Plasticity Phenotypes – Create Heatmap,” lines 131–200) (Figure 8). The order of the subclusters was determined using a dendrogram found by computing the distances for the correlation matrix and using that as the input to the hclust function illustrated in the following example code.

distance.row <- dist(as.matrix(CorMat), method = “euclidean”)
cluster.row <- hclust(distance.row, method = “complete”)
# Transform “cluster.row” into a dendrogram which is stored in “dd”.
dd <- as.dendrogram(cluster.row)

The correlation matrix for the plasticity phenotypes showed the strength of similarity or dissimilarity among the subclusters (Figure 8). Here, the surrounding dendrogram ordered some of the rearing conditions (e.g., LT BV) on the same branch as the Normal subcluster, while other conditions (e.g., BD) were far from the Normal branch. This analysis showed which subclusters had similar plasticity phenotypes but did not clarify if the similarity was based on the entire pattern of the plasticity phenotype or a subset of plasticity features.

### Constructing and Visualizing the Plasticity Phenotypes for the Recovery Treatment Subclusters

**FIGURE 9:**
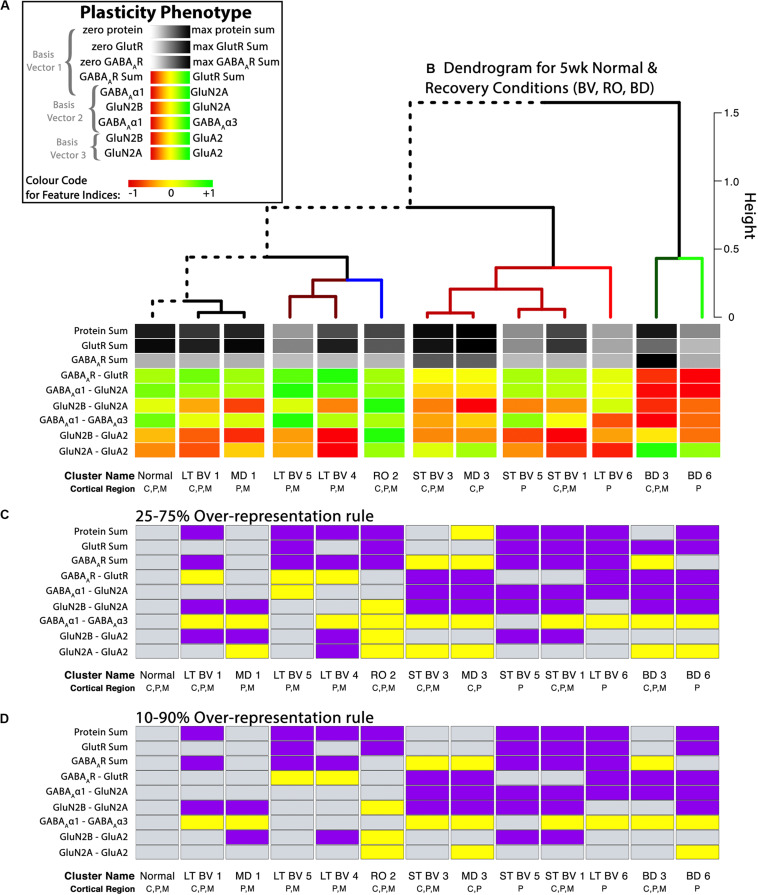
Analysis of plasticity phenotypes for the recovery treatment subclusters. **(A)** The color-code for each of the nine plasticity features. **(B)** The plasticity phenotypes for the 13 subclusters were ordered using the dendrogram that surrounds the correlation matrix in Figure 8. The rearing conditions (Normal, MD, RO, LT BV, ST BV) are described in Table 1. **(C)** Over-representation analysis (ORA) for each of the features in the treatment subclusters. With the first ORA (25–75%) bars were coded as over-represented (yellow) when the 25th percentile of the subcluster feature was greater than the 95th percentile of the normal distribution and under-represented (purple) when the 75th percentile of the feature was less than the 5th percentile of the normal distribution. That rule corresponds with the size of the boxplots in Figure 10. For the second ORA **(D)**, a more conservative rule was applied that used the 10th and 90th percentile to identify over- or under-represented features in the treatment subclusters. Panel A is adapted from Balsor et al. (2019b), used with permission.

In the last step for this workflow, we describe combining the plasticity phenotypes and hierarchical ordering of the subclusters to visualize the impact of the different types of visual recovery treatments (Figure 9). This step allowed for the direct comparison of the individual features as well as the complete plasticity phenotype for each subcluster.

Figure 9 shows the visualizations of the plasticity phenotypes for each subcluster and the identification of all the features that were over- or under-represented relative to normal. The panels in Figure 9 were made using code in the Cat&Human_Markdown section “Cat tSNE Analysis” (lines 1046–1426).

The subcluster labels and V1 regions for each sample were merged with the tsne.raw.data (Cat&Human_Markdown section “Cat tSNE Analysis,” lines 1052–1166). Then the median was calculated for each feature in the subclusters, and those were stored (cat.subcl) to be used for plotting the plasticity phenotypes for all of the subclusters (Cat&Human_Markdown section “Cat tSNE Analysis” lines 1174–1182).

cat.subcl_0.5 <- aggregate(tsne. processed.4 [,NewFeatCol],list(tsne. processed.4$Cluster.Name2), median)
### Store “cat.subcl_0.5” in “cat.subcl”. This was done to avoid potentially overwriting the contents of “cat.subcl_0.5”.
cat.subcl<- cat.subcl_0.5

Next, the plasticity phenotypes were visualized for the subclusters (Cat&Human_Markdown section “Cat: Constructing and Visualizing the Plasticity Phenotypes for the Subclusters” lines 1190–1275) (Figure 9B).

plasticity_phenotype(df_list = list(cat.subcl[,-1]),
# Median values data frame first_index_column = 4,
# Index number of first index column in “cat.subcl” (indexes begin at 0) group_label = “\nSubclusters”,
# X-axis labels translation = ‘local’)
# Desired colour-scale

Finally, an over-representation analysis (ORA) was used as a way of identifying features that might differ from the normal cluster. We used the ORA approach because it is a simple analysis that can be applied when a small-N design is used to discover features or rearing conditions of interest for follow-up studies. The adult fluoxetine study presented in the next section uses a larger-N design, and we use bootstrapping to estimate the uncertainty of the feature estimates and identify the ones that are likely to differ from normal.

A custom function in the *PlasticityPhenotypes* package was used to do an ORA and create the visualization (ORA_phenotype) (Cat&Human_Markdown section “Create an ORA_phenotype.”, lines 1356–1426) (Figure 9).

**FIGURE 10:**
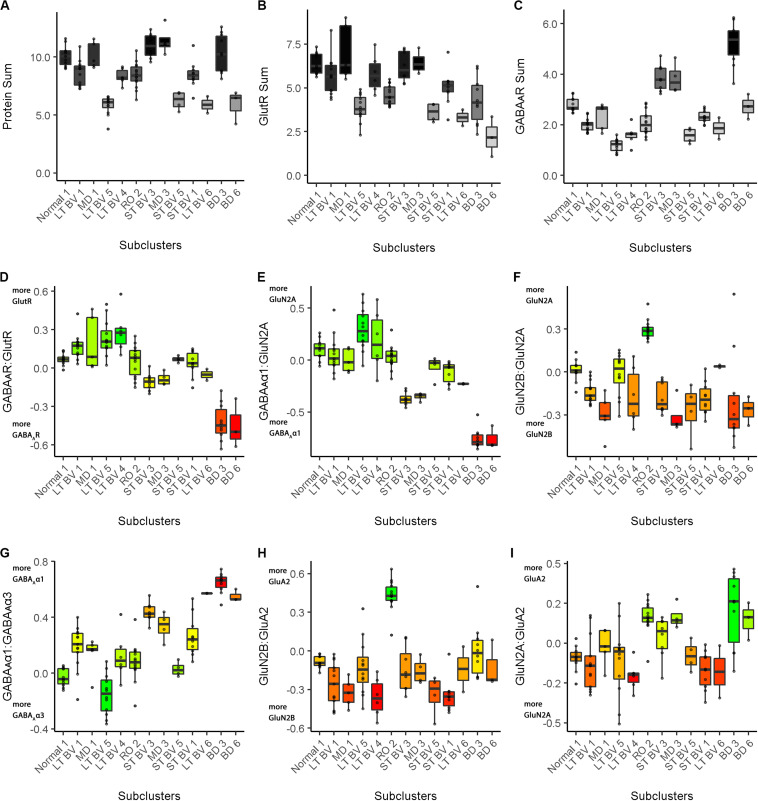
Boxplots showing the expression levels for each feature and recovery treatment subcluster. The boxplots show the nine plasticity features (**A–C**, protein sums) (**D–I**, indices) in cat V1 of the recovery treatment subclusters. The color of each boxplot was set to match the corresponding feature bar in the cat recovery plasticity phenotypes (Figure 9B). Adapted from Balsor et al. (2019b), used with permission.

The ORA_phenotype function simulated the distributions of each feature in the normal cluster and identified the 5th and 95th percentile values. Those values were compared with the features in the other clusters to determine over- or under-represented features. Here, we present two examples using the over-representation analysis. In the first example, a feature was coded as over-represented (yellow) if the 25th percentile of the subcluster feature was greater than the 95th percentile of the normal distribution and under-represented (purple) if the 75th percentile of the feature was less than the 5th percentile of the simulated distribution (Figure 9C). That rule corresponds with the size of the boxplots in Figure 10. Second, a more conservative rule was applied that used the 10th and 90th percentile of the features in the treatment subclusters to identify over- or under-represented features (Figure 9D). The comparison rule (e.g., 25–75% or 10–90%) can be passed to the function to evaluate different levels of uncertainty for describing the results.

# Create an ORA_phenotype using the object, “tsne.processed.4[,2:10]” with percentile values: “c(0.10,0.90)”.
ORA_phenotype(features_df_row = tsne.processed.4[,2:10],
# Data frame for bootstrap analysis
condition_list = as.list(
c(’Normal 1\n C,P,M’,
’LT BV 1\n C,P,M’,
’MD 1\n P,M’,
’LT BV \n P,M’,
’LT BV 4\n P,M’,
’RO 2\n C,P,M’,
’ST BV 3\n C,P,M’,
’MD 3\n C,P’,
’ST BV 5\n P’,
’ST BV 1\n C,P,M’,
’LT BV 6\n P’,
’BD 3\n C,P,M’,
’BD 6\n P’)),
# List of subclusters as they appear in row names of “features_df_row” reference_group = ‘Normal 1\n C,P,M’,
# Name of reference group as it appears in the row names of “features_df_row” group_label = “\nSubclusters”,
# X-axis label percentiles = c(0.10,0.90))
# Thresholds to use for experimental subclusters when performing ORA against

This example highlights the workflow’s application to a small-N approach where multiple proteins were measured for each animal and analyzed as part of a discovery process. This design can be useful when animal welfare is an issue, or the samples are rare and valuable (e.g., human postmortem tissue) (Editorial., 2020). The results are descriptive but still provide valuable information about treatment conditions that warrant further investigation. High-dimensional analyses are particularly useful for this type of small-N designs because the clustering algorithms *borrow strength* from the multiple variables to partition cases into clusters. In the current example, that strength supported partitioning of the rearing conditions into different subclusters, and the plasticity phenotypes for the subclusters helped to describe the neurobiological differences among the conditions.

### Interpretation of the Plasticity Phenotype and Cluster Analysis for Classifying Experience-Dependent Changes in V1

In addition to the plasticity phenotype visualization for the recovery data set (Figure 9), boxplots were made to facilitate understanding how each of the nine features varied by subcluster (Cat&Human_Markdown section “Cat: Constructing and Visualizing the Plasticity Phenotypes for the Subclusters,” lines 1288–1347) (Figure 10).

phenotype_boxplots(feature_df = tsne.processed.5[,-ncol(tsne. processed.5)],
# Boxplot data frame phenotype_cols = tsne.cols,
# Phenotype colour-code data frame first_index_column = 4,
# Index number of first index column in “tsne.processed.5[,-ncol(tsne. processed.5)]” (indexes begin at 0) group_label = “\nSubclusters”,
# X-axis label point_size = 0.7,
# Desired size of geom_jitter points point_alpha = 0.5,
# Desired transparency of geom_jitter points aspect_ratio = 9/10)
# Desired aspect ratio of each boxplot

Together, the visualizations presented in Figures 9, 10 help to describe the features that contributed to partitioning the rearing conditions into different subclusters. For example, RO and BD were partitioned into separate subclusters even though the two treatments lead to extremely poor acuity in both eyes (Murphy and Mitchell, 1986, 1987; Duffy et al., 2015). The red bands in the BD subcluster phenotypes describe the over-representation of GABA_A_Rs compared with GlutRs, and more GABA_A_α1 in particular. In contrast, the green bands in the RO phenotype point to over-representation of GlutRs compared with GABA_A_Rs and more GluN2A and GluA2. A notable finding was that even though the BV treatments clustered near the normal, none of the treatments returned all of the plasticity features to normal. Thus, even subtle differences in a feature contributed to partitioning the conditions into subclusters.

### Studying Fluoxetine-Enhanced Plasticity in Adult Rat V1 Using a Plasticity Phenotype

**FIGURE 11:**
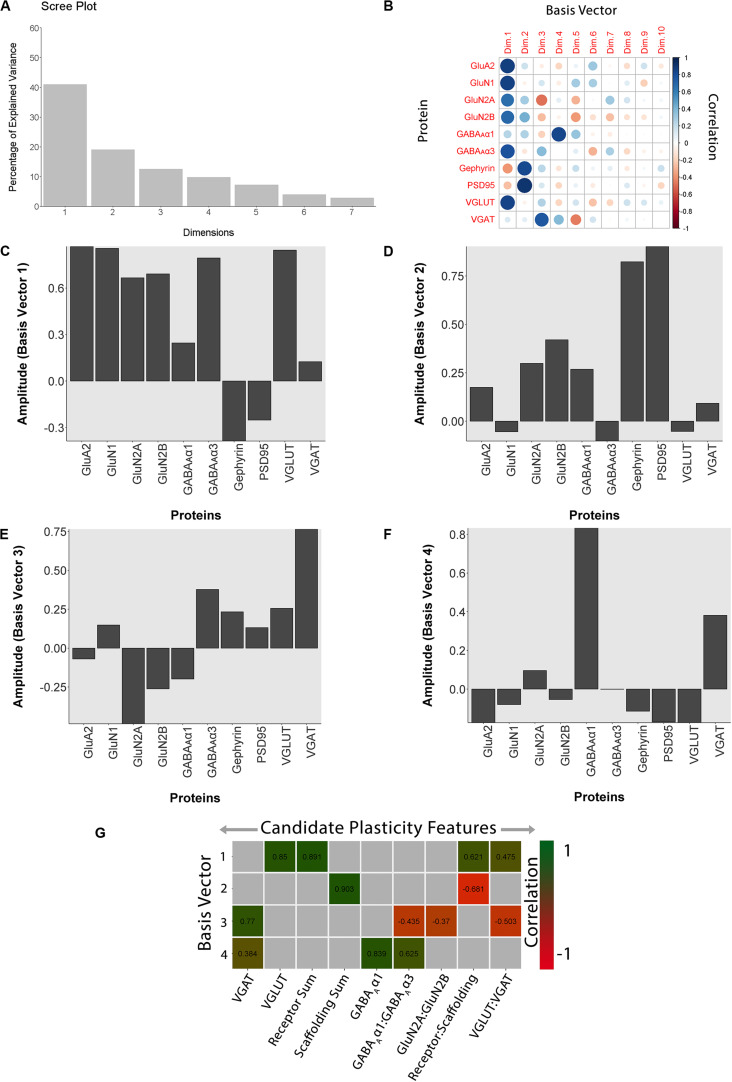
Using principal component analysis to identify candidate plasticity for rat fluoxetine data. **(A)** The explained variance captured by each principal component after the singular value decomposition (SVD). The first four principal components captured 50, 19, 13, and 10% of the variance totaling >80% and thus representing the significant dimensions. **(B)** The strength (circle size) and direction (blue-positive, red-negative) of the correlation (*R*^2^) between each protein and the PCA dimensions. **(C–F)** The basis vectors for dimensions 1–4 showing the amplitude of each protein in the vector. **(G)** Correlations between the plasticity features (columns) identified using the basis vectors (see section “Results”) and PCA dimensions 1–4. Filled and labeled cells are significant, Bonferroni corrected correlations (green = positive, red = negative).

In this example, we tested the workflow for analyzing experience-dependent changes in V1 using data from our study examining the effects of fluoxetine administration on the expression of glutamatergic and GABAergic proteins in adult rats (Beshara et al., 2015). Fluoxetine has been used to reinstate juvenile-like plasticity to adult rat V1 and promote visual recovery (Vetencourt et al., 2008). However, the mechanisms that support fluoxetine-enhanced plasticity remain poorly understood. In our previous study, we measured the expression of 12 synaptic proteins and assessed changes in V1 of adult rats that received either fluoxetine alone, MD alone or a combination of both treatments (Beshara et al., 2015). Figure 11 illustrates the application of the current workflow to those data showing the PCA findings for the data from the rat fluoxetine study. The analyses shown in Figure 11 follow the same steps described for Figure 2 in the previous section, “Constructing Plasticity Phenotypes to Describe V1 Development.”

The R code for this example is found in the Supplementary Material (Rat_Markdown and Rat_CorrHeatmap).

The scree plot from the PCA for the rat fluoxetine data showed that the first four PC dimensions explained >80% of the variance in the data (Rat_Markdown, section “Rat Analysis,” lines 86–157) (Figure 11A). Therefore, the basis vectors and cos^2^ for those four dimensions (Rat_Markdown, section “Rat Analysis,” lines 158–239) were interrogated to identify candidate plasticity features (Figures 11B–F). Here, we applied the three heuristics described in the section “Heuristics to Identify Candidate Plasticity Features.” Those heuristics include using cos^2^ and the basis vectors to select collections of proteins that account for the largest amount of the variance, *a priori* pairs of proteins known to regulate experience-dependent plasticity, and novel pairs of proteins with large weights pointing in opposite directions on a basis vector. That process identified nine candidate features that were then validated by determining the correlation between the basis vectors and the candidate features (Figure 11G). The significant features were identified by color-coding the cells green for positive and red for negative correlations (Rat_Markdown, section “Rat Analysis,” lines 240–355).

**FIGURE 12:**
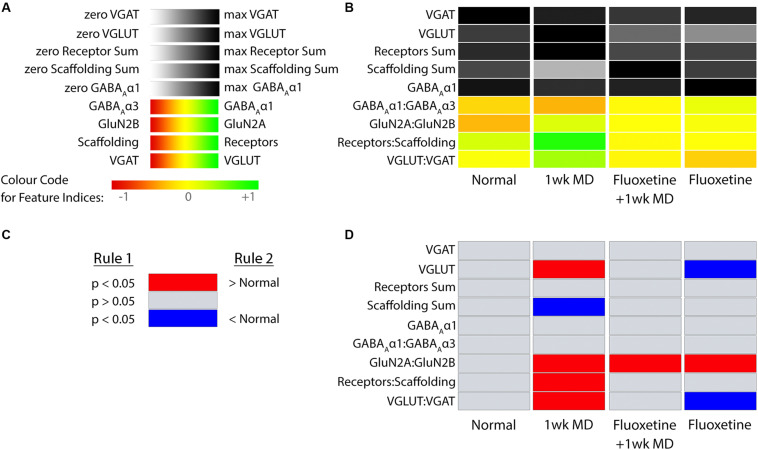
Plasticity phenotypes for rat fluoxetine data. **(A)** The color-code for each of the nine plasticity features identified for the rat fluoxetine study. **(B)** The color-coded plasticity phenotypes for each of the conditions in rat fluoxetine data (normal, 1wk MD, Fluoxetine +1wk MD, Fluoxetine). **(C)** The legend for the significance phenotype **(D)** where features were colored red if it was > normal and blue if it was < normal (*p* < 0.05). Significant differences were identified by color-coding the plasticity feature band. **(D)** The plasticity features for treatment groups that differed from normal **(B)** visualized as a stack of red/blue horizontal bars.

The nine plasticity features were used to construct a new plasticity phenotype for the rat fluoxetine data and compare among the four rearing conditions (Rat_Markdown, section “Rat Phenotype,” lines 401–424) (Figure 12A).

plasticity_phenotype(df_list = list(meds[,-1]),
# Median values data frame first_index_column = 6,
# Index number of first index column in “meds” (indexes begin at 0) group_label = “\nRearing Conditions”,
# X-axis label translation = ‘absolute’)
# Desired colour-scale

**FIGURE 13:**
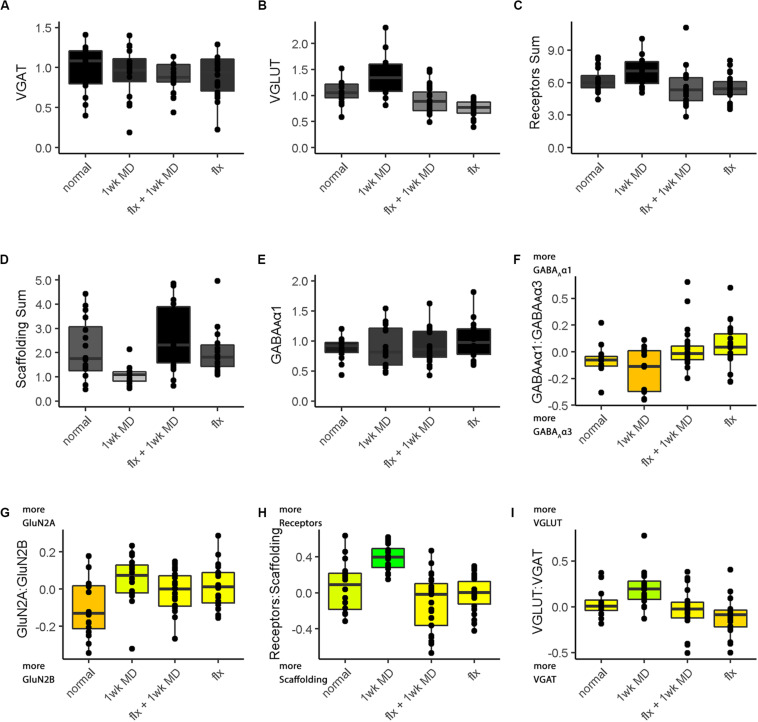
Boxplots of features included in the plasticity phenotypes for rat V1 fluoxetine treatment groups (Figures 12B,D). The boxplots show the protein sums **(A–E)** and index values **(F–I)** for each treatment group. The color of each boxplot was the same as the corresponding feature bar in the plasticity phenotype (Figure 12B).

Figure 12 shows the rat fluoxetine plasticity phenotypes for the four conditions and captures the findings from a large set of data that was originally presented using 16 separate graphs (Beshara et al., 2015). Interpretation of the plasticity phenotype visualization was facilitated by including boxplots for each feature (Figure 13) (Rat_Markdown, section “Create Rat Boxplots,” lines 427–498).

phenotype_boxplots(feature_df = merged.data2[,c(”Condition”, NewFeatsCol2)],
# Boxplot data frame phenotype_cols = rat.cols,
# Phenotype colour-code data frame max_sum = c(1.5, max(merged.data2$’VGLUT\n’), max(merged.data2$’Receptors Sum\n’), max(merged.data2$’Scaffolding Sum\n’),2),
# Maximum Y-axis values for feature sums’ boxplots group_label = “ ”,
# X-axis label first_index_column = 6,
# Index number of first index column in “merged.data2[,c(”Condition”, NewFeatsCol2)]” (indexes begin at 0) point_size = 1.5,
# Desired size of geom_jitter points point_alpha = 1,
# Desired transparency of geom_jitter points aspect_ratio = 5/7,
# Desired aspect ratio of each boxplot text_size = 8)
# Desired X- & Y-axis text size for each boxplot

The Bootstrap Analysis of the features (Rat_Markdown, section “Rat Plasticity Phenotype Bootstrap Analysis” lines 499–535) helped to identify that MD caused the most change from normal adult rats (Figure 12D). Five of the nine features were different from normal.

bootstrap_phenotype(features_df_row = NewFeatures[,NewFeatsCol],
# Data frame for bootstrap analysis condition_list = c( ’ normal ’, ’ 1wk MD ’, ’ flx + 1wk MD ’, ’ flx ’),
# List of experimental conditions as they appear in row names of “features_df_row” reference_group = ’ normal ’,
# Name of reference group as it appears in the row names of “features_df_row” group_label = “\nRearing Conditions”)
# X-axis label

For example, the features showed that MD increased VGLUT1 expression (darker gray bar) but reduced the amount of the receptor scaffolding proteins (PSD-95, Gephyrin) (lighter gray bar) (Figures 12, 13D). Also, the bottom three features of the MD condition shift to relatively more GluN2A, receptor subunits, and VGLUT1 that resulted in greener colors for those features (Figures 12, 13G–I).

The phenotype and bootstrap analyses helped to identify the effects of fluoxetine because all of the conditions and features were visualized in one figure. After fluoxetine +1wk of MD only one feature, the GluN2A:GluN2B index was different from normal, and the other four features changed by MD were not different from normal (Figures 12A,B, 13G). Thus, the addition of fluoxetine with MD resulted in the normalization of the majority of the plasticity features.

Finally, fluoxetine alone changed 3 of the 9 features, including reduced VGLUT1 (Figures 12A,B, 13B), and shifts to relatively more GluN2A (Figure 13G), and VGAT (Figure 13I) than normal. This visualization of all nine features in the phenotype helped to capture the fluoxetine-driven changes in favor of GABAergic mechanisms that have been described previously (Vetencourt et al., 2008).

To extend the high dimensional analysis of the rat fluoxetine data, we used the nine features from all of the animals (*n* = 28) as the input to an unsupervised hierarchical clustering algorithm (Ward.d2) described in a previous section (“Clustering of Experience-Dependent Changes in V1 Using a Plasticity Phenotype”). The R code for this example is found in the Supplementary Material (Rat_CorrHeatmap). Briefly, all pairwise Pearson’s correlations were calculated between the 28 animals and the order of the animals in the correlation matrix was determined by the surrounding dendrogram (Rat_CorrHeatmap, section “Studying Fluoxetine-enhanced Plasticity in Adult Rat V1…,” lines 195–247).

**FIGURE 14:**
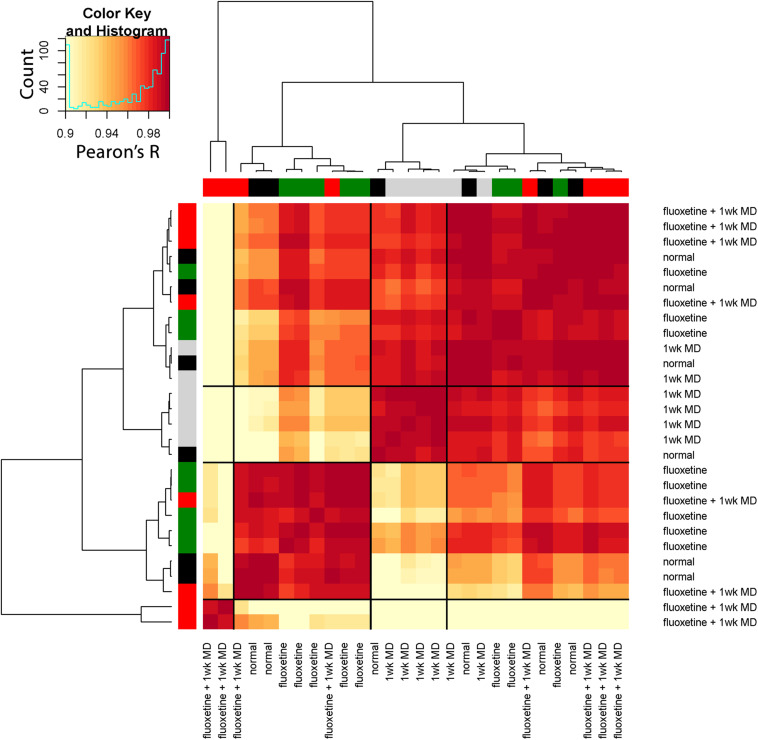
Visualizing pairwise correlations between treatments in the rat. The matrix shows the strength of correlation (color) between individual animals in each treatment group. Treatment groups were ordered using hierarchical clustering, and the resultant dendrogram positioned similar groups near one another. This reordering identified four clusters based on the height of the dendrogram branches, and the strength of the Pearson’s *R* correlations. The solid black lines in the matrix mark these four groups. The color at the end of each branch in the dendrogram indicates the rearing condition of the case: normal-black, 1wk MD-gray, fluoxetine + 1wk MD-red, fluoxetine-green.

The resulting correlation matrix highlighted four clusters in the rat fluoxetine data; however, the clusters did not merely recapitulate the four rearing conditions (Figure 14). The largest cluster had 12 animals that were from all of the rearing conditions suggesting that there is some overlap among the four conditions. Different rearing conditions dominated the other three clusters. The next clusters had 5 animals, and 4 were reared with 1wk of MD. The second-largest cluster had 9 animals, and 5 were reared with fluoxetine alone. Finally, there was a small cluster with just 2 animals that had fluoxetine combined with MD. Thus, using the plasticity features as the input to a cluster analysis clarified that 1wk MD and fluoxetine alone were most likely to shift the pattern away from normal.

## Discussion

Many neural proteins underpin the complex set of processes that support experience-dependent plasticity in V1. In this paper, we have provided a step-by-step primer using R code to illustrate a data-driven approach for combining measurements from multiple plasticity proteins to construct and use a plasticity phenotype for studying V1. The examples highlight the steps for rebranding protein measurements into plasticity features and combining those features into a phenotype. Thus, the plasticity phenotype can be thought of as a collection of biomarkers. We illustrated the use of plasticity phenotypes to classify tissue-level changes in V1 for different ages, rearing conditions, and species including cat, rat, and human. We also showed that the phenotype visualization tool helps synthesize large amounts of data into a single figure for exploring development and plasticity in V1. For example, the comparison of cat and human V1 development identified some features that follow similar postnatal patterns, while others have unique trajectories in humans. Thus, this application of plasticity phenotyping has the potential to guide discoveries about the experience-dependent development of V1 that can facilitate translation from animal models to humans.

We used the term “phenotype” to study a set of synaptic proteins that are known to regulate plasticity in V1 based on visual experience (Fagiolini et al., 2004; Hensch and Fagiolini, 2005; Philpot et al., 2007; Yashiro and Philpot, 2008; Cho et al., 2009; Gainey et al., 2009; Smith et al., 2009; Kubota and Kitajima, 2010; Larsen et al., 2010; Levelt and Hübener, 2012; Lambo and Turrigiano, 2013; Cooke and Bear, 2014; Guo et al., 2017; Hensch and Quinlan, 2018). Thus, the *plasticity phenotype* can be used to classify the state of plasticity mechanisms in V1. The term plasticity phenotype has been used previously to describe the waxing and waning of plasticity-related gene expression in V1 (Smith et al., 2019). Here, we applied a quantitative approach to phenotyping, and the traits observed were plasticity-related proteins measured by Western blotting that were rebranded into plasticity features (sums and indices). The collection of features was used to construct an *extended phenotype* (Dawkins, 1982) and infer V1 plasticity based on the measured features. For example, the GluN2A:GluN2B balance is known to regulate various aspects of experience-dependent plasticity in V1 (Smith et al., 2009). Thus, observing that balance describes a characteristic of V1 plasticity that contributes to an overall plasticity phenotype.

The paper’s examples show the application of a phenotyping approach to different experimental designs for discovery or hypothesis-testing of experience-dependent plasticity in V1. Exploratory small-N studies that use rare and valuable tissue samples, such as the cat and human V1 data sets, can apply the plasticity phenotyping approach to narrow in on new aspects of V1 development and generate hypotheses for testing in future studies. The high dimensional analyses are particularly useful for small-N discovery studies because the algorithms *borrow strength* across the multiple measurements of proteins or genes to increase the sensitivity and help estimate differences. The phenotyping approach also helps with larger-N designs like the rat fluoxetine study because the visualization unifies the results of many comparisons, thereby capturing complex changes in one figure that can be used to formulate new theories.

The workflows described in the paper were developed to help with interpreting complex data sets that include the expression of multiple proteins or genes at different ages, rearing conditions or species. Aspects of the workflows are similar to other approaches used to study V1 that applied sequential steps beginning with dimension reduction (e.g., PCA, tSNE) and then cluster analysis (e.g., Carlyle et al., 2017; Luo et al., 2017). The new workflows extend previous studies by presenting the steps to build, visualize, and compare plasticity phenotypes. Those steps can facilitate meaningful interpretation of intricate patterns of protein or gene expression in V1. For example, the analysis of the rat fluoxetine data set synthesized 16 graphs in the original paper (Beshara et al., 2015) down to just one figure. Furthermore, the visualization of plasticity phenotypes for the 4 rearing conditions highlighted the substantial change in adult rat V1 caused by MD and that adding fluoxetine normalized all of the features except the GluN2A:GluN2B balance.

### The Plasticity Phenotype Facilitates Translation

In addition to helping understand complex data, the plasticity phenotyping approach can enhance the translation of findings from model systems to humans. The example used in the paper compared cat and human V1 development because the cat is one of the model systems that laid the foundation for understanding experience-dependent changes in V1 and visual perception (Wiesel and Hubel, 1965a,b; Hubel and Wiesel, 1970). Those early studies included showing that NMDA receptors and GABAergic mechanisms participate in regulating that plasticity in V1 (Kleinschmidt et al., 1987; Gu et al., 1989; Hensch et al., 1998). The cat continues to be a helpful model for translation, especially for studies of development and amblyopia because it has a prolonged developmental period and good visual perception (Cnops et al., 2004, 2008; Beston et al., 2010; Duffy and Mitchell, 2013; Duffy et al., 2015; Laskowska-Macios et al., 2015, 2017; Williams et al., 2015; Balsor et al., 2019b; Mitchell et al., 2019). Here, we took advantage of that extended development and combined it with measurements of proteins and features that have been extensively studied in mouse models showing how they regulate V1 plasticity. The comparison revealed some similarities but other significant differences between the species. For the cat, the development of plasticity features proceeded in concert so that the whole phenotype reached a maximum at the height of the critical period for cat V1 (4–6 weeks in the cat). In contrast, the plasticity phenotypes for human V1 development encompassed multiple developmental timescales with collections of features maturing at different ages, from early changes during the neonatal period to a series of prolonged trajectories that cover the lifespan.

### Next Steps for Extending the Plasticity Phenotyping Approach

Since the current workflows build on established methods for high-dimensional data analysis, it is possible to adapt them to use other algorithms by exchanging a few lines in the R code. As new plasticity mechanisms are identified, those can be explored to find novel associations between visual plasticity and V1 neurobiology. The advent of single-cell transcriptomics using RNA sequencing to study brain development has added another layer of complexity. However, those very high-dimensional data studies have spurred the development of new analytical tools to discover gene markers for cell phenotypes (Aevermann et al., 2018). There is little doubt that further development of high-dimensional analyses will be necessary for decomposing the direct and indirect effects of changing patterns of plasticity proteins and genes on visual development. Adding measurements of visual function to the collection of features is a natural next step for this approach. However, vision and the neurobiology that underpins it are dynamic systems and changes at the neurobiological level may occur days, weeks, or months before vision changes are manifest. Thus, combining dynamic properties of molecular features with visual, physiological and anatomical measurements would capture multiple modalities and timescales to provide a richer phenotype for describing visual system plasticity.

### Feature Selection: Supervised Versus Unsupervised

The supervised approach to feature selection used in this study worked well with the example data sets, and it facilitated classifying patterns of changes in V1 during development and after abnormal visual experience. The approach took advantage of prior knowledge about the function of the plasticity proteins when selecting the features, and that knowledge facilitated making inferences about the plasticity state of V1. However, this approach needs to be tested with other data sets, especially in model systems, where the molecular mechanisms underpinning the features can be directly manipulated. Additional work is necessary to develop unsupervised methods for identifying features and transforming data. These changes will be especially important when working with very high dimensional data sets containing hundreds or thousands of proteins or genes and looking for new suites of plasticity features that may have a role in V1 plasticity. Other methods for solving the feature selection problem, such as minimum redundancy maximum relevance (mRMR) (Ding and Peng, 2005) or random forest machine learning (Aevermann et al., 2018), may be helpful for automatic annotation of patterns in the data. Finally, the public knowledgebase Synaptic Gene Ontologies (SynGO), which describes the function of synaptic genes, can be used to augment an unsupervised approach by selecting synaptic genes or proteins that match a term such as “plasticity” (Koopmans et al., 2019).

Similarly, an exploratory process and manual inspection of clusters were used in the clustering workflow to select the k-means parameters for the number of clusters. Estimating the number of clusters in a data set is challenging; however, methods such as the gap statistic can be added to the workflow for choosing the number of clusters (Tibshirani et al., 2001). Additionally, recent approaches to clustering, such as robust (weighted) sparse *k*-mean clustering, have the advantage of simultaneously identifying clusters and informative features for partitioning the data that can be used in feature selection (Brodinová et al., 2019). Finally, growth mixture models for cluster analysis of longitudinal data may be more suitable for data analysis from studies that include a series of sequential measurements of cortical development (Wei et al., 2017).

## Conclusion

The plasticity phenotypes described in this primer used the expression of a set of proteins to classify the neurobiological milieux of V1 during development and experience-dependent plasticity. In the long-term, the value of a phenotyping approach will be to show the composition of a set of observable molecular mechanisms that regulate experience-dependent plasticity. Here, the plasticity phenotype was used as an exploratory tool, but in future studies, it could be used to test which high-dimensional patterns of protein or gene expression are necessary to support the recovery of good vision. Those studies will require additional molecular and imaging tools to manipulate the expression and follow the dynamics of changes *in vivo*. Overall, the development of a phenotyping approach holds the potential of establishing a standard vocabulary with a formal ontology of plasticity features and phenotypes that can be used to classify things like developmental stages, critical periods and disease-related changes in V1.

## Data Availability Statement

The data used to support the findings of this study are available here: https://osf.io/8a3kx/ or from the corresponding author upon request. The code can be downloaded here: https://github.com/visualneurosciencelab/PlasticityPhenotypes.

## Ethics Statement

The studies involving human post-mortem tissue samples were reviewed and approved by the Hamilton Integrated Research Ethics Board HiREB #2605. Written informed consent for participation was not required for this study in accordance with the national legislation and the institutional requirements. The animal studies were reviewed and approved by the McMaster Animal Research Ethics Board (AREB) McMaster University.

## Author Contributions

JB designed and performed the research, analyzed the data, and wrote and revised the manuscript. DA performed the research, analyzed the data, and revised the manuscript. DJ designed the research, analyzed the data, and revised the manuscript. KM designed the research, analyzed the data, and wrote and revised the manuscript. All authors contributed to the article and approved the submitted version.

## Conflict of Interest

DJ was employed by the company Pairwise Affinity Inc. which has no commercial or financial relationship with the research.

The remaining authors declare that the research was conducted in the absence of any commercial or financial relationships that could be construed as a potential conflict of interest.

## Footnotes

1 https://osf.io/45rjy/?action=download

2 https://osf.io/6pbgr//?action=download

3 https://osf.io/d5pzt//?action=download
