# Supplementary material for: A Primer on Constructing Plasticity Phenotypes to Classify Experience-Dependent Development of the Visual Cortex
Source: Front Cell Neurosci. 2020 Aug 27;14:245. doi: 10.3389/fncel.2020.00245 (PMC7482673; doi:10.3389/fncel.2020.00245)
Supplement: Supplementary file 1 [file Table_1.DOCX]

| **Subcluster** | **Rearing Condition** | **tSNE Cluster** | **Region** | | |
| --- | --- | --- | --- | --- | --- |
|  |  |  | **C** | **P** | **M** |
| Normal 1_C,P,M_ | Normal | 1 | X | X | X |
| LTBV1_C,P,M_ | Long term BV recovery | 1 | X | X | X |
| MD1_P,M_ | Monocular deprivation | 1 |  | X | X |
| STBV1_C,P,M_ | Short term BV recovery | 1 | X | X | X |
| RO2_C,P,M_ | Reverse occlusion | 2 | X | X | X |
| STBV3_C,P,M_ | Short term BV recovery | 3 | X | X | X |
| MD3_C,P_ | Monocular deprivation | 3 | X | X |  |
| BD3_C,P,M_ | Binocular deprivation | 3 | X | X | X |
| LTBV4_P,M_ | Long term BV recovery | 4 |  | X | X |
| LTBV5_P,M_ | Long term BV recovery | 5 |  | X | X |
| STBV5_P_ | Short term BV recovery | 5 |  | X |  |
| LTBV6_P_ | Long term BV recovery | 6 |  | X |  |
| BD6_P_ | Binocular deprivation | 6 |  | X |  |

Supplementary Table 1: Description of the subclusters identified by the tSNE analysis.
