# Supplementary material for: A Primer on Constructing Plasticity Phenotypes to Classify Experience-Dependent Development of the Visual Cortex
Source: Front Cell Neurosci. 2020 Aug 27;14:245. doi: 10.3389/fncel.2020.00245 (PMC7482673; doi:10.3389/fncel.2020.00245)
Supplement: Supplementary file 2 [file Data_Sheet_1.pdf]

A primer on constructing plasticity phenotypes to classify experience-dependent development of the visual cortex

—

All R Markdowns Compiled

—

Table of Contents

Markdown Number	Title	Page Number (of 108)
1	Cat&Human_Markdown	2 of 108
2	Cat_tSNE_plots_Figure7	52 of 108
3	Cat_CorrHeatmap_Figure8	63 of 108
4	Rat_Markdown	71 of 108
5	Rat_CorrHeatmap_Figure14	98 of 108

Cat&Human_Markdown

Table of Contents

Section Number	Title	Page Number
1	Load Cat and Human Data	2
2	Cat Analysis	7
3	Human Analysis	24
4	Create Cat & Human Phenotypes	29
5	Create Cat Boxplots	32
6	Create Human Boxplots	36
7	Cat tSNE Analysis.	40

1.) Load Cat and Human Data

Cat & Human: Dimension Reduction Using PCA – Data Processing

Install the package, "PlasticityPhenotypes".

```
devtools::install_github("visualneurosciencelab/PlasticityPhenotypes")
```

Load the package, "PlasticityPhenotype".

```
library(PlasticityPhenotypes)

## Loading required package: tidyverse

## -- Attaching packages ---------------------------------------------------------
----------------------- tidyverse 1.3.0 --

## v ggplot2 3.3.0     v purrr   0.3.3
## v tibble  2.1.3     v dplyr   0.8.5
## v tidyr   1.0.2     v stringr 1.4.0
## v readr   1.3.1     v forcats 0.5.0

## -- Conflicts ------------------------------------------------------------------
----------------- tidyverse_conflicts() --
## x dplyr::filter() masks stats::filter()
## x dplyr::lag()    masks stats::lag()

## Loading required package: FactoMineR

## Loading required package: factoextra

## Welcome! Want to learn more? See two factoextra-related books at https://g
oo.gl/ve3WBa

## Loading required package: data.table

##
## Attaching package: 'data.table'

## The following objects are masked from 'package:dplyr':
##
##     between, first, last

## The following object is masked from 'package:purrr':
##
##     transpose

## Loading required package: ggpubr
```

```
## Loading required package: magrittr

##
## Attaching package: 'magrittr'

## The following object is masked from 'package:purrr':
##
##     set_names

## The following object is masked from 'package:tidyr':
##
##     extract

## Loading required package: corrplot

## corrplot 0.84 loaded

## Loading required package: psych

##
## Attaching package: 'psych'

## The following objects are masked from 'package:ggplot2':
##
##     %+%, alpha

## Loading required package: httr
```

Store the cat and human file paths in unique objects.

```
raw.cat.dev <- 'https://osf.io/45rjy//?action=download'
raw.cat.tsne <- 'https://osf.io/59yu6//?action=download'

raw.hum.imputed <- 'https://osf.io/6pbgr//?action=download'
raw.hum.nonimputed <- 'https://osf.io/azdv8//?action=download'
```

Import the necessary cat data CSVs from OSF. The “raw.data” object consists of 9 attribute columns (“id”, “Sample”, “Blot”, “Case”, “Age”, “Condition”, “Location”, “Tissue.Piece”, “Run.Number”) and 7 protein expression columns. The “tsne.raw.data” object consists of 8 attribute columns (“Condition”, “Labels”, “V1”, “V2”, “Cluster.Number”, “Reordered”, “Cluster.Name”, “Region”).

```
filename <- 'cat_proteins.csv'
GET(raw.cat.dev, write_disk(filename, overwrite = TRUE))

## Response [https://files.ca-1.osf.io/v1/resources/8a3kx/providers/osfstorag
e/5ed0684ec7568603012d0920?action=download&direct&version=2]
##   Date: 2020-06-09 21:11
```

```
##   Status: 200
##   Content-Type: text/csv
##   Size: 32.3 kB
## <ON DISK>  C:\Users\dezia\Dropbox (Kathy Murphy)\JB-ProteinAnalysisWorkflo
w\JB ProteinAnalysisWorkflow Markdowns\RMD files June 2020\Cat & Human Analys
is\cat_proteins.csv

raw.data <- read.csv(filename)
head(raw.data)

##     id Sample Blot       Case Age Condition Location Tissue.Piece Run.Numb
er
## 1 K252      5  A+C 2wk Normal   2         1        1            1        1
+2
## 2 K252      6  A+C 2wk Normal   2         1        1            2        1
+2
## 3 K242      3  A+C 3wk Normal   3         1        1            1        1
+2
## 4 K242      4  A+C 3wk Normal   3         1        1            2        1
+2
## 5 K244     15  B+D 4wk Normal   4         1        1            1        1
+2
## 6 K244     16  B+D 4wk Normal   4         1        1            2        1
+2
##       GluN1    GluN2A    GluN2B   GABAAa1  GABAAa3     GluA2       Syn
## 1 0.3854409 0.4268017 0.7555231 0.8563928 1.526663 0.4211669 0.5734898
## 2 0.3190519 0.4147973 0.6501530 0.6309027 1.361924 0.4979576 0.6161713
## 3 1.1909136 1.3210631 1.7467064 1.0709774 1.477091 0.7386673 0.6625438
## 4 1.2921448 1.2865798 1.6964425 1.3543666 1.851294 0.7892433 0.7016512
## 5 0.8178326 1.0010536 1.0862350 0.8661382 1.504845 0.4188479 0.8361352
## 6 0.8388763 0.6876145 0.8356130 1.9219633 2.037198 0.4474243 0.6970003

filename <- 'cat_tsne.csv'
GET(raw.cat.tsne, write_disk(filename, overwrite = TRUE))

## Response [https://files.ca-1.osf.io/v1/resources/8a3kx/providers/osfstorag
e/5ed0684f17ac9e0316621448?action=download&direct&version=1]
##   Date: 2020-06-09 21:11
##   Status: 200
##   Content-Type: text/csv
##   Size: 13.9 kB
## <ON DISK>  C:\Users\dezia\Dropbox (Kathy Murphy)\JB-ProteinAnalysisWorkflo
w\JB ProteinAnalysisWorkflow Markdowns\RMD files June 2020\Cat & Human Analys
is\cat_tsne.csv

tsne.raw.data <- read.csv(filename)
head(tsne.raw.data)

##   Condition            Labels        V1       V2 Cluster.Number Reordered
## 1    Normal 5wk Normal CVF 1 -1.421038 1.926670              1         1
## 2    Normal 5wk Normal CVF 2 -1.182249 3.150573              1         1
```

```
## 3    Normal 5wk Normal MVF 1 -1.488770 1.005343              1          1
## 4    Normal 5wk Normal MVF 2 -1.511189 1.509622              1          1
## 5    Normal 5wk Normal PVF 1 -1.438462 3.133959              1          1
## 6    Normal 5wk Normal PVF 2 -1.956975 2.357059              1          1
##   Cluster.Name Region
## 1     Normal 1    CVF
## 2     Normal 1    CVF
## 3     Normal 1    MVF
## 4     Normal 1    MVF
## 5     Normal 1    PVF
## 6     Normal 1    PVF
```

Import the necessary human data CSVs from OSF. The “raw.data.imputed” contains 1 attribute column (“ids”) and 7 protein columns. The “raw.data.nonimputed” contains 6 attribute columns (“ids”,“Age”,“AgeBin”,“Sex”,“PMI”, “PMI.Interval”) and 7 protein columns.

```
filename <- 'human_imputed.csv'
GET(raw.hum.imputed, write_disk(filename, overwrite = TRUE))
```

```
## Response [https://files.ca-1.osf.io/v1/resources/8a3kx/providers/osfstorag
e/5ece9223c7568602612d38fe?action=download&direct&version=2]
##   Date: 2020-06-09 21:11
##   Status: 200
##   Content-Type: text/csv
##   Size: 1.64 kB
## <ON DISK>  C:\Users\dezia\Dropbox (Kathy Murphy)\JB-ProteinAnalysisWorkflo
w\JB ProteinAnalysisWorkflow Markdowns\RMD files June 2020\Cat & Human Analys
is\human_imputed.csv
```

```
raw.data.imputed <- read.csv(filename)
head(raw.data.imputed)
```

```
##    ids  Gaba1  Gaba3 Synapsin  GluA2  GluN1   NR2A   NR2B
## 1  271 0.3248 1.1794   1.5397 0.7354 0.9382 2.1907 1.3247
## 2 1283 0.6098 1.7641   0.5793 1.0750 1.0786 0.8077 2.5610
## 3 1055 0.9530 1.3563   0.9279 1.1351 1.5934 1.3013 2.7864
## 4 1296 0.5215 0.9289   0.4865 0.8936 1.4743 0.4494 0.7914
## 5 1102 0.7715 1.4949   0.5107 0.8892 1.0861 1.8462 2.9910
## 6  135 0.6382 1.7209   1.2124 1.2077 1.8118 1.3394 2.5131
```

```
filename <- 'human_nonimputed.csv'
GET(raw.hum.nonimputed, write_disk(filename, overwrite = TRUE))
```

```
## Response [https://files.ca-1.osf.io/v1/resources/8a3kx/providers/osfstorag
e/5ece9223aeeb6d025a085f2b?action=download&direct&version=2]
##   Date: 2020-06-09 21:11
```

```
##   Status: 200
##   Content-Type: text/csv
##   Size: 2.34 kB
## <ON DISK>  C:\Users\dezia\Dropbox (Kathy Murphy)\JB-ProteinAnalysisWorkflo
w\JB ProteinAnalysisWorkflow Markdowns\RMD files June 2020\Cat & Human Analys
is\human_nonimputed.csv
```

```
raw.data.nonimputed <- read.csv(filename)
head(raw.data.nonimputed)
```

```
##    ids  Age  Gaba1  Gaba3 Synapsin  GluA2  GluN1   NR2A   NR2B   AgeBin Se
x PMI
## 1  271 0.05 0.3248 1.1794       NA 0.7354 0.9382     NA 1.3247     <0.3 
M   9
## 2 1157 0.05     NA     NA   0.4560     NA     NA     NA     NA     <0.3 
F  14
## 3 1283 0.24 0.6098 1.7641   0.5793 1.0750 1.0786 0.8077 2.5610     <0.3 
F  23
## 4 1055 0.26 0.9530 1.3563   0.9279 1.1351 1.5934 1.3013 2.7864     <0.3 
M  12
## 5 1296 0.27 0.5215 0.9289   0.4865 0.8936 1.4743 0.4494 0.7914     <0.3 
M  16
## 6 1102 0.33 0.7715 1.4949   0.5107 0.8892 1.0861 1.8462 2.9910     <0.3 
M  22
##   PMI.Interval
## 1          <10
## 2     10 to 15
## 3          20+
## 4     10 to 15
## 5     15 to 20
## 6          20+
```

2.) Cat Analysis

Cat: Dimension Reduction Using PCA – Additional Processing & Analysis

Rename columns in the “raw.data” object whose headers must contain special characters.

```
colnames(raw.data)[13:14] <- c('GABA\u1D00\u03b11','GABA\u1D00\u03b13')

head(raw.data)
```

```
##     id Sample Blot       Case Age Condition Location Tissue.Piece Run.Numb
er
## 1 K252      5  A+C 2wk Normal   2         1        1            1        1
+2
## 2 K252      6  A+C 2wk Normal   2         1        1            2        1
+2
## 3 K242      3  A+C 3wk Normal   3         1        1            1        1
+2
## 4 K242      4  A+C 3wk Normal   3         1        1            2        1
+2
## 5 K244     15  B+D 4wk Normal   4         1        1            1        1
+2
## 6 K244     16  B+D 4wk Normal   4         1        1            2        1
+2
##       GluN1    GluN2A    GluN2B GABA<U+1D00>a1 GABA<U+1D00>a3     GluA2
## 1 0.3854409 0.4268017 0.7555231      0.8563928       1.526663 0.4211669
## 2 0.3190519 0.4147973 0.6501530      0.6309027       1.361924 0.4979576
## 3 1.1909136 1.3210631 1.7467064      1.0709774       1.477091 0.7386673
## 4 1.2921448 1.2865798 1.6964425      1.3543666       1.851294 0.7892433
## 5 0.8178326 1.0010536 1.0862350      0.8661382       1.504845 0.4188479
## 6 0.8388763 0.6876145 0.8356130      1.9219633       2.037198 0.4474243
##         Syn
## 1 0.5734898
## 2 0.6161713
## 3 0.6625438
## 4 0.7016512
## 5 0.8361352
## 6 0.6970003
```

Assign “raw.data” to the object “my.data”. While both contain the same data, the former will be used during the subcluster analysis while the latter will be used in PCA.

```
my.data <- raw.data

head(my.data)
```

```
##     id Sample Blot       Case Age Condition Location Tissue.Piece Run.Numb
er
## 1 K252      5   A+C 2wk Normal   2         1        1            1        1
+2
## 2 K252      6   A+C 2wk Normal   2         1        1            2        1
+2
## 3 K242      3   A+C 3wk Normal   3         1        1            1        1
+2
## 4 K242      4   A+C 3wk Normal   3         1        1            2        1
+2
## 5 K244     15   B+D 4wk Normal   4         1        1            1        1
+2
## 6 K244     16   B+D 4wk Normal   4         1        1            2        1
+2
##       GluN1    GluN2A    GluN2B GABA<U+1D00>a1 GABA<U+1D00>a3     GluA2
## 1 0.3854409 0.4268017 0.7555231      0.8563928       1.526663 0.4211669
## 2 0.3190519 0.4147973 0.6501530      0.6309027       1.361924 0.4979576
## 3 1.1909136 1.3210631 1.7467064      1.0709774       1.477091 0.7386673
## 4 1.2921448 1.2865798 1.6964425      1.3543666       1.851294 0.7892433
## 5 0.8178326 1.0010536 1.0862350      0.8661382       1.504845 0.4188479
## 6 0.8388763 0.6876145 0.8356130      1.9219633       2.037198 0.4474243
##         Syn
## 1 0.5734898
## 2 0.6161713
## 3 0.6625438
## 4 0.7016512
## 5 0.8361352
## 6 0.6970003
```

Rename the “1”,“2”, and “3” factor levels in “raw.data$Location" with "CVF", "PVF", and "MVF", respectively. Assign the output in the column "raw.data$VFR”. This was done to more clearly identify where in the visual cortex an observation was taken, for the subcluster analysis.

```
raw.data$VFR <- plyr::mapvalues(raw.data$Location,
                                unique(raw.data$Location),
                                c("CVF","PVF","MVF"))
```

Combine the contents of “raw.data$Case","raw.data$VFR”, & “raw.data$Tissue.Piece", and assign them to the column "raw.data$Labels”. This was done to create unique labels for each observation.These labels were included in the prior tSNE analysis and will be used to identify which subcluster an observation falls into.

```
raw.data$Labels <- paste(raw.data$Case,
                         raw.data$VFR,
                         raw.data$Tissue.Piece)
```

Centre (but do not scale) the protein columns of “my.data”, and assign them to “my.data.scaled”.

```
my.data.scaled <- scale(my.data[,10:16],
                        center = TRUE,
                        scale = F)
```

Perform a PCA on “my.data.scaled”.

```
pca.scaled <- PCA(my.data.scaled,
                  ncp=ncol(my.data.scaled),
                  scale.unit=FALSE,
                  graph = FALSE)
```

Construct scree plot.

```
fviz_eig(pca.scaled,
         addlabels = T,
         ylim = c(0, 60),
         xlim = c(0.5,7.5),
         ncp = 7, # Select number of principal components using 'ncp' parameter
         barfill = "grey",
         barcolor = "grey",
         geom = "bar")+
  scale_y_continuous(expand = c(0,0))+
  scale_x_discrete(expand = c(0,0))+
  theme(axis.line.y=element_line(),
        axis.line.x=element_line(),
        panel.grid=element_blank())
```

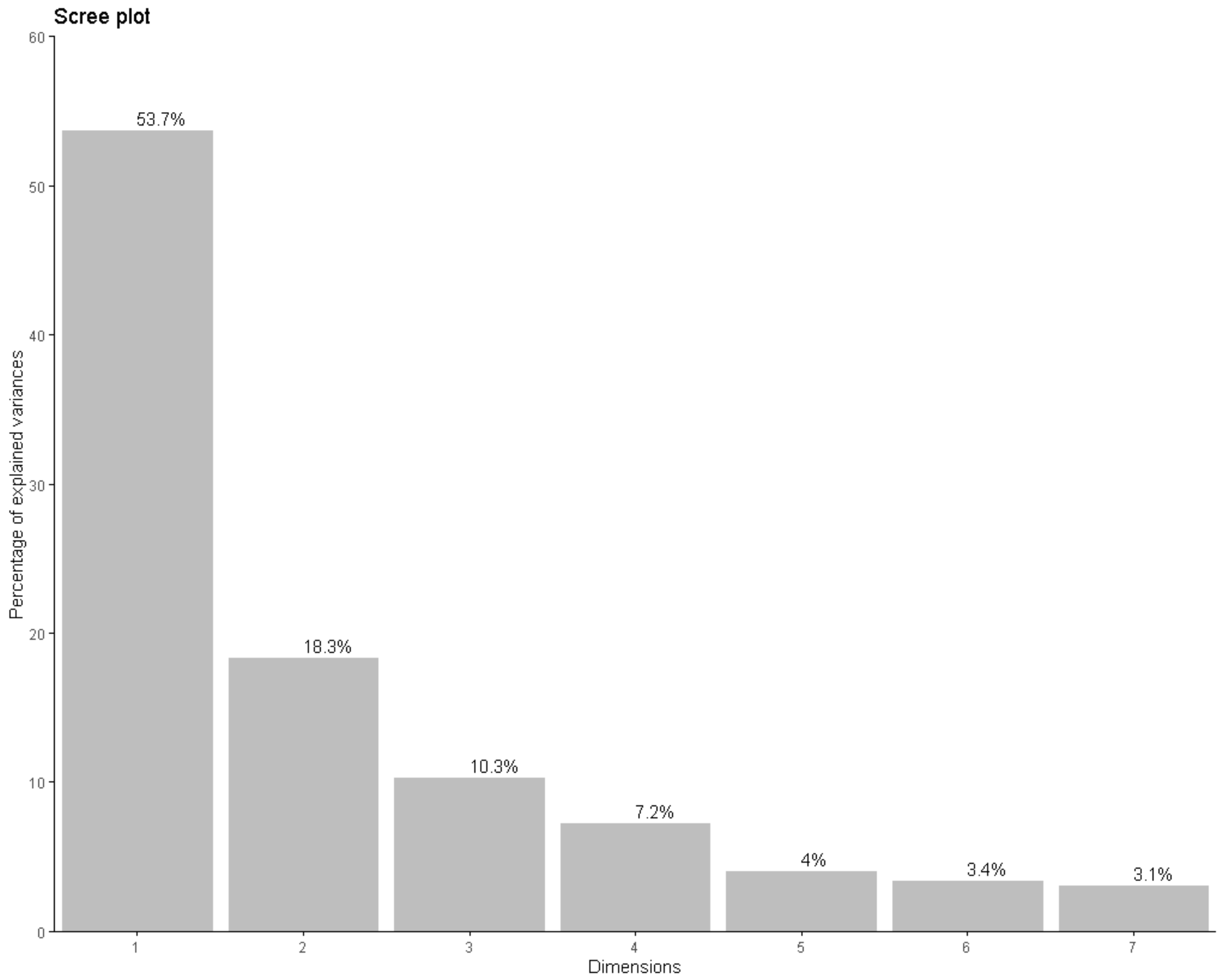

Calculate how many components are required to maintain 80% of the total variance. Store the output of this function into object, “cum.var”.

```
pca.scaled$eig[,3]

##    comp 1    comp 2    comp 3    comp 4    comp 5    comp 6    comp 7
##  53.66586  72.00511  82.27410  89.51460  93.55403  96.92361 100.00000

cum.var <- cum_var(pca.eig.3 = pca.scaled$eig[,3], # "pca.scaled$eig[,3]" is
contained within object "pca"
                   thresh = 80) # Custom threshold value

cum.var

## [1] 3
```

Cat: Identifying Candidate Plasticity Features

Construct plot of $\cos^2$ data stored in object, “pca.scaled”

```
head(pca.scaled$var$cos2)
```

```
##                    Dim.1       Dim.2       Dim.3        Dim.4       Dim.5
## GluN1        0.77881379 0.001235058 0.058361191 0.0806635837 0.020487897
## GluN2A       0.71044845 0.076807238 0.034590787 0.1288130896 0.044429485
## GluN2B       0.71924708 0.001258850 0.181659213 0.0409213505 0.024605760
## GABA<U+1D00>a1 0.08798193 0.875605314 0.002366629 0.0316236983 0.001141864
## GABA<U+1D00>a3 0.31884955 0.030507545 0.039562035 0.0177579594 0.102205636
## GluA2        0.55711246 0.025593577 0.328354478 0.0000216584 0.012722004
##                     Dim.6        Dim.7
## GluN1        0.011315701 0.0491227785
## GluN2A       0.002127357 0.0027835923
## GluN2B       0.032213898 0.0000938451
## GABA<U+1D00>a1 0.001089827 0.0001907419
## GABA<U+1D00>a3 0.419456917 0.0716603572
## GluA2        0.017388047 0.0588077725
```

```
corrplot(pca.scaled$var$cos2,is.corr=FALSE)
```

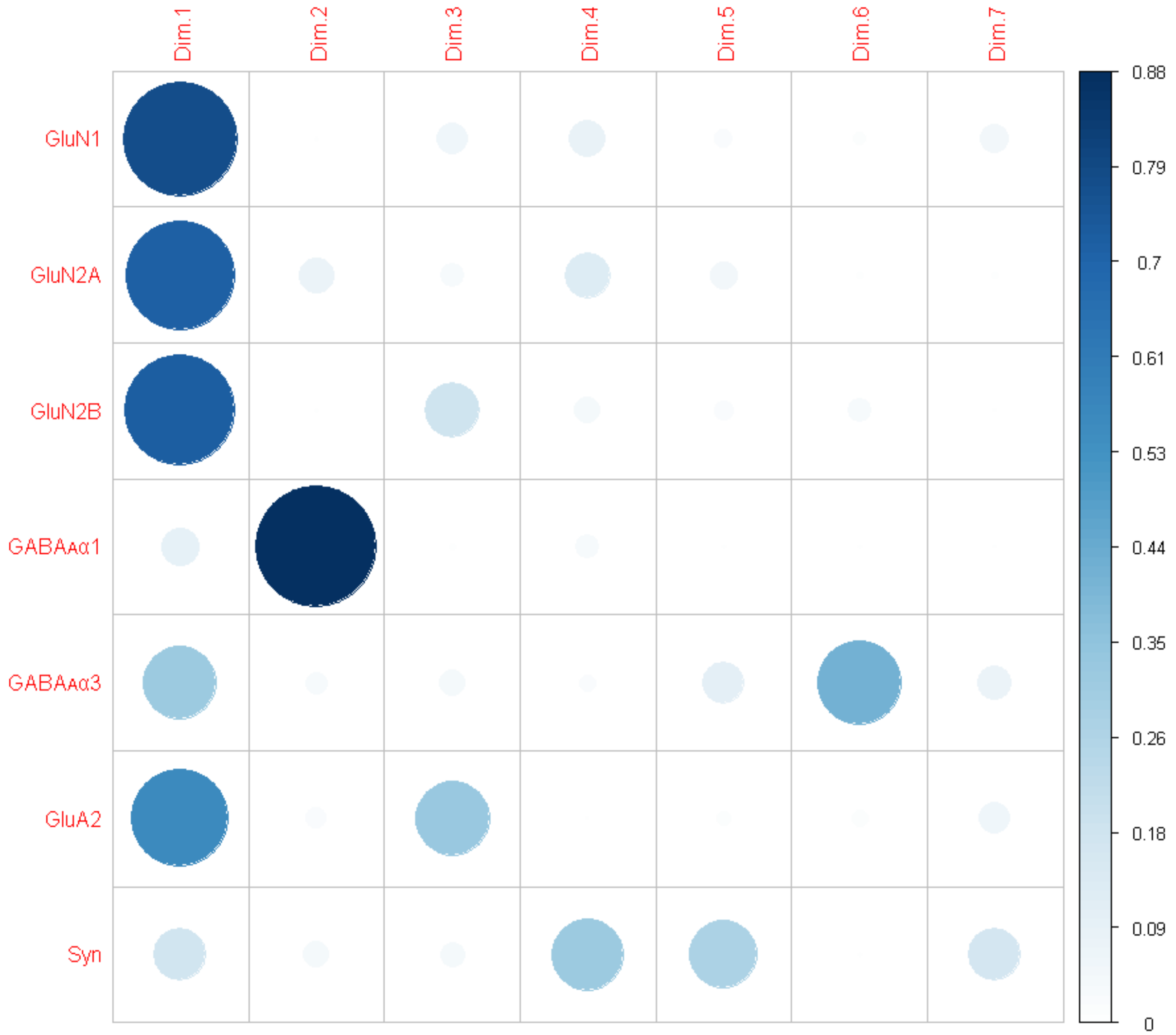

Construct plot of coord (correlation) data stored in object, "pca.scaled".

```
head(pca.scaled$var$coord)
```

```
##                     Dim.1       Dim.2       Dim.3       Dim.4       Dim.5
## GluN1           0.7372864  0.02936047  0.20182804 -0.23727839 -0.11958264
## GluN2A          0.7357148 -0.24190470 -0.16233922  0.31327319 -0.18398350
## GluN2B          0.7305037  0.03056119 -0.36712339 -0.17424420  0.13511443
## GABA<U+1D00>a1 0.2504041  0.78994861  0.04106858  0.15012429 -0.02852673
## GABA<U+1D00>a3 0.2577073  0.07971453 -0.09077645 -0.06081776  0.14590538
## GluA2           0.5521184 -0.11833855  0.42386956 -0.00344250  0.08343313
##                       Dim.6        Dim.7
## GluN1            0.08887102  0.185165851
## GluN2A           0.04025904 -0.046051740
## GluN2B          -0.15459840 -0.008344285
```

```
## GABA<U+1D00>a1 -0.02786913 -0.011659171
## GABA<U+1D00>a3  0.29558171 -0.122172473
## GluA2        -0.09754077 -0.179381694
```

```
corrplot(pca.scaled$var$coord, is.corr=F)
```

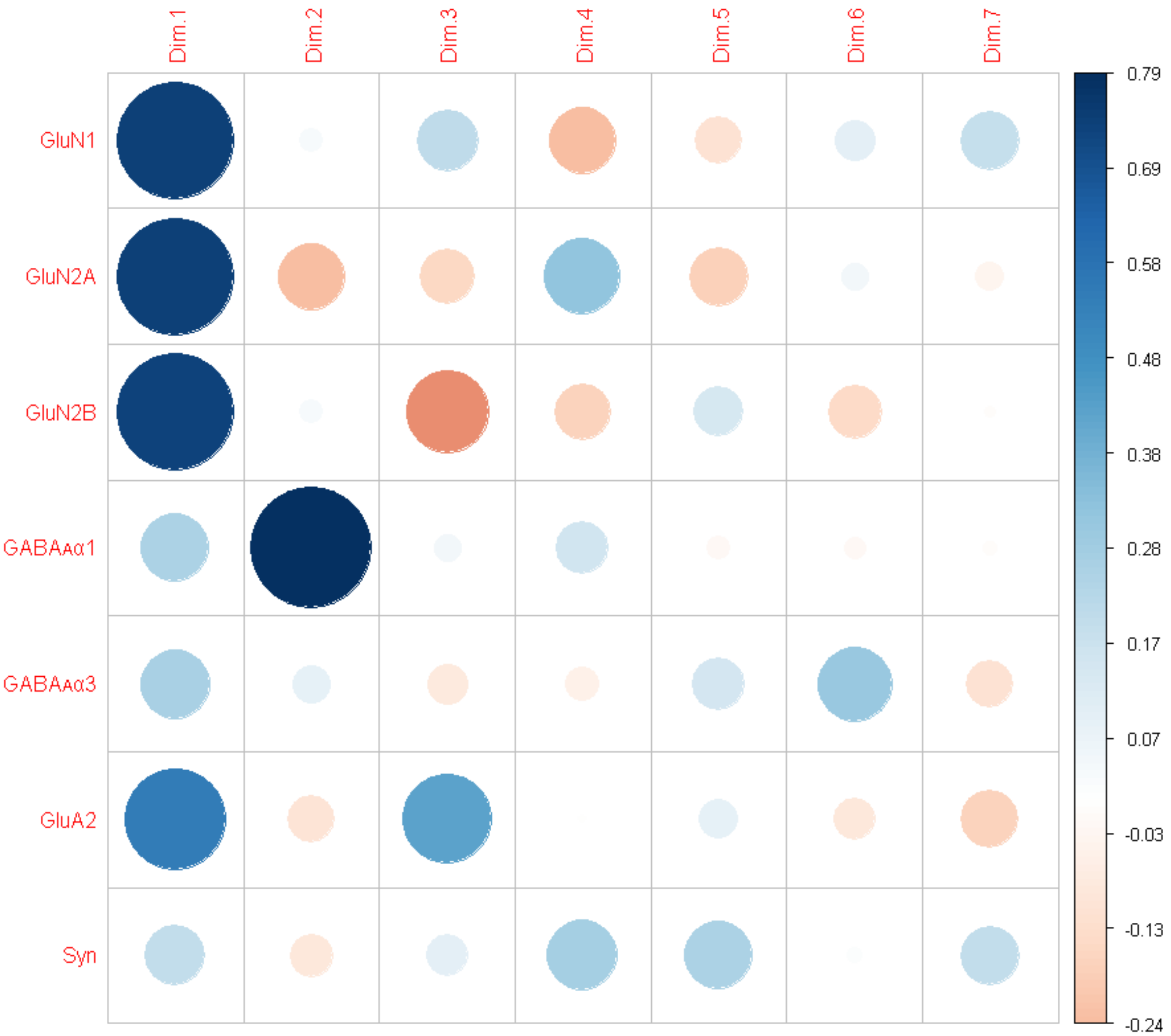

Create PCA amplitude plots for each important component. Use amplitude plots to identify relevant plasticity features.

```
pca.scaled$var$coord
```

```
##                    Dim.1      Dim.2       Dim.3       Dim.4       Dim.5
## GluN1          0.7372864  0.02936047  0.20182804 -0.23727839 -0.11958264
## GluN2A         0.7357148 -0.24190470 -0.16233922  0.31327319 -0.18398350
## GluN2B         0.7305037  0.03056119 -0.36712339 -0.17424420  0.13511443
## GABA<U+1D00>a1 0.2504041  0.78994861  0.04106858  0.15012429 -0.02852673
## GABA<U+1D00>a3 0.2577073  0.07971453 -0.09077645 -0.06081776  0.14590538
```

```
## GluA2        0.5521184 -0.11833855  0.42386956 -0.00344250  0.08343313
## Syn          0.1942073 -0.09789409  0.09216506  0.26636772  0.24871271
##                   Dim.6        Dim.7
## GluN1          0.08887102  0.185165851
## GluN2A         0.04025904 -0.046051740
## GluN2B        -0.15459840 -0.008344285
## GABA<U+1D00>a1 -0.02786913 -0.011659171
## GABA<U+1D00>a3  0.29558171 -0.122172473
## GluA2         -0.09754077 -0.179381694
## Syn            0.01262123  0.190005138
```

```
amplitude_plots(cum.var = cum.var,# Output of "cum_var()" function
                pca.var.coord = pca.scaled$var$coord) # "pca.scaled$var$coord
" is contained within object "pca"
```

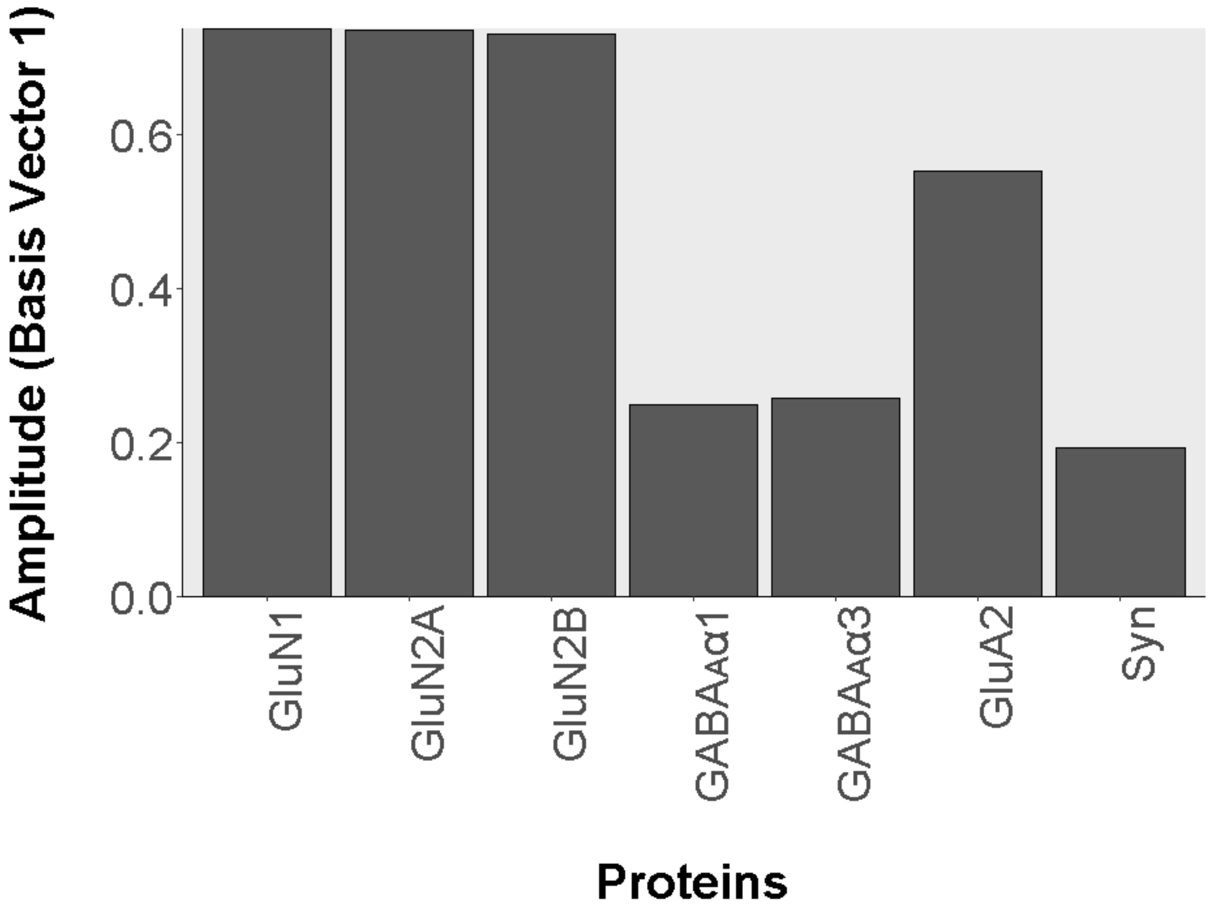
Amplitude (Basis Vector 1)
0.0
0.2
0.4
0.6
GluN1
GluN2A
GluN2B
GABAAα1
GABAAα3
GluA2
Syn
Proteins

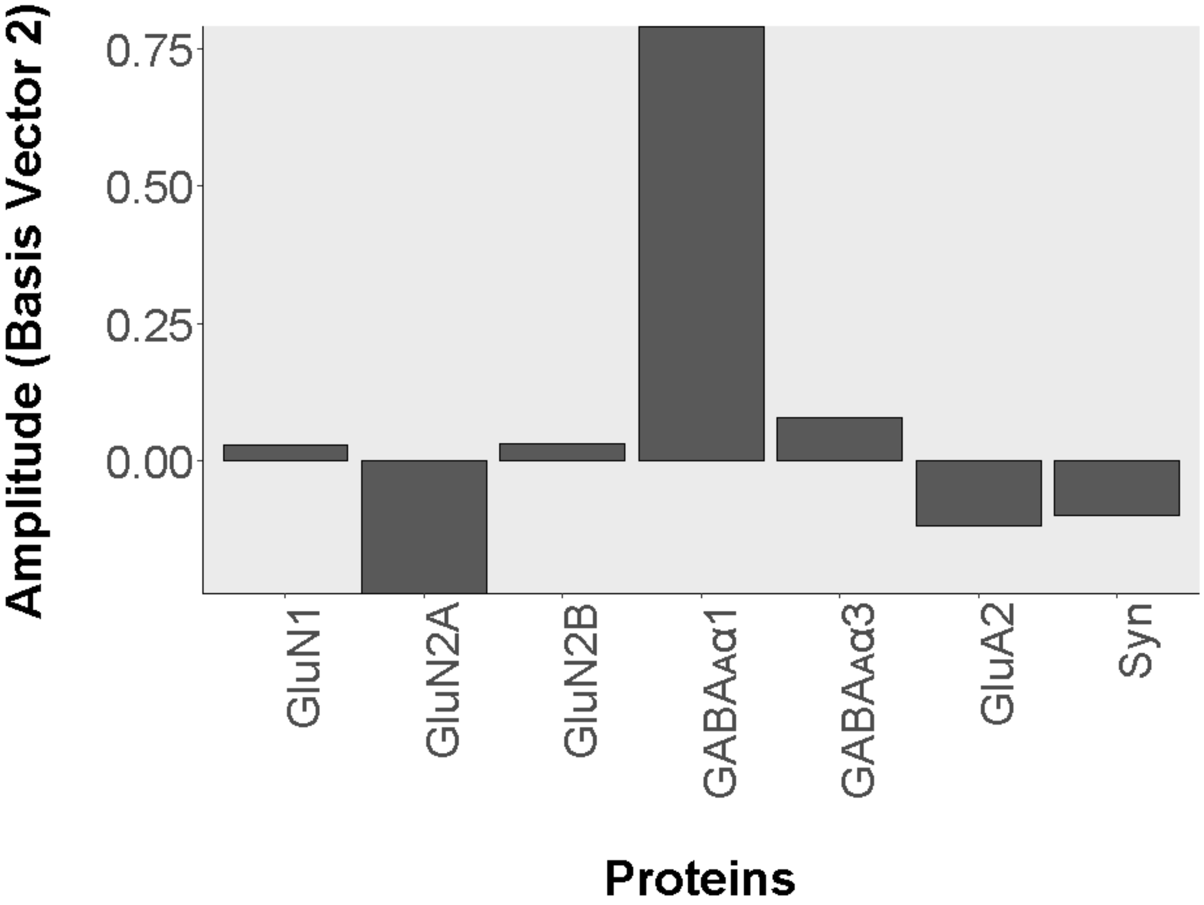
Amplitude (Basis Vector 2)
0.75
0.50
0.25
0.00
GluN1
GluN2A
GluN2B
GABAAα1
GABAAα3
GluA2
Syn
Proteins

372

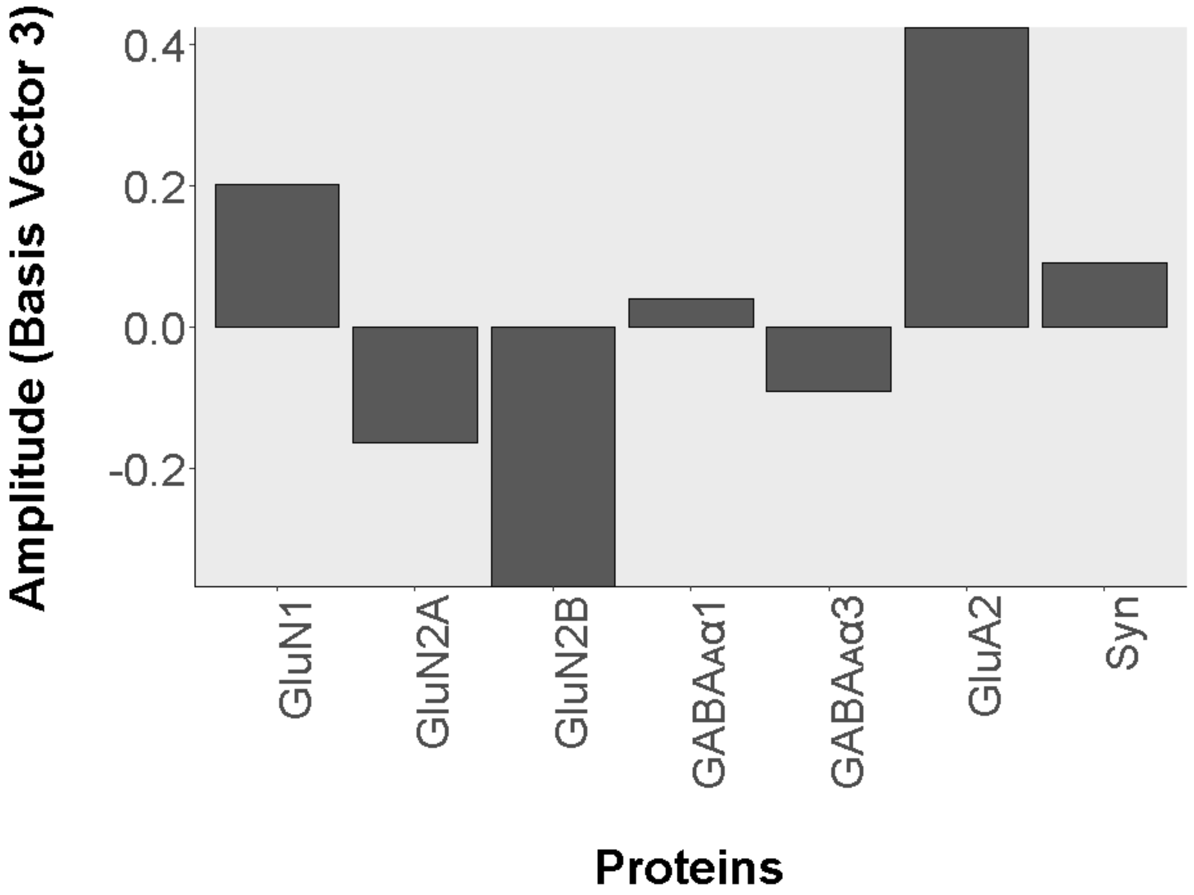

Cat: Application of the Heuristics to Identify Candidate Plasticity Features for Cat V1

Create a dataframe called "NewFeatures" to store the subsequently-calculated plasticity features.

```
NewFeatures <- data.frame(matrix(nrow = nrow(my.data),
                                 ncol = 0))
```

Calculate plasticity features.

```
NewFeatures$'Protein Sum' <- rowSums(my.data[,c('GABA\u1D00\u03b11',
                                                'GABA\u1D00\u03b13',
                                                'Syn',
                                                'GluA2',
                                                'GluN1',
                                                'GluN2A',
                                                'GluN2B')])

```

```
      prot_sums <- NewFeatures$'Protein Sum'

NewFeatures$'GlutR Sum' <- rowSums(my.data[,c('GluA2',
                                              'GluN1',
                                              'GluN2A',
                                              'GluN2B')])

      glutr_sum <- NewFeatures$'GlutR Sum'

NewFeatures$'GABA\u1D00R Sum' <- rowSums(my.data[,c('GABA\u1D00\u03b11',
                                                    'GABA\u1D00\u03b13')])

      gaba_sum <- NewFeatures$'GABA\u1D00R Sum'

      ave.glut <- NewFeatures$'GlutR Sum'/ncol(my.data[,c('GluA2',
                                                          'GluN1',
                                                          'GluN2A',
                                                          'GluN2B')])

      ave.gaba <- NewFeatures$'GABA\u1D00R Sum'/ncol(my.data[,c('GABA\u1D00\u
03b11',
                                                                'GABA\u1D00\u
03b13')])

NewFeatures$'GABA\u1D00R:GlutR' <- (ave.glut - ave.gaba)/
                              (ave.glut + ave.gaba)

NewFeatures$'GABA\u1D00\u03b11:GluN2A' <- (my.data$GluN2A - my.data$'GABA\u1D
00\u03b11')/
                                          (my.data$'GABA\u1D00\u03b11' + my.d
ata$GluN2A)

NewFeatures$'GluN2B:GluN2A'<- (my.data$GluN2A - my.data$GluN2B)/
                              (my.data$GluN2B + my.data$GluN2A)

NewFeatures$'GABA\u1D00\u03b13:GABA\u1D00\u03b11' <- (my.data$'GABA\u1D00\u03
b11' - my.data$'GABA\u1D00\u03b13')/
                                                     (my.data$'GABA\u1D00\u03
b11' + my.data$'GABA\u1D00\u03b13')

NewFeatures$'GluN2B:GluA2' <- (my.data$GluA2 - my.data$GluN2B)/
                              (my.data$GluN2B + my.data$GluA2)

NewFeatures$'GluN2A:GluA2' <- (my.data$GluA2 - my.data$GluN2A)/
                              (my.data$GluN2A + my.data$GluA2)
```

Bind the “raw.data$Labels” column to the “NewFeatures” data frame, and store the output in a new object, “NewFeatures.tsne”. The order of observations in “NewFeatures” match that of “raw.data” object. This was done so that each observation for the plasticity features had a corresponding unique subcluster label.

```
NewFeatures.tsne <- cbind(raw.data$Labels,NewFeatures)
```

Cat: Validating Candidate Plasticity Features

Reassign “pca.scaledindcord” object to “PCA.scores”.

```
PCA.scores <- pca.scaled$ind$coord
```

Perform a Bonferroni-corrected, pairwise Pearson’s correlation against PCA scores and plasticity features. Store the output in the object, “corr.scores.bf”.

```
corr.scores.bf <- corr.test(PCA.scores[,1:cum.var],
                            NewFeatures,
                            use="pairwise",
                            method="pearson",
                            adjust="bonferroni")

## Warning in abbreviate(colnames(r), minlength = minlength): abbreviate used
with
## non-ASCII chars

## Warning in abbreviate(colnames(r), minlength = minlength): abbreviate used
with
## non-ASCII chars
```

Store a matrix of correlation coefficients in the object, “corr.scores.rval”.

```
corr.scores.rval <- corr.scores.bf$r

corr.scores.rval

##        Protein Sum   GlutR Sum   GABA<U+1D00>R Sum   GABA<U+1D00>R:GlutR
## Dim.1  0.98329175  0.99125848        0.47835172           0.5350370
## Dim.2  0.13405963 -0.10803230        0.81872766          -0.6241021
## Dim.3  0.03943816  0.03461785       -0.04679652           0.1217347
##          GABA<U+1D00>a1:GluN2A GluN2B:GluN2A     GABA<U+1D00>a3:GABA<U+1D00
>a1
```

```
## Dim.1            0.43709583    0.04388945                    -0.1764722
## Dim.2           -0.74316024   -0.42092044                     0.5042847
## Dim.3           -0.07014937    0.33804970                     0.1940967
##         GluN2B:GluA2 GluN2A:GluA2
## Dim.1  -0.05781758   -0.1125934
## Dim.2  -0.20885858    0.1716322
## Dim.3   0.79782256    0.6431236
```

Store a matrix of adjusted p-values for each correlation coefficient in the object, “corr.scores.bfpval”.

```
corr.scores.bfpval <- corr.scores.bf$p

corr.scores.bfpval
```

```
##          Protein Sum     GlutR Sum   GABA<U+1D00>R Sum   GABA<U+1D00>R:Glut
R
## Dim.1 1.523827e-205 2.847155e-244      6.278714e-16        1.242786e-20
## Dim.2  6.786802e-01  1.000000e+00      2.847804e-67        4.277009e-30
## Dim.3  1.000000e+00  1.000000e+00      1.000000e+00        1.000000e+00
##          GABA<U+1D00>a1:GluN2A GluN2B:GluN2A     GABA<U+1D00>a3:GABA<U+1D00
>a1
## Dim.1           5.148524e-13  1.000000e+00                  8.369023e-02
## Dim.2           7.994234e-49  5.632186e-12                  5.681145e-18
## Dim.3           1.000000e+00  1.871047e-07                  3.023620e-02
##        GluN2B:GluA2 GluN2A:GluA2
## Dim.1 1.000000e+00 1.000000e+00
## Dim.2 1.201851e-02 1.089777e-01
## Dim.3 2.164192e-61 1.567638e-32
```

Construct plasticity feature matrix.

```
feature_matrix(
  corr.scores.pval = corr.scores.bfpval, # Matrix of adjusted p-values
  corr.scores.rval = corr.scores.rval, # Matrix of correlation coefficients
  thresh = 0.05) # Significance threshold (acceptable values range from 0 - 1
)
```

```
## Warning: Removed 12 rows containing missing values (geom_text).
```

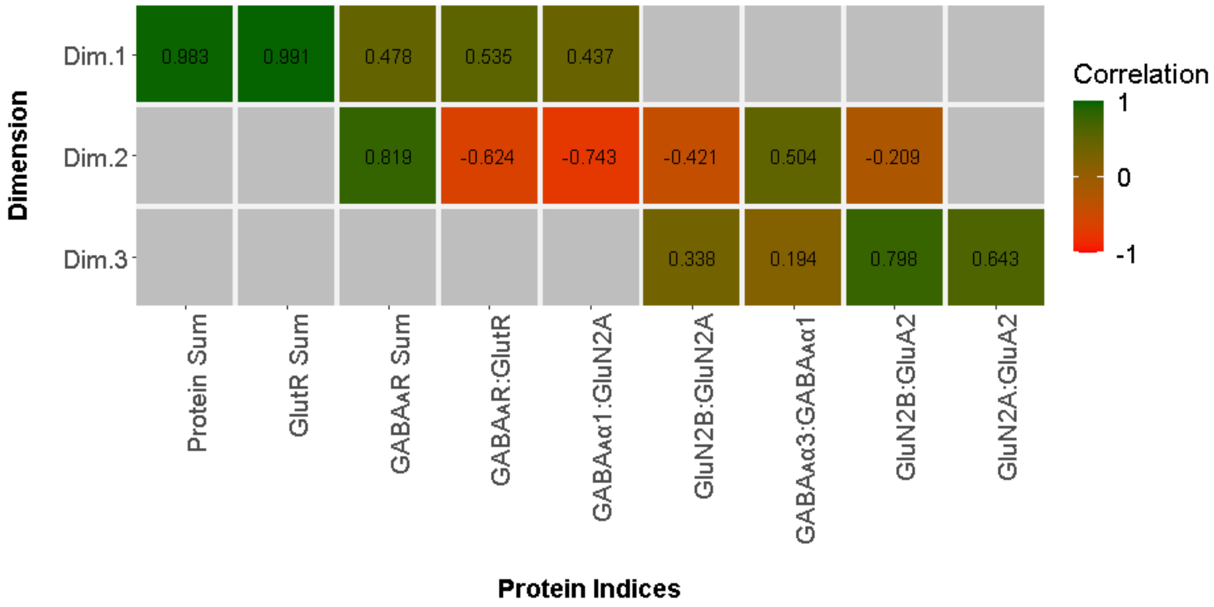

Cat: Using Plasticity Features to Construct a Plasticity Phenotype – Data Processing

Bind “NewFeatures” data frame with the nine attribute columns of “my.data”. Assign this data frame to the object, “merged.data_all”. This was done because “NewFeatures” was calculated from “my.data”, so the order of observations are maintained between the two data frames. Furthermore, the attributes are necessary to subset the cat developmental data and thus create the phenotype and boxplots.

```
merged.data_all <- cbind(my.data[,1:9],NewFeatures)
```

Subset “merged.data_all” to display only data for the normal development condition (where it is coded as a 1 in the “merged.data_all$Condition” column). Store the output in the object, “merged.data”.

```
merged.data <- subset(merged.data_all,
                      merged.data_all$Condition == 1)
```

Store the character strings of the plasticity feature columns contained within “merged.data”, to allow for easy selection of those columns in subsequent analysis. Store these strings in the object, “NewFeatCol”.

```
NewFeatCol <- colnames(merged.data)[10:ncol(merged.data)]
```

Calculate the median of each plasticity feature in “merged.features[,NewFeatsCol]” across all levels of “list(merged.features$Case)”. Store the output in object, “cat.dev”.

```
cat.dev <- aggregate(merged.data[,NewFeatCol],
                   list(merged.data$Case),
                   median)
```

Reorder the rows of “cat.dev” based on the specified order of the “cat.dev$Group.1” levels. Reassign this data to “cat.dev”.

```
cat.dev <-  cat.dev[match(c("2wk Normal",
                            "3wk Normal",
                            "4wk Normal",
                            "5wk Normal",
                            "6wk Normal",
                            "8wk Normal",
                            "12wk Normal",
                            "16wk Normal",
                            "24wk Normal"),
                    cat.dev$Group.1),]
```

Rename the levels of “cat.dev$Group.1" and reassign those names to "cat.dev$Group.1”. This is done to customize the appearance of the age groups that appear on the cat developmental phenotype.

```
cat.dev$Group.1 <- plyr::mapvalues(cat.dev$Group.1,
                             unique(cat.dev$Group.1),
                             c("2wk",
                               "3wk",
                               "4wk",
                               "5wk",
                               "6wk",
                               "8wk",
                               "12wk",
                               "16wk",
                               "Adult"))
```

Assign the the contents of the “Group.1” column as row names for the “cat.dev” data frame.

```
rownames(cat.dev) <- cat.dev[,'Group.1']
```

3.) Human: Analysis

Human: Translational Research Using Plasticity Phenotypes

Merge “raw.data.imputed” and “raw.data.nonimputed” by shared “ids” column. Store the output in the object, “raw.data.allproteins”.

```
raw.data.allproteins <- merge(raw.data.imputed,
                              raw.data.nonimputed[,c("ids",
                                                     "Age",
                                                     "AgeBin",
                                                     "Sex")],
                              by.x = "ids",
                              by.y = "ids")

head(raw.data.allproteins)
```

```
##   ids  Gaba1  Gaba3 Synapsin  GluA2  GluN1   NR2A   NR2B  Age   AgeBin Sex
## 1   3 1.0395 1.5813   3.8900 1.8893 1.0565 5.1471 4.8389 5.39  5 to 11   M
## 2 103 1.3457 1.2796   1.7013 1.4039 1.1076 3.0127 1.9369 2.21   1 to 4   F
## 3 135 0.6382 1.7209   1.2124 1.2077 1.8118 1.3394 2.5131 0.33    <0.3    M
## 4 166 0.4611 0.8364   0.7692 1.2135 1.9642 0.3896 0.8482 0.37 0.3 to 1   F
## 5 271 0.3248 1.1794   1.5397 0.7354 0.9382 2.1907 1.3247 0.05     <0.3   M
## 6 451 0.8640 1.3357   0.9809 1.3880 0.9515 2.3311 3.0684 4.56   1 to 4   M
```

Reorder “raw.data.allproteins” so that all relevant attribute columns precede the protein columns. Store this reordered data frame in the object, “syn.prots”.

```
syn.prots     <- raw.data.allproteins [,c("ids",
                                          "Age",
                                          "AgeBin",
                                          "Gaba1",
                                          "Gaba3",
                                          "Synapsin",
                                          "GluA2",
                                          "GluN1",
                                          "NR2A",
                                          "NR2B")]
```

Assign “syn.prots” to the new object, “my.data” but convert the “syn.prots$ids” column to row names.

```
my.data <- column_to_rownames(syn.prots, var = "ids")
```

Rename protein data columns of “my.data” that contain special characters.

```
colnames(my.data) <- c('Age',
                       'AgeBin',
                       "GABA\u1D00\u03b11",
                       'GABA\u1D00\u03b13',
                       "Syn",
                       "GluA2",
                       "GluN1",
                       "GluN2A",
                       "GluN2B")

head(my.data)
```

```
##       Age   AgeBin GABA<U+1D00>a1 GABA<U+1D00>a3    Syn  GluA2  GluN1 GluN2
A
## 3   5.39  5 to 11         1.0395         1.5813 3.8900 1.8893 1.0565 5.147
1
## 103 2.21   1 to 4         1.3457         1.2796 1.7013 1.4039 1.1076 3.012
7
## 135 0.33     <0.3         0.6382         1.7209 1.2124 1.2077 1.8118 1.339
4
## 166 0.37 0.3 to 1         0.4611         0.8364 0.7692 1.2135 1.9642 0.389
6
## 271 0.05     <0.3         0.3248         1.1794 1.5397 0.7354 0.9382 2.190
7
## 451 4.56   1 to 4         0.8640         1.3357 0.9809 1.3880 0.9515 2.331
1
##     GluN2B
## 3   4.8389
## 103 1.9369
## 135 2.5131
## 166 0.8482
## 271 1.3247
## 451 3.0684
```

Human: Application of the Heuristics to Identify Candidate Plasticity Features

Create a data frame called “NewFeatures” to store the plasticity features.

```
NewFeatures <- data.frame(matrix(nrow = nrow(my.data),
                                 ncol = 0))
```

Calculate plasticity features determined in section 2 (Cat Analysis).

```
NewFeatures$'Protein Sum' <-
  rowSums(my.data[,c('GABA\u1D00\u03b11',
                     'GABA\u1D00\u03b13',
                     'Syn',
                     'GluA2',
                     'GluN1',
                     'GluN2A',
                     'GluN2B')])

prot_sums <-
  NewFeatures$'Protein Sum'

NewFeatures$'GlutR Sum' <-
  rowSums(my.data[,c('GluA2',
                     'GluN1',
                     'GluN2A',
                     'GluN2B')])

glutr_sum <- NewFeatures$'GlutR Sum'

NewFeatures$'GABA\u1D00R Sum' <-
  rowSums(my.data[,c('GABA\u1D00\u03b11',
                     'GABA\u1D00\u03b13')])

gaba_sum <-
  NewFeatures$'GABA\u1D00R Sum'

ave.glut <-
  NewFeatures$'GlutR Sum'/
  ncol(my.data[,c('GluA2',
                  'GluN1',
                  'GluN2A',
                  'GluN2B')])

ave.gaba <-
  NewFeatures$'GABA\u1D00R Sum'/
  ncol(my.data[,c('GABA\u1D00\u03b11',
                  'GABA\u1D00\u03b13')])

NewFeatures$'GABA\u1D00R:GlutR' <-
  (ave.glut - ave.gaba)/
  (ave.glut + ave.gaba)

NewFeatures$'GABA\u1D00\u03b11:GluN2A' <-
  (my.data$GluN2A - my.data$'GABA\u1D00\u03b11')/
  (my.data$'GABA\u1D00\u03b11' + my.data$GluN2A)
```

```r
NewFeatures$'GluN2B:GluN2A'<-
  (my.data$GluN2A - my.data$GluN2B)/
  (my.data$GluN2B + my.data$GluN2A)

NewFeatures$'GABA\u1D00\u03b13:GABA\u1D00\u03b11' <-
  (my.data$'GABA\u1D00\u03b11' - my.data$'GABA\u1D00\u03b13')/
  (my.data$'GABA\u1D00\u03b11' + my.data$'GABA\u1D00\u03b13')

NewFeatures$'GluN2B:GluA2' <-
  (my.data$GluA2 - my.data$GluN2B)/
  (my.data$GluN2B + my.data$GluA2)

NewFeatures$'GluN2A:GluA2' <-
  (my.data$GluA2 - my.data$GluN2A)/
  (my.data$GluN2A + my.data$GluA2)
```

Human: Using Plasticity Features to Construct a Plasticity Phenotype – Data Processing

Bind the “Age” and “AgeBin” columns of “my.data” with the “NewFeatures” data frame. Store this new data frame into the object, “merged.features”.

```r
merged.features <- cbind(data.frame(my.data[c('Age', 'AgeBin')]), NewFeatures
)
```

Assign the column names for the plasticity features within “merged.features” to the new object, “NewFeatsCol”. This will allow for the subsetting of the plasticity feature data to be easier.

```r
NewFeatsCol <- colnames(merged.features)[3:ncol(merged.features)]
```

Calculate the median of each plasticity feature in “merged.features[,NewFeatsCol]” across all levels of “list(merged.features$AgeBin)”. Store content in the object, “hum.dev”.

```r
hum.dev <- aggregate(merged.features[,NewFeatsCol],
                     list(merged.features$AgeBin),
                     median)
```

Assign and order the factor levels of the “hum.dev$Group.1”. Arrange the factors in ascending order from “<0.3” to “55+”. This was done to specify the order of age-bins along the X-axis of the phenotype.

```
hum.dev$Group.1 <- factor(hum.dev$Group.1,
                          levels = c('<0.3',
                                     '0.3 to 1',
                                     '1 to 4',
                                     '5 to 11',
                                     '12 to 20',
                                     '21 to 55',
                                     '55+'),
                          ordered = TRUE)
```

Reorder “hum.dev” rows according to the previously assigned factor order of “hum.dev$Group.1”.

```
hum.dev <- hum.dev[match(c("<0.3",
                           "0.3 to 1",
                           "1 to 4",
                           "5 to 11" ,
                           "12 to 20",
                           "21 to 55",
                           "55+"),
                         hum.dev$Group.1),]
```

Assign the the contents of the “Group.1” column as row names for the “hum.dev” data frame.

```
rownames(hum.dev) <- hum.dev[,'Group.1']
```

4.) Create Cat & Human Phenotypes

Cat & Human: Using Plasticity Features to Construct a Plasticity Phenotype – Creating Phenotypes

Combine the plasticity feature columns in the human and cat data frames into a list, and store this list in the object, “df_list”.

```
df_list <- list(hum.dev[,2:ncol(hum.dev)],
                cat.dev[,2:ncol(cat.dev)])
```

Construct a plasticity phenotype from the human and cat developmental data

```
plasticity_phenotype(df_list = df_list,
                     first_index_column = 4,
                     group_label = c('\nAge Bins (Years)',
                                     '\nAge (Weeks)'),
                     translation = 'absolute')
```

```
##                                <0.3 0.3 to 1  1 to 4 5 to 11 12 to 20 21 to
55
## Protein Sum              #4C4C4C  #6F6F6F #3F3F3F #444444  #4F4F4F  #0000
00
## GlutR Sum                #1B1B1B  #323232 #1F1F1F #2F2F2F  #545454  #0000
00
## GABA<U+1D00>R Sum              #1F1F1F  #959595 #2C2C2C #393939  #292929  #00
0000
## GABA<U+1D00>R:GlutR            #D0FF00  #ADFF00 #CBFF00 #D8FF00  #EAFF00  #DD
FF00
## GABA<U+1D00>a1:GluN2A          #BEFF00  #E9FF00 #8EFF00 #B7FF00  #C3FF00  #7A
FF00
## GluN2B:GluN2A            #FFA800  #FF8F00 #FFF400 #FFD100  #EBFF00  #98FF
00
## GABA<U+1D00>a3:GABA<U+1D00>a1 #FF8800  #FFB100 #FFE300 #FFDB00  #F8FF00  #
FFE200
## GluN2B:GluA2             #FF8B00  #FF2900 #FFB500 #FF9300  #FFF700  #FF9A
00
## GluN2A:GluA2             #FFEE00  #ECFF00 #FFA300 #FFC100  #FFD600  #FF00
00
##                               55+
## Protein Sum              #868686
## GlutR Sum                #888888
## GABA<U+1D00>R Sum              #070707
## GABA<U+1D00>R:GlutR            #FFD600
## GABA<U+1D00>a1:GluN2A          #FF9300
```

```
## GluN2B:GluN2A               #FFD500
## GABA<U+1D00>a3:GABA<U+1D00>a1 #FFFE00
## GluN2B:GluA2                #FFD400
## GluN2A:GluA2                #DDFF00
##                                 2wk     3wk     4wk     5wk     6wk     8wk
## Protein Sum                 #EDEDED #C0C0C0 #C7C7C7 #969696 #AEAEAE #000000
## GlutR Sum                   #F6F6F6 #CECECE #DCDCDC #B0B0B0 #C3C3C3 #000000
## GABA<U+1D00>R Sum             #D1D1D1 #2A2A2A #1C1C1C #363636 #252525 #00000
0
## GABA<U+1D00>R:GlutR           #FF7800 #FFE200 #FFAB00 #EEFF00 #FFFC00 #9FFF0
0
## GABA<U+1D00>a1:GluN2A         #FF8100 #F9FF00 #FF8C00 #E3FF00 #FBFF00 #D4FF0
0
## GluN2B:GluN2A               #FFAC00 #FFC500 #FFE300 #FDFF00 #F7FF00 #FFFF00
## GABA<U+1D00>a3:GABA<U+1D00>a1 #FFD100 #FFC700 #E6FF00 #FFF100 #E6FF00 #F0F
F00
## GluN2B:GluA2                #FFBF00 #FF6F00 #FF7500 #FFE400 #FF9200 #FFDB00
## GluN2A:GluA2                #EDFF00 #FFAA00 #FFAC00 #FFE400 #FF9100 #EBFF00
##                                12wk    16wk   Adult
## Protein Sum                 #414141 #D5D5D5 #D8D8D8
## GlutR Sum                   #444444 #DADADA #E0E0E0
## GABA<U+1D00>R Sum             #747474 #CFCFCF #CBCBCB
## GABA<U+1D00>R:GlutR           #94FF00 #C4FF00 #F6FF00
## GABA<U+1D00>a1:GluN2A         #8FFF00 #C6FF00 #FFF400
## GluN2B:GluN2A               #DDFF00 #ADFF00 #9DFF00
## GABA<U+1D00>a3:GABA<U+1D00>a1 #EDFF00 #64FF00 #64FF00
## GluN2B:GluA2                #EEFF00 #E8FF00 #AEFF00
## GluN2A:GluA2                #FFF000 #FFBA00 #FFE200

## [[1]]
```

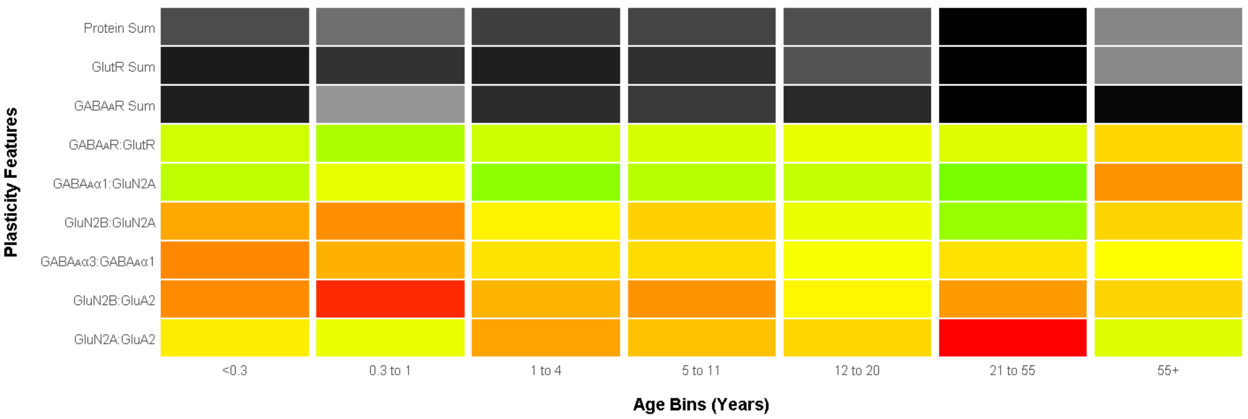


```
##
## [[2]]
```

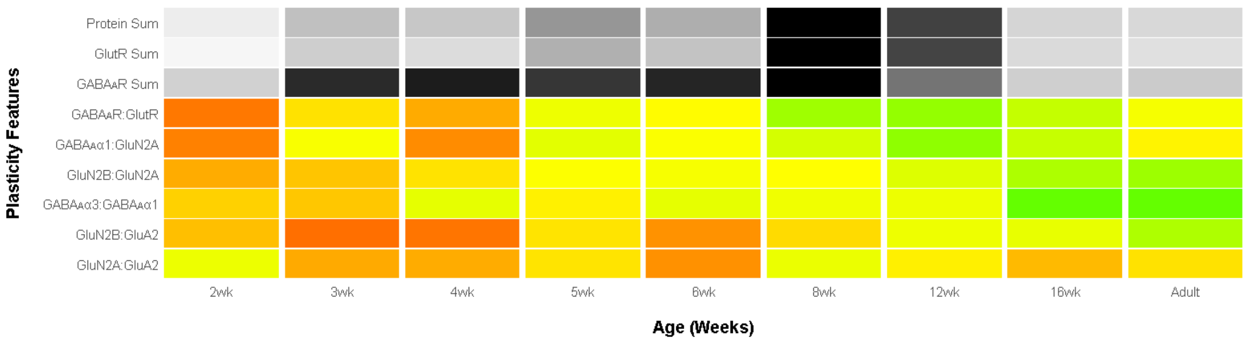

Store the human and cat colour-codes in their respective "hum.cols" and "cat.cols" objects.

```
hum.cols <- phen.cols[[1]]
cat.cols <- phen.cols[[2]]
```

5.) Create Cat Boxplots

Cat: Using Plasticity Features to Construct a Plasticity Phenotype – Creating Boxplots

Assign a subset of columns – specifically, the "Case" column, and the column headers for each plasticity feature stored within "NewFeatCol") – from "merged.data" that are necessary to create the cat developmental phenotype boxplots. Store these data in the object, "feats_df".

```
feats_df <- as.data.frame(merged.data[,c("Case",NewFeatCol)])
```

Rename levels of 'feats_df$Case' and reassign them to "feats_df$Case". This is done to customize the appearance of the age groups that appear on the cat developmental boxplots.

```
feats_df$Case <- plyr::mapvalues(feats_df$Case,
                           unique(feats_df$Case),
                           c("2wk",
                             "3wk",
                             "4wk",
                             "5wk",
                             "6wk",
                             "8wk",
                             "12wk",
                             "16wk",
                             "24wk"))
```

Assign an order to the levels of "feats_df$Case", and then reassign to "feats_df$Case". The age groups will appear in ascending order, from "2wk" to "24wk".

```
feats_df$Case <- factor(feats_df$Case,
                        levels = c( "2wk",
                                    "3wk",
                                    "4wk",
                                    "5wk",
                                    "6wk",
                                    "8wk",
                                    "12wk",
                                    "16wk",
```

```
                                               "24wk"),
                                    ordered = TRUE)
```

Divide all data points in a “feats_df” sum by the median value of that corresponding sum for the “2wk” age group in the object, “cat.dev”. Store these normalized data into own “sum” object.

```
sum1 <- feats_df[,'Protein Sum']/
         cat.dev[cat.dev$Group.1 == "2wk",
                 'Protein Sum']

sum2 <- feats_df[,'GlutR Sum']/
         cat.dev[cat.dev$Group.1 == "2wk",
                 'GlutR Sum']

sum3 <- feats_df[,'GABA\u1D00R Sum']/
        cat.dev[cat.dev$Group.1 == "2wk",
                'GABA\u1D00R Sum']
```

Reassign the objects “sum1”,“sum2”, & “sum3” to their corresponding sums column in “feats_df”.

```
feats_df[,'Protein Sum'] <- sum1
feats_df[,'GlutR Sum'] <- sum2
feats_df[,'GABA\u1D00R Sum'] <- sum3
```

Assign “feats_df” to “feats_df2”. This is done to ensure the contents of “feats_df” aren’t mistakenly overwritten.

```
feats_df2 <- feats_df
```

Append each column name for the sums in “feats_df” with “\n (Normalized to 2wk)”. Store the output in the corresponding columns of “feats_df2”.

```
colnames(feats_df2)[2:4] <- paste0(colnames(feats_df)[2:4],
                                   "\n (Normalized to 2wk)")
```

Append each column name for the indices in “feats_df” with “\n”. Store the output in the corresponding columns of “feats_df2”.

```
colnames(feats_df2)[5:ncol(feats_df2)] <- paste0(colnames(feats_df)[5:ncol(fe
ats_df2)],
                                                 "\n")
```

Create individual boxplots colour-coded according to the cat developmental phenotype.

```
head(feats_df2)
```

```
##   Case Protein Sum\n (Normalized to 2wk) GlutR Sum\n (Normalized to 2wk)
## 1  2wk                          2.146473                        2.519942
## 2  2wk                          1.949199                        2.384410
## 3  3wk                          3.562481                        6.331554
## 4  3wk                          3.893973                        6.416518
## 5  4wk                          2.834671                        4.211410
## 6  4wk                          3.240314                        3.559622
##   GABA<U+1D00>R Sum\n (Normalized to 2wk) GABA<U+1D00>R:GlutR\n
## 1                           2.175087          -0.411126707
## 2                           1.818913          -0.358526315
## 3                           2.325699          -0.009787201
## 4                           2.925903          -0.117370427
## 5                           2.164067          -0.175800645
## 6                           3.613645          -0.476217884
##   GABA<U+1D00>a1:GluN2A\n GluN2B:GluN2A\n GABA<U+1D00>a3:GABA<U+1D00>a1\n
## 1             -0.33478252     -0.27802972                       -0.28126504
## 2             -0.20666095     -0.22100155                       -0.36682639
## 3              0.10454911     -0.13874683                       -0.15938105
## 4             -0.02566762     -0.13739844                       -0.15501567
## 5              0.07225582     -0.04080959                       -0.26938471
## 6             -0.47300707     -0.09716108                       -0.02910578
##   GluN2B:GluA2\n GluN2A:GluA2\n
## 1     -0.2841498    -0.00664508
## 2     -0.1325616     0.09110914
## 3     -0.4055886    -0.28275343
## 4     -0.3649694    -0.23958523
## 5     -0.4434222    -0.41003249
## 6     -0.3025545    -0.21161415
```

```
phenotype_boxplots(feature_df = feats_df2 , # Boxplot data frame
                   phenotype_cols = cat.cols, # Phenotype colour-code data frame
                   first_index_column = 4, # Index number of first index column in "feats_df2" (indexes begin at 0)
                   group_label = "\nAge (Weeks)", # X-axis label
                   point_size = 0.6, # Desired size of geom_jitter points
                   point_alpha = 1, # Desired transparency of geom_jitter points
                   aspect_ratio = 5/7, # Desired aspect ratio of each boxplot
                   text_size = 8) # Desired X- & Y-axis text size for each boxplot
```

Create a 3 x 3 matrix that displays all of the colour-coded boxplots on a single figure.

```
ggarrange(plotlist = plot_list,
          nrow = 3,
          ncol = 3,
          labels = LETTERS[1:9],
          font.label = list(size = 10),
          vjust = 1)
```

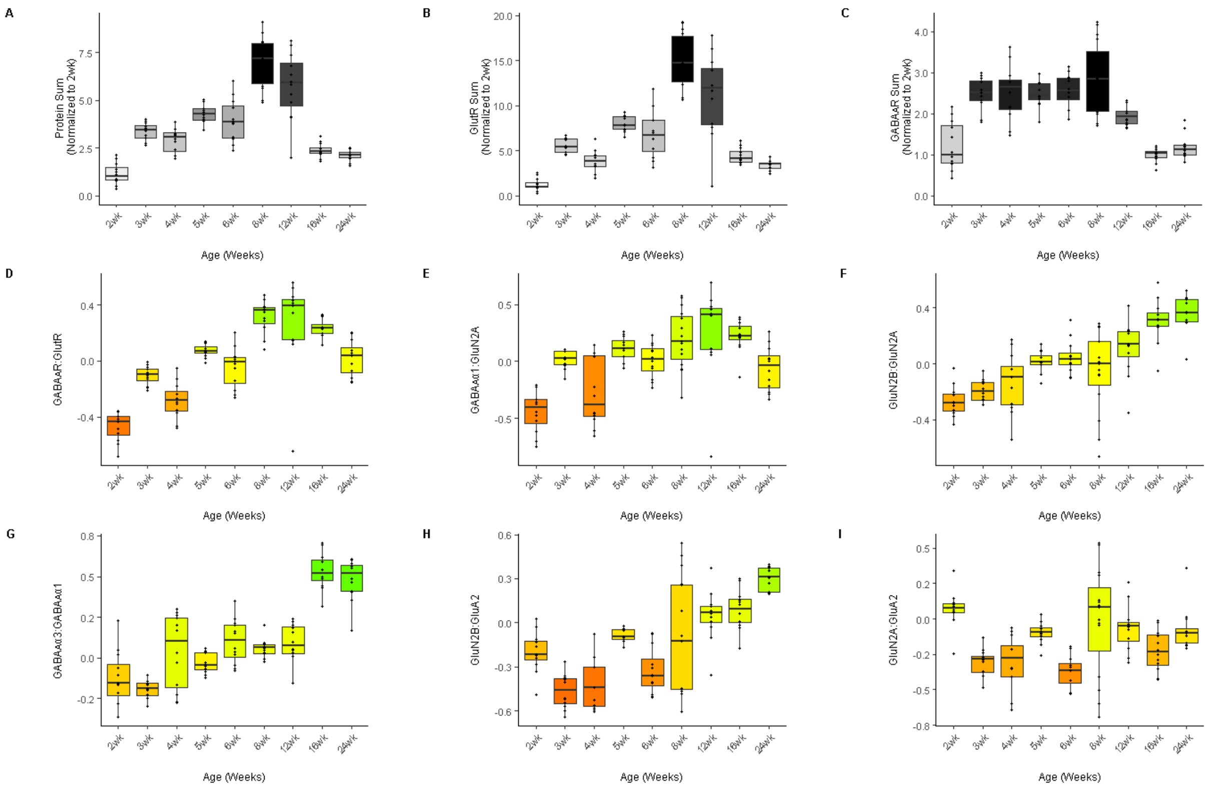

6.) Create Human Boxplots

Human: Using Plasticity Features to Construct a Plasticity Phenotype – Creating Boxplots

Assign all but the first column of “merged.features” (merged.features$Age“) to”feats_df“.

```
feats_df <- merged.features[,-1]
```

Assign an order to the levels of “feats_df$AgeBin", and then reassign to "feats_df$AgeBin”. Arrange the factors in ascending order from “<0.3” to “55+”. This was done to specify the order of the age-bins along the X-axis of the boxplots.

```
feats_df$AgeBin <- factor(feats_df$AgeBin,
                          levels = c('<0.3',
                                     '0.3 to 1',
                                     '1 to 4',
                                     '5 to 11',
                                     '12 to 20',
                                     '21 to 55',
                                     '55+'),
                          ordered = TRUE)
```

Assign “feats_df” to “feats_df2”.

```
feats_df2 <- feats_df
```

Divide all data points in a “feats_df” sum by the median value of that corresponding sum for the “<0.3” age-bin in the “hum.dev” object. Store these normalized data in their own “sum” objects.

```
sum1 <- feats_df[,'Protein Sum']/
  unlist(hum.dev[hum.dev$Group.1 =='<0.3',
                 'Protein Sum'])

sum2 <- feats_df[,'GlutR Sum']/
  unlist(hum.dev[hum.dev$Group.1 =='<0.3',
                 'GlutR Sum'])

sum3 <-  feats_df[,'GABA\u1D00R Sum']/
```

```r
  unlist(hum.dev[hum.dev$Group.1 =='<0.3',
                 'GABA\u1D00R Sum'])
```

Reassign objects “sum1”,“sum2”, & “sum3” to their respective sums column in “feats_df2”.

```r
feats_df2[,'Protein Sum'] <- sum1
feats_df2[,'GlutR Sum'] <- sum2
feats_df2[,'GABA\u1D00R Sum'] <- sum3
```

Assign “feats_df2” to “feats_df3”.

```r
feats_df3 <- feats_df2
```

Append each column name for the sums in “feats_df2” with “\n (Normalized to <0.3)”. Store the output in the corresponding columns of “feats_df3”.

```r
colnames(feats_df3)[2:4] <- paste0(colnames(feats_df2)[2:4],
                                   "\n (Normalized to <0.3)")
```

Append each column name for the indices in “feats_df2” with “\n”. Store the output in the corresponding columns of “feats_df3”.

```r
colnames(feats_df3)[5:ncol(feats_df3)] <- paste0(colnames(feats_df2)[5:ncol(f
eats_df3)],"\n")
```

Create individual boxplots that are colour-coded according to the human developmental phenotype.

```r
head(feats_df3)

##       AgeBin Protein Sum\n (Normalized to <0.3)
## 3    5 to 11                          2.1525040
## 103   1 to 4                          1.3050246
## 135     <0.3                          1.1562073
## 166 0.3 to 1                          0.7176489
## 271     <0.3                          0.9114702
## 451   1 to 4                          1.2089166
##     GlutR Sum\n (Normalized to <0.3) GABA<U+1D00>R Sum\n (Normalized to <0
.3)
## 3                         2.0967993                                1.1455
296
## 103                       1.2097642                                1.1474
966
## 135                       1.1142459                                1.0311
```

```
428
## 166                    0.7159419                                      0.5671
263
## 271                    0.8413594                                      0.6574
732
## 451                    1.2548238                                      0.9614
704
##      GABA<U+1D00>R:GlutR\n GABA<U+1D00>a1:GluN2A\n GluN2B:GluN2A\n
## 3                0.4231569              0.66395112      0.03086321
## 103              0.1738949              0.38247981      0.21735090
## 135              0.1858294              0.35457120     -0.30465931
## 166              0.2596819             -0.08404843     -0.37049604
## 271              0.2660112              0.74176108      0.24634465
## 451              0.2751269              0.45917186     -0.13654968
##      GABA<U+1D00>a3:GABA<U+1D00>a1\n GluN2B:GluA2\n GluN2A:GluA2\n
## 3                        -0.20673077     -0.4383936    -0.46299244
## 103                       0.02517807     -0.1595426    -0.36426210
## 135                      -0.45894621     -0.3508385    -0.05170586
## 166                      -0.28924855      0.1771839     0.51394174
## 271                      -0.56814253     -0.2860541    -0.49735142
## 451                      -0.21443833     -0.3770757    -0.25358286
```

```
phenotype_boxplots(feature_df = feats_df3, # Boxplot data frame
                   phenotype_cols = hum.cols, # Phenotype colour-code data fr
ame
                   first_index_column = 4, # Index number of first index colu
mn in "feats_df4" (indexes begin at 0)
                   group_label = "\nAge Bins (Years)", # X-axis label
                   point_size = 0.7, # Desired size of geom_jitter points
                   point_alpha = 1, # Desired transparency of geom_jitter poi
nts
                   aspect_ratio = 5/7) # Desired aspect ratio of each boxplot
```

Create a 3 x 3 matrix displaying all colour-coded boxplots on a single figure.

```
ggarrange(plotlist = plot_list,
          nrow = 3,
          ncol = 3,
          labels = LETTERS[1:9],
          font.label = list(size = 12),
          vjust = 1)
```

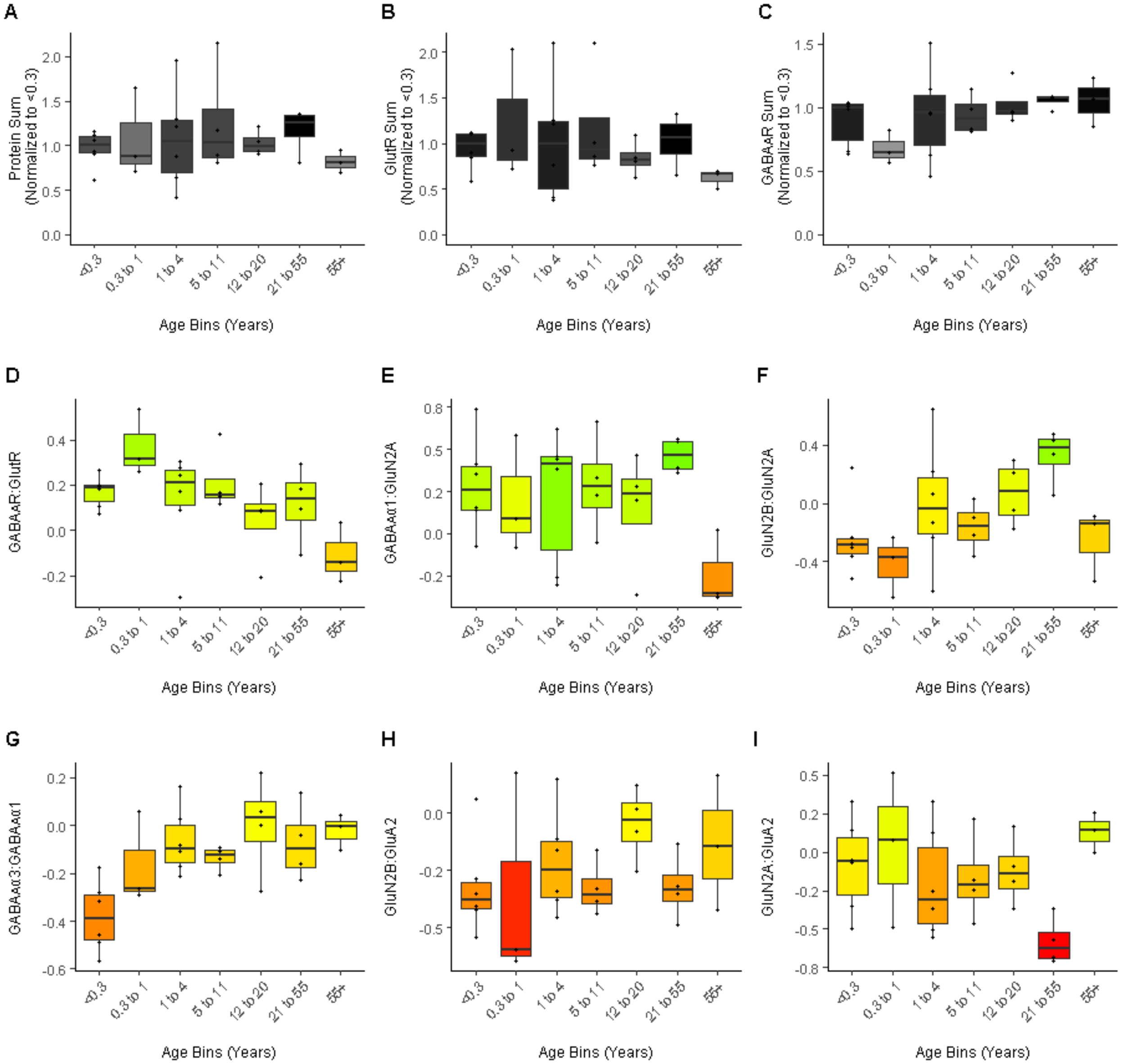
A
Protein Sum
(Normalized to <0.3)
Age Bins (Years)
B
GlutR Sum
(Normalized to <0.3)
Age Bins (Years)
C
GABAAR Sum
(Normalized to <0.3)
Age Bins (Years)
D
GABAAR:GlutR
Age Bins (Years)
E
GABAAα1:GluN2A
Age Bins (Years)
F
GluN2B:GluN2A
Age Bins (Years)
G
GABAAα3:GABAAα1
Age Bins (Years)
H
GluN2B:GluA2
Age Bins (Years)
I
GluN2A:GluA2
Age Bins (Years)
<0.3
0.3 to 1
1 to 4
5 to 11
12 to 20
21 to 55
55+

7.) Cat tSNE Analysis

Cat: Using Plasticity Phenotypes for Clustering of Experience-Dependent Changes in V1 – Data Processing

Rename the first column of “NewFeature.tsne” to “Labels”.

```
colnames(NewFeatures.tsne)[1] <- 'Labels'
```

Merge “NewFeatures.tsne” and “tsne.raw.data[,c(‘Labels’,‘Cluster.Name’)]” by their mutual “Labels” column, and store the output in “tsne.processed.data”. This was done to map the unique subcluster labels onto the corresponding observation, so that the subcluster each observation fell into (stored in “tsne.raw.data$Cluster.Name”) could be determined.

```
tsne.processed.data <- merge(NewFeatures.tsne,
                             tsne.raw.data[,c('Labels',
                                              'Cluster.Name')],
                             by.x = 'Labels',
                             by.y = 'Labels')
```

Assign the “tsne.processed.data” data frame to the object, “tsne.processed.data2”, except the “Labels” column in “tsne.processed.data” which becomes the row names of “tsne.processed.data2”.

```
tsne.processed.data2 <- column_to_rownames(tsne.processed.data, "Labels")
```

Reorder “tsne.processed.data2” columns and store the output in the object, “tsne.processed.data3”. The first column is which subcluster each observation falls into (“Cluster.Name”), followed by the sums and indices calculated for each observation.

```
tsne.processed.data3 <- tsne.processed.data2[,c(colnames(tsne.processed.data2
)[10],
                                                colnames(tsne.processed.data2
)[1:9])]
```

Count how many instances of each subcluster level are within the "tsne.processed.data3" and store the output in the object, "enough.counts".

```
enough.counts <- plyr::count(tsne.processed.data3$Cluster.Name)
```

Assign the labels of select subclusters (determined according to Balsor et al. 2019) to the object, "clustered.groups".

```
clustered.groups <- c("Normal 1",
                      "LT BV 1",
                      "MD 1",
                      "LT BV 5",
                      "LT BV 4",
                      "RO 2",
                      "ST BV 3",
                      "MD 3",
                      "ST BV 5",
                      "ST BV 1",
                      "LT BV 6",
                      "BD 3",
                      "BD 6")
```

Subset 'enough.counts' to retain subcluster labels in the "enough.counts$x" column that are also contained in the object, "clustered.groups".

```
enough.counts <- subset(enough.counts,
                        enough.counts$x %in% clustered.groups)
```

Rename the select subcluster labels stored within "tsne.processed.data3$Cluster.Name" to include the visual cortex location of each sample, and store the output in the new column, "tsne.processed.data3$Cluster.Name2".

```
tsne.processed.data3$Cluster.Name2 <- plyr::mapvalues(tsne.processed.data3$Cl
uster.Name,
                                                      unique(tsne.processed.data3$Cluster
.Name),
                                                      c( 'RO 2\n C,P,M' ,
                                                        'LT BV 1\n C,P,M' ,
                                                        'LT BV 4\n P,M' ,
                                                        'LT BV 5\n P,M' ,
                                                        'ST BV 3\n C,P,M' ,
                                                        'ST BV 1\n C,P,M' ,
```

```
                                            'ST BV 5\n P' ,
                                            'ST BV 6' ,
                                            'ST BV 4' ,
                                            'LT BV 6\n P' ,
                                            'BD 3\n C,P,M' ,
                                            'BD 5',
                                            'BD 6\n P',
                                            'LT BV 3' ,
                                            'MD 3\n C,P' ,
                                            'MD 1\n P,M' ,
                                            'MD 4' ,
                                            'Normal 1\n C,P,M'))
```

Assign a factor order to the levels of "tsne.processed.data3$Cluster.Name2". This ordering was done according to (Balsor et al., 2019) and was necessary to properly order the subcluster levels when displayed on the phenotype.

```
tsne.processed.data3$Cluster.Name2 <- factor(
                                      tsne.processed.data3$Cluster.Name2,
                                      levels = c('Normal 1\n C,P,M',
                                                 'LT BV 1\n C,P,M',
                                                 'MD 1\n P,M',
                                                 'LT BV 5\n P,M',
                                                 'LT BV 4\n P,M',
                                                 'RO 2\n C,P,M',
                                                 'ST BV 3\n C,P,M',
                                                 'MD 3\n C,P',
                                                 'ST BV 5\n P',
                                                 'ST BV 1\n C,P,M',
                                                 'LT BV 6\n P',
                                                 'BD 3\n C,P,M',
                                                 'BD 6\n P'),
                                      ordered = TRUE)
```

Assign a factor order to the levels of "tsne.processed.data3$Cluster.Name" columns. This ordering was done according to (Balsor et al., 2019) and was necessary to properly order the subcluster levels when displayed on the phenotype boxplots.

```
tsne.processed.data3$Cluster.Name <- factor(
                                     tsne.processed.data3$Cluster.Name,
                                     levels = c('Normal 1',
                                                'LT BV 1',
```

```
                                                'MD 1',
                                                'LT BV 5',
                                                'LT BV 4',
                                                'RO 2',
                                                'ST BV 3',
                                                'MD 3',
                                                'ST BV 5',
                                                'ST BV 1',
                                                'LT BV 6',
                                                'BD 3',
                                                'BD 6'),
                                      ordered = TRUE)
```

Subset the “tsne.processed.data3” data frame based on items in “tsne.processed.data3$Cluster.Name" that are also present in the "enough.counts$x” column. Store the output in the object, “tsne.processed.data4”.

```
tsne.processed.4 <- subset(tsne.processed.data3,
                           tsne.processed.data3$Cluster.Name %in% enough.coun
ts$x)
```

Calculate the median values of each plasticity feature across all levels of “tsne.processed.data4$Cluster.Name2”. Store the output in the object, “cat.subcl_0.5”.

```
cat.subcl_0.5 <- aggregate(tsne.processed.4[,NewFeatCol],
                           list(tsne.processed.4$Cluster.Name2),
                           median)
```

Store “cat.subcl_0.5” in “cat.subcl”. This was done to avoid potentially overwriting the contents of “cat.subcl_0.5”.

```
cat.subcl <- cat.subcl_0.5
```

Reverse the signs of “cat.subcl_0.5$'GABA\u1D00\u03b13:GABA\u1D00\u03b11" and store the output in "cat.subcl$’GABAD003b13:GABAD003b11”.

```
cat.subcl$'GABA\u1D00\u03b13:GABA\u1D00\u03b11' <- cat.subcl_0.5$'GABA\u1D00\
u03b13:GABA\u1D00\u03b11'*-1
```

Rename the 8th column of the “cat.subcl”.

```r
colnames(cat.subcl)[8] <- 'GABA\u1D00\u03b11:GABA\u1D00\u03b13'
```

Cat: Constructing and Visualizing the Plasticity Phenotypes for the Subclusters – Create Phenotype

Assign the contents of the “Group.1” column as row names for the “cat.subcl” data frame.

```r
rownames(cat.subcl) <- cat.subcl$Group.1
```

Create a plasticity phenotype using the “cat.subcl” data.

```r
head(cat.subcl)
```

```
##                               Group.1 Protein Sum GlutR Sum GABA<U+1D00>R Sum
## Normal 1\n C,P,M Normal 1\n C,P,M   10.054874  6.240234          2.664731
## LT BV 1\n C,P,M   LT BV 1\n C,P,M    8.983626  5.654809          2.008049
## MD 1\n P,M             MD 1\n P,M    9.672284  6.299899          2.644214
## LT BV 5\n P,M       LT BV 5\n P,M    6.088109  3.781250          1.228423
## LT BV 4\n P,M       LT BV 4\n P,M    8.208065  5.690774          1.635767
## RO 2\n C,P,M         RO 2\n C,P,M    8.391937  4.463141          1.982746
##                  GABA<U+1D00>R:GlutR GABA<U+1D00>a1:GluN2A GluN2B:GluN2A
## Normal 1\n C,P,M          0.06713220            0.11370947   0.009268495
## LT BV 1\n C,P,M           0.17430524            0.01721096  -0.164778667
## MD 1\n P,M                0.08728357           -0.02049818  -0.308250943
## LT BV 5\n P,M             0.20686317            0.27958360   0.023554669
## LT BV 4\n P,M             0.27555796            0.14812194  -0.222851584
## RO 2\n C,P,M              0.08007062            0.04127925   0.286154842
##                  GABA<U+1D00>a1:GABA<U+1D00>a3 GluN2B:GluA2 GluN2A:GluA2
## Normal 1\n C,P,M                    0.04220642  -0.09389056  -0.08669236
## LT BV 1\n C,P,M                    -0.20718232  -0.25534466  -0.14229348
## MD 1\n P,M                         -0.17115154  -0.32646982  -0.02025748
## LT BV 5\n P,M                       0.14884904  -0.14511606  -0.05281835
## LT BV 4\n P,M                      -0.08800405  -0.36991967  -0.19746244
## RO 2\n C,P,M                       -0.07850049   0.42592757   0.15801685
```

```r
plasticity_phenotype(df_list = list(cat.subcl[,-1]), # Median values data fra
me
                     first_index_column = 4, # Index number of first index co
lumn in "cat.subcl" (indexes begin at 0)
                     group_label = "\nSubclusters", # X-axis labels
                     translation = 'local') # Desired colour-scale
```

```
##                           Normal 1\n C,P,M LT BV 1\n C,P,M MD 1\n P,M
## Protein Sum                        #252525         #464646    #303030
## GlutR Sum                          #090909         #292929    #040404
## GABA<U+1D00>R Sum                    #BEBEBE         #CECECE    #BFBFBF
```

```
## GABA<U+1D00>R:GlutR                  #B5FF00        #7DFF00    #ACFF00
## GABA<U+1D00>a1:GluN2A                #86FF00        #AAFF00    #B7FF00
## GluN2B:GluN2A                      #E8FF00        #FFB800    #FF5900
## GABA<U+1D00>a1:GABA<U+1D00>a3          #7DFF00        #ECFF00    #DEFF00
## GluN2B:GluA2                       #FFC700        #FF7500    #FF4400
## GluN2A:GluA2                       #FF9E00        #FF6900    #FFD500
##                           LT BV 5\n P,M LT BV 4\n P,M RO 2\n C,P,M
## Protein Sum                      #AAAAAA       #5F5F5F      #595959
## GlutR Sum                        #969696       #272727      #6C6C6C
## GABA<U+1D00>R Sum                  #E1E1E1       #D7D7D7      #CFCFCF
## GABA<U+1D00>R:GlutR                #66FF00       #00FF00      #AFFF00
## GABA<U+1D00>a1:GluN2A              #00FF00       #77FF00      #A2FF00
## GluN2B:GluN2A                    #E1FF00       #FF9500      #00FF00
## GABA<U+1D00>a1:GABA<U+1D00>a3        #00FF00       #BDFF00      #B8FF00
## GluN2B:GluA2                     #FFAE00       #FF0000      #00FF00
## GluN2A:GluA2                     #FFBB00       #FF0000      #A5FF00
##                           ST BV 3\n C,P,M MD 3\n C,P ST BV 5\n P
## Protein Sum                        #090909    #000000     #A0A0A0
## GlutR Sum                          #181818    #000000     #9F9F9F
## GABA<U+1D00>R Sum                    #6B6B6B    #747474     #D8D8D8
## GABA<U+1D00>R:GlutR                  #FDFF00    #FAFF00     #B4FF00
## GABA<U+1D00>a1:GluN2A                #FFD900    #FFE700     #B9FF00
## GluN2B:GluN2A                      #FF9400    #FF0000     #FF9500
## GABA<U+1D00>a1:GABA<U+1D00>a3          #FFB300    #FFD600     #9DFF00
## GluN2B:GluA2                       #FF9B00    #FF9F00     #FF5D00
## GluN2A:GluA2                       #E3FF00    #B1FF00     #FFA200
##                           ST BV 1\n C,P,M LT BV 6\n P BD 3\n C,P,M BD 6\n P
## Protein Sum                        #585858     #B1B1B1      #202020  #9C9C9C
## GlutR Sum                          #454545     #B5B5B5      #7E7E7E  #D2D2D2
## GABA<U+1D00>R Sum                    #C7C7C7     #D2D2D2      #000000  #BBBBBB
## GABA<U+1D00>R:GlutR                  #C3FF00     #E8FF00      #FF4900  #FF0000
## GABA<U+1D00>a1:GluN2A                #C6FF00     #F4FF00      #FF2800  #FF0000
## GluN2B:GluN2A                      #FFA600     #DAFF00      #FF4400  #FF8000
## GABA<U+1D00>a1:GABA<U+1D00>a3          #F9FF00     #FF6700      #FF0000  #FF8000
## GluN2B:GluA2                       #FF2400     #FFB000      #FFEA00  #FF8600
## GluN2A:GluA2                       #FF4D00     #FF3900      #00FF00  #A1FF00

## [[1]]
```

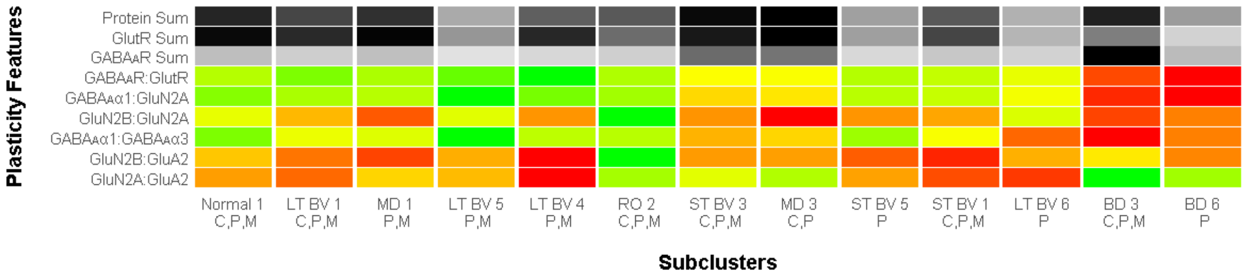

Assign the tSNE phenotype colour-code to the object “tsne.cols”.

```
tsne.cols <- phen.cols[[1]]
```

Rename the 8th column of the “tsne.processed.4”.

```
colnames(tsne.processed.4)[8] <- 'GABA\u1D00\u03b11:GABA\u1D00\u03b13'
```

Store the “tsne.processed.4” object into the new object, “tsne.processed.5”. This was done to avoid potentially overwriting the contents of “tsne.processed.4”.

```
tsne.processed.5 <- tsne.processed.4
```

Append a subset of the column headers of “tsne.processed.5” with an “\n”.

```
colnames(tsne.processed.5)[2:10] <- paste0(colnames(tsne.processed.4)[c(2:10)
],"\n")
```

Cat: Constructing and Visualizing the Plasticity Phenotypes for the Subclusters – Create Boxplots

Create individual colour-coded boxplots for cat subcluster data.

```
head(tsne.processed.5[,-ncol(tsne.processed.5)])

##                 Cluster.Name Protein Sum\n GlutR Sum\n GABA<U+1D00>R Sum\n
## 18d RO CVF 1            RO 2      9.326704    5.307563            2.142932
## 18d RO CVF 2            RO 2     10.556702    5.316230            2.694749
## 18d RO MVF 1            RO 2      9.658435    5.497712            2.100966
## 18d RO MVF 2            RO 2      8.259729    4.771556            1.727323
## 18d RO PVF 1            RO 2      9.216756    4.490773            2.830239
## 18d RO PVF 10           RO 2      8.754209    5.089096            1.522142
##                 GABA<U+1D00>R:GlutR\n GABA<U+1D00>a1:GluN2A\n GluN2B:GluN2A\
```

```
n
## 18d RO CVF 1              0.10649975              0.11897191       0.280133
5
## 18d RO CVF 2             -0.00684382             -0.02566512       0.303105
7
## 18d RO MVF 1              0.13359050              0.06702033       0.292176
2
## 18d RO MVF 2              0.16008720              0.09124979       0.333386
8
## 18d RO PVF 1             -0.11522762             -0.17862038       0.257593
6
## 18d RO PVF 10             0.25140999              0.29200612       0.367567
0
##               GABA<U+1D00>a1:GABA<U+1D00>a3\n GluN2B:GluA2\n GluN2A:GluA2\
n
## 18d RO CVF 1                        0.02462795      0.3838222      0.116180
6
## 18d RO CVF 2                        0.05244266      0.4242947      0.139074
9
## 18d RO MVF 1                        0.06432353      0.5487068      0.305509
6
## 18d RO MVF 2                        0.16977656      0.5336853      0.243649
5
## 18d RO PVF 1                        0.10214623      0.4249325      0.187907
2
## 18d RO PVF 10                       0.03174592      0.4604927      0.111859
2
```

```
phenotype_boxplots(feature_df = tsne.processed.5[,-ncol(tsne.processed.5)], #
Boxplot data frame
                   phenotype_cols = tsne.cols, # Phenotype colour-code data f
rame
                   first_index_column = 4, # Index number of first index colu
mn in "tsne.processed.5[,-ncol(tsne.processed.5)]" (indexes begin at 0)
                   group_label = "\nSubclusters", # X-axis label
                   point_size = 0.7, # Desired size of geom_jitter points
                   point_alpha = 0.5, # Desired transparency of geom_jitter p
oints
                   aspect_ratio = 9/10) # Desired aspect ratio of each boxplo
t
```

Create a single figure displaying all colour-coded boxplots for the subcluster phenotypes.

```
ggarrange(plotlist = plot_list,
          nrow = 3,
          ncol = 3,
          labels = LETTERS[1:9],
          font.label = list(size = 12))
```

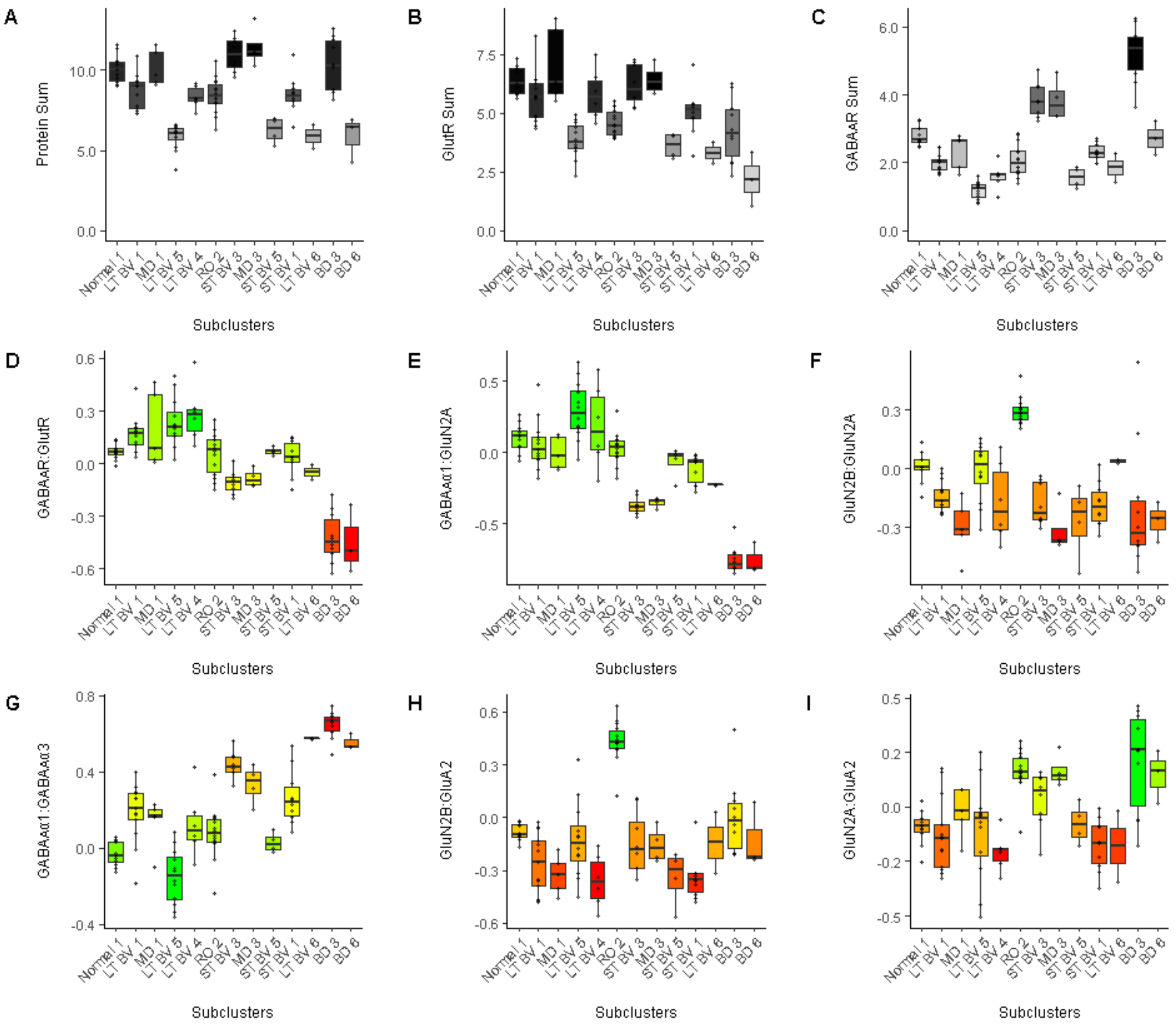

Combine contents of "tsne.processed.4$Cluster.Name2" and numbers from 1 to the total number of rows in the "tsne.processed.4" data frame. Assign these combined identifiers as the row names of the "tsne.processed.4" object.

```
rownames(tsne.processed.4) <- paste(tsne.processed.4$Cluster.Name2,1:nrow(tsne.processed.4))
```

Cat: Interpretation of the plasticity phenotype and cluster analysis for classifying experience-dependent changes in V1

Create an ORA_phenotype using the object, “tsne.processed.4[,2:10]” with default percentile values: "c(0.25,0.75)".

```
head(tsne.processed.4[,2:10])
```

```
##                Protein Sum GlutR Sum GABA<U+1D00>R Sum GABA<U+1D00>R:GlutR
## RO 2\n C,P,M 1    9.326704  5.307563          2.142932          0.10649975
## RO 2\n C,P,M 2   10.556702  5.316230          2.694749         -0.00684382
## RO 2\n C,P,M 3    9.658435  5.497712          2.100966          0.13359050
## RO 2\n C,P,M 4    8.259729  4.771556          1.727323          0.16008720
## RO 2\n C,P,M 5    9.216756  4.490773          2.830239         -0.11522762
## RO 2\n C,P,M 6    8.754209  5.089096          1.522142          0.25140999
##                GABA<U+1D00>a1:GluN2A GluN2B:GluN2A
## RO 2\n C,P,M 1            0.11897191     0.2801335
## RO 2\n C,P,M 2           -0.02566512     0.3031057
## RO 2\n C,P,M 3            0.06702033     0.2921762
## RO 2\n C,P,M 4            0.09124979     0.3333868
## RO 2\n C,P,M 5           -0.17862038     0.2575936
## RO 2\n C,P,M 6            0.29200612     0.3675670
##                GABA<U+1D00>a1:GABA<U+1D00>a3 GluN2B:GluA2 GluN2A:GluA2
## RO 2\n C,P,M 1                    0.02462795    0.3838222    0.1161806
## RO 2\n C,P,M 2                    0.05244266    0.4242947    0.1390749
## RO 2\n C,P,M 3                    0.06432353    0.5487068    0.3055096
## RO 2\n C,P,M 4                    0.16977656    0.5336853    0.2436495
## RO 2\n C,P,M 5                    0.10214623    0.4249325    0.1879072
## RO 2\n C,P,M 6                    0.03174592    0.4604927    0.1118592
```

```
ORA_phenotype(
  features_df_row  = tsne.processed.4[,2:10], # Data frame for bootstrap anal
ysis
  condition_list = as.list(c('Normal 1\n C,P,M',
                      'LT BV 1\n C,P,M',
                      'MD 1\n P,M',
                      'LT BV 5\n P,M',
                      'LT BV 4\n P,M',
                      'RO 2\n C,P,M',
                      'ST BV 3\n C,P,M',
                      'MD 3\n C,P',
                      'ST BV 5\n P',
                      'ST BV 1\n C,P,M',
                      'LT BV 6\n P',
                      'BD 3\n C,P,M',
                      'BD 6\n P')), # List of subclusters as they appear in ro
w names of "features_df_row"
  reference_group =  'Normal 1\n C,P,M', # Name of reference group as it appe
```

```
ars in the row names of "features_df_row"
  group_label  = "\nSubclusters") # X-axis label
```

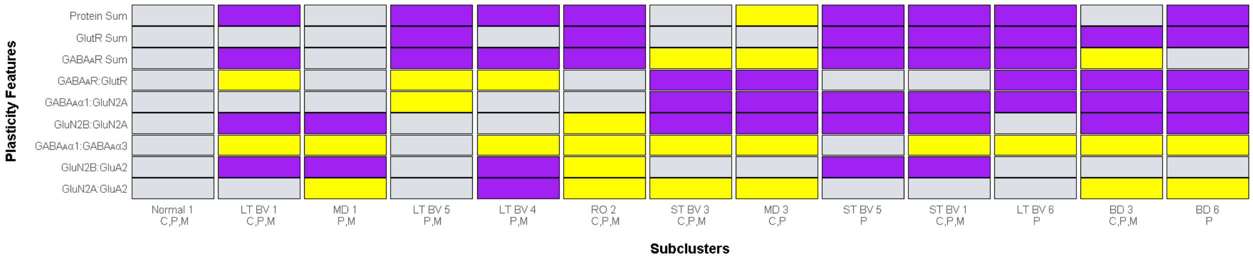

Create an ORA_phenotype using the object, “tsne.processed.4[,2:10]” with percentile values: "c(0.10,0.90)".

```
ORA_phenotype(
  features_df_row  = tsne.processed.4[,2:10], # Data frame for bootstrap analysis
  condition_list = as.list(c('Normal 1\n C,P,M',
                     'LT BV 1\n C,P,M',
                     'MD 1\n P,M',
                     'LT BV 5\n P,M',
                     'LT BV 4\n P,M',
                     'RO 2\n C,P,M',
                     'ST BV 3\n C,P,M',
                     'MD 3\n C,P',
                     'ST BV 5\n P',
                     'ST BV 1\n C,P,M',
                     'LT BV 6\n P',
                     'BD 3\n C,P,M',
                     'BD 6\n P')), # List of subclusters as they appear in row names of "features_df_row"
  reference_group =  'Normal 1\n C,P,M', # Name of reference group as it appears in the row names of "features_df_row"
  group_label  = "\nSubclusters", # X-axis label

  percentiles  = c(0.10,0.90)  # Thresholds to use for experimental subclusters when performing ORA against reference subclusters

)
```

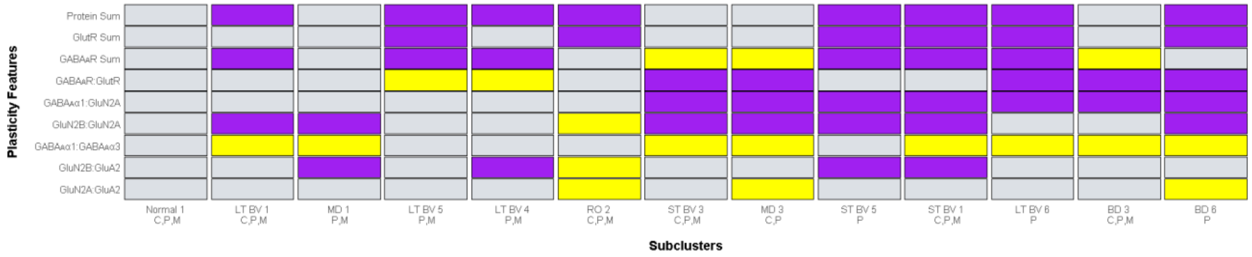

Cat_tSNE_plots_Figure7

Table of Contents

Section Number	Title	Page Number
1	Load Cat tSNE Data	53
2	Clustering of Experience-Dependent Changes in V1 Using a Plasticity Phenotype	56
2a	Create tSNE scatter plot: Plain	56
2b	Create tSNE scatter plot: Elbow Plot	57
2c	Create tSNE scatter plot: Cluster-Coded	58
2d	Create tSNE scatter plot: Region-Coded	59
2e	Create tSNE scatter plot: Condition- & Region-Coded	61

1.) Load Cat tSNE Data

Load relevant packages and data files

Install the package, “PlasticityPhenotypes”.

```
devtools::install_github("visualneurosciencelab/PlasticityPhenotypes")
```

Load the package, “PlasticityPhenotypes”.

```
library(PlasticityPhenotypes)

## Loading required package: tidyverse

## -- Attaching packages ------------------------------------------ tidyverse
1.3.0 --

## v ggplot2 3.3.0     v purrr   0.3.3
## v tibble  2.1.3     v dplyr   0.8.5
## v tidyr   1.0.2     v stringr 1.4.0
## v readr   1.3.1     v forcats 0.5.0

## -- Conflicts --------------------------------------------- tidyverse_confl
icts() --
## x dplyr::filter() masks stats::filter()
## x dplyr::lag()    masks stats::lag()

## Loading required package: FactoMineR

## Loading required package: factoextra

## Welcome! Want to learn more? See two factoextra-related books at https://g
oo.gl/ve3WBa

## Loading required package: data.table

##
## Attaching package: 'data.table'

## The following objects are masked from 'package:dplyr':
##
##     between, first, last

## The following object is masked from 'package:purrr':
##
##     transpose

## Loading required package: ggpubr
```

```
## Loading required package: magrittr

##
## Attaching package: 'magrittr'

## The following object is masked from 'package:purrr':
##
##     set_names

## The following object is masked from 'package:tidyr':
##
##     extract

## Loading required package: corrplot

## corrplot 0.84 loaded

## Loading required package: psych

##
## Attaching package: 'psych'

## The following objects are masked from 'package:ggplot2':
##
##     %+%, alpha

## Loading required package: httr
```

Store file paths in unique objects.

```
raw.cat.tsne <- 'https://osf.io/59yu6//?action=download'
wss <- 'https://osf.io/t73fc//?action=download'
```

Import cat subcluster/tSNE data from OSF, and store them in the object, “tsne.raw.data”. This data frame consists of 6 attributes (“Condition”, “Labels”, “Cluster.Number”, “Reordered”, “Cluster.Name”, & “Region”), and 2 tSNE X & Y coordinates. The object “elbow.data” consists of one column for ascending cluster numbers (‘k’) and another for the within groups sums of squares of each cluster (“ss”).

```
filename <- 'cat_tsne.csv'
GET(raw.cat.tsne, write_disk(filename, overwrite = TRUE))

## Response [https://files.ca-1.osf.io/v1/resources/8a3kx/providers/osfstorag
e/5ed0684f17ac9e0316621448?action=download&direct&version=1]
##   Date: 2020-06-03 18:00
##   Status: 200
```

```
##   Content-Type: text/csv
##   Size: 13.9 kB
## <ON DISK>  C:\Users\dezia\Dropbox (Kathy Murphy)\JB-ProteinAnalysisWorkflo
w\JB ProteinAnalysisWorkflow Markdowns\RMD files\Cat Analysis\cat_tsne.csv
```

```
tsne.raw.data <- read.csv(filename)
head(tsne.raw.data)
```

```
##   Condition           Labels        V1       V2 Cluster.Number Reordered
## 1    Normal 5wk Normal CVF 1 -1.421038 1.926670              1         1
## 2    Normal 5wk Normal CVF 2 -1.182249 3.150573              1         1
## 3    Normal 5wk Normal MVF 1 -1.488770 1.005343              1         1
## 4    Normal 5wk Normal MVF 2 -1.511189 1.509622              1         1
## 5    Normal 5wk Normal PVF 1 -1.438462 3.133959              1         1
## 6    Normal 5wk Normal PVF 2 -1.956975 2.357059              1         1
##   Cluster.Name Region
## 1     Normal 1    CVF
## 2     Normal 1    CVF
## 3     Normal 1    MVF
## 4     Normal 1    MVF
## 5     Normal 1    PVF
## 6     Normal 1    PVF
```

```
filename <- 'and stored in the file wss.csv'
GET(wss, write_disk(filename, overwrite = TRUE))
```

```
## Response [https://files.ca-1.osf.io/v1/resources/8a3kx/providers/osfstorag
e/5ed7dce7237cd3009d54b26e?action=download&direct&version=1]
##   Date: 2020-06-03 18:00
##   Status: 200
##   Content-Type: text/csv
##   Size: 236 B
## <ON DISK>  C:\Users\dezia\Dropbox (Kathy Murphy)\JB-ProteinAnalysisWorkflo
w\JB ProteinAnalysisWorkflow Markdowns\RMD files\Cat Analysis\wss.csv
```

```
elbow.data <- read.csv(filename)
head(elbow.data)
```

```
##   k        ss
## 1 1 2744.0456
## 2 2 1407.4805
## 3 3  737.2642
## 4 4  642.9166
## 5 5  316.1098
## 6 6  228.6830
```

2.) Clustering of Experience-Dependent Changes in V1 Using a Plasticity Phenotype

Subset “tsne.raw.data” to only return observations where there are no NAs in the “Cluster.Number” column.

```
tsne.raw.data <- subset(tsne.raw.data,
                  is.na(tsne.raw.data$Cluster.Number) == F)
```

Set a standard size for data points and axis label size across all tSNE scatter plots.

```
ptsize = 5
txtsize = 20
```

Set a standard size for data points, axis tick labels, and axis titles on the elbow plot.

```
#data point size for elbow plot = "ptsize"
txtsize.elb = 12
titlesize.elb = 14
```

Create tSNE scatterplots

a.) Plain: Create a tSNE scatter plot with no mapped aesthetics.

```
noncolor <- ggplot(tsne.raw.data,
                         aes(x=V1,
                             y=V2))+
                    geom_point(size = ptsize)+
                    xlab("\ntSNE X")+
                    ylab("tSNE Y\n")+
                    theme_classic()+
                    theme(axis.text.x = element_blank(),
                          axis.text.y = element_blank(),
                          axis.ticks.x=element_blank(),
                          axis.ticks.y=element_blank(),
                          axis.title.x = element_text(size = txtsize, face =
'bold'),
                          axis.title.y = element_text(size = txtsize, face =
'bold'),
                           legend.position = "none")
```

```r

print(noncolor)
```

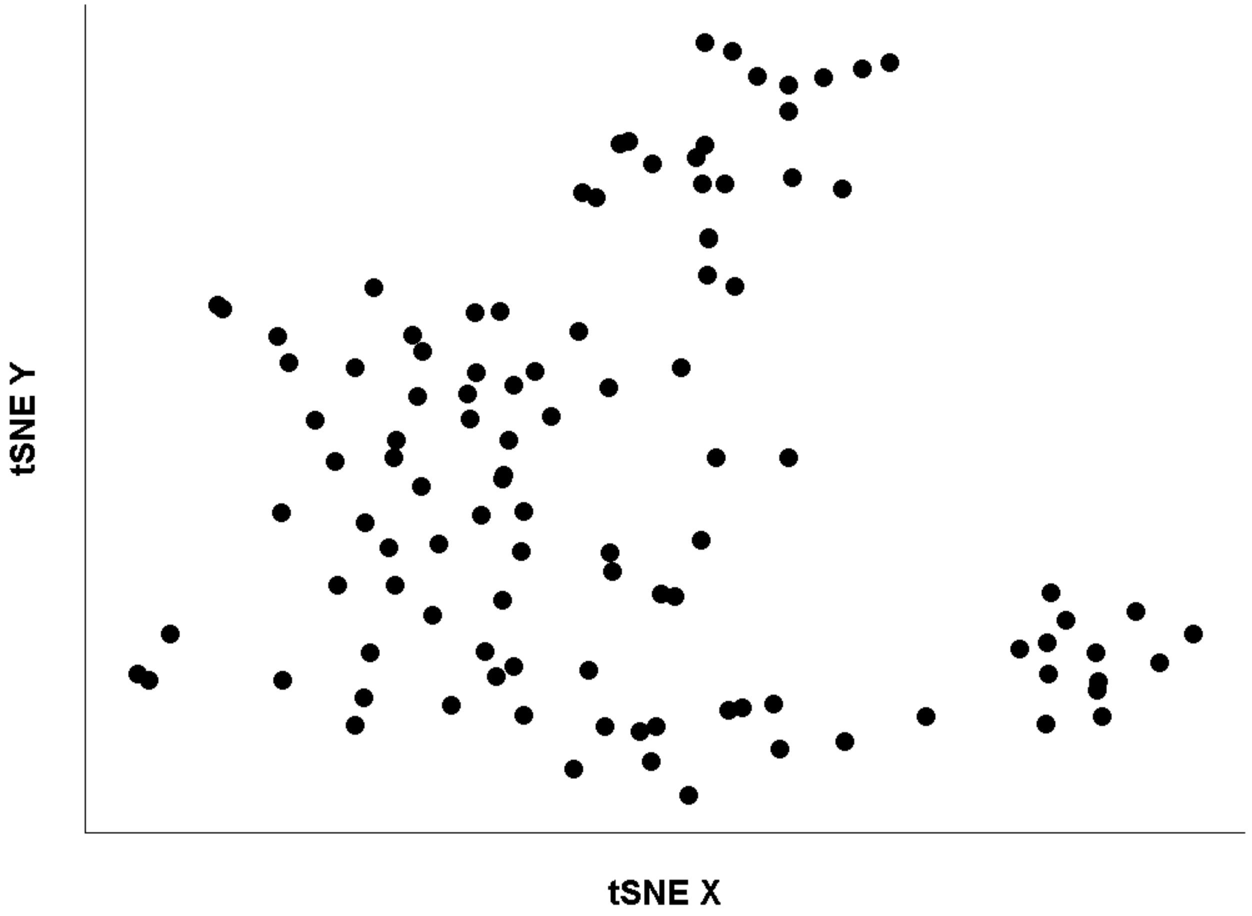

b.) Elbow Plot: Create an elbow plot that identifies how many clusters are required to minimize the within group sums of squares.

```r
elb.plot <- ggplot(elbow.data,aes(x = factor(k),
                                  y = ss,
                                  group = 1)) +
  xlab("\nNumber of Clusters") +
  ylab("Within Group Sums of Square (WGSS)\n") +
  theme_classic() +
  geom_line() +
  geom_point(shape=21, fill="white",
             color="black",
             size=ptsize,
             stroke = 1.3)+
  theme(axis.text.x = element_text(size = txtsize.elb),
        axis.text.y = element_text(size = txtsize.elb),
        axis.title.x = element_text(size = titlesize.elb, face = "bold"),
```

```
        axis.title.y = element_text(size = titlesize.elb, face = "bold"))
print(elb.plot)
```

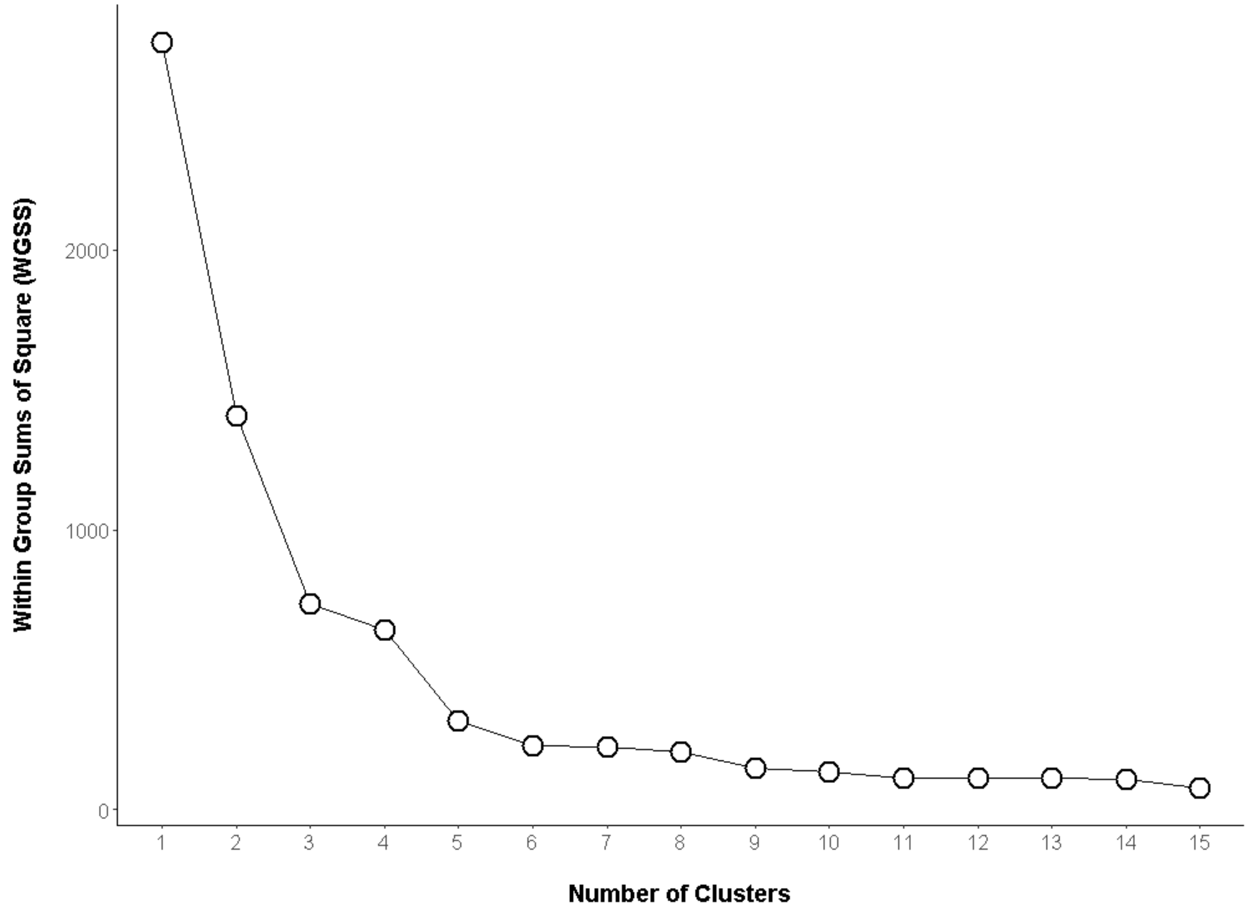

c.) Cluster-Coded: Create a tSNE scatter plot where points are coloured according to their cluster membership.

```
color <- ggplot(tsne.raw.data,
                aes(x=V1,
                    y=V2,
                    color = as.factor(Cluster.Number)))+
                geom_point(size = ptsize)+
                xlab("\ntSNE X")+
                ylab("tSNE Y\n")+
                theme_classic()+
                theme(axis.text.x = element_blank(),
                      axis.text.y = element_blank(),
                      axis.ticks.x=element_blank(),
                      axis.ticks.y=element_blank(),
                      axis.title.x = element_text(size = txtsize, face = 'bol
d'),
                      axis.title.y = element_text(size = txtsize, face = 'bol
```

```
d')
                      ,legend.position = "none"
                      )+scale_color_manual(values = c('orange',
                                                      'green',
                                                      '#15F4EE',
                                                      '#E1AD01',
                                                      'purple',
                                                      '#FF00FF'))
print(color)
```

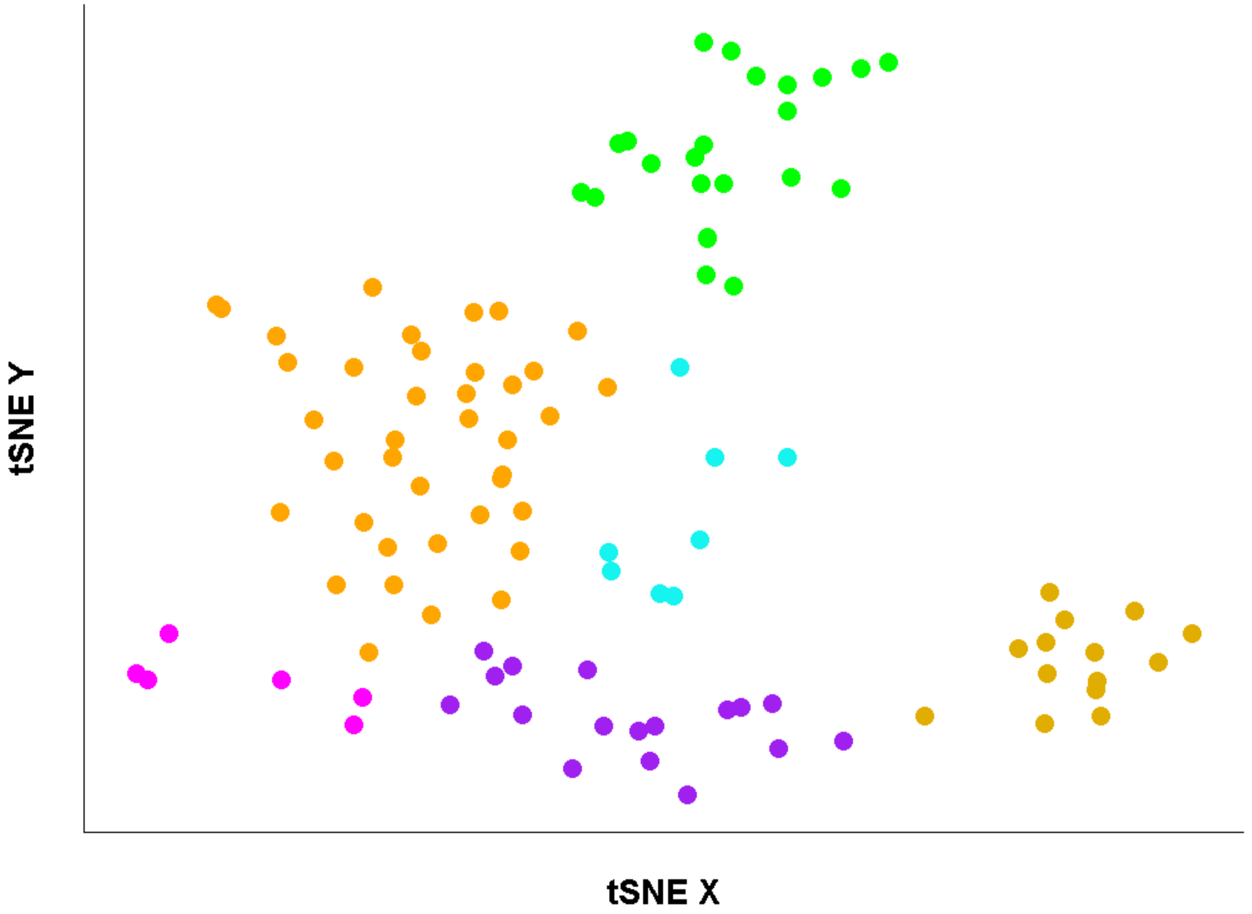

d.) Region-Coded: Create a tSNE scatter plot where points are shaped according to the region of the visual cortex they were sampled from.

```
shapes <-              ggplot(tsne.raw.data,
                          aes(x=V1,
                              y=V2,
                              shape = Region,
                              fill = Region))+
                          geom_point(size = ptsize)+
                          xlab("\ntSNE X")+
```

```
                          ylab("tSNE Y\n")+
                          theme_classic()+
                          theme(axis.text.x = element_blank(),
                                axis.text.y = element_blank(),
                                axis.ticks.x=element_blank(),
                                axis.ticks.y=element_blank(),
                                axis.title.x = element_text(size = txtsize, f
ace = 'bold'),
                                axis.title.y = element_text(size = txtsize, f
ace = 'bold'),
                                legend.position = "none")+
                        scale_shape_manual(values = c(21,22,24))+
                        scale_fill_manual(values = c('black','white','grey'))
print(shapes)
```

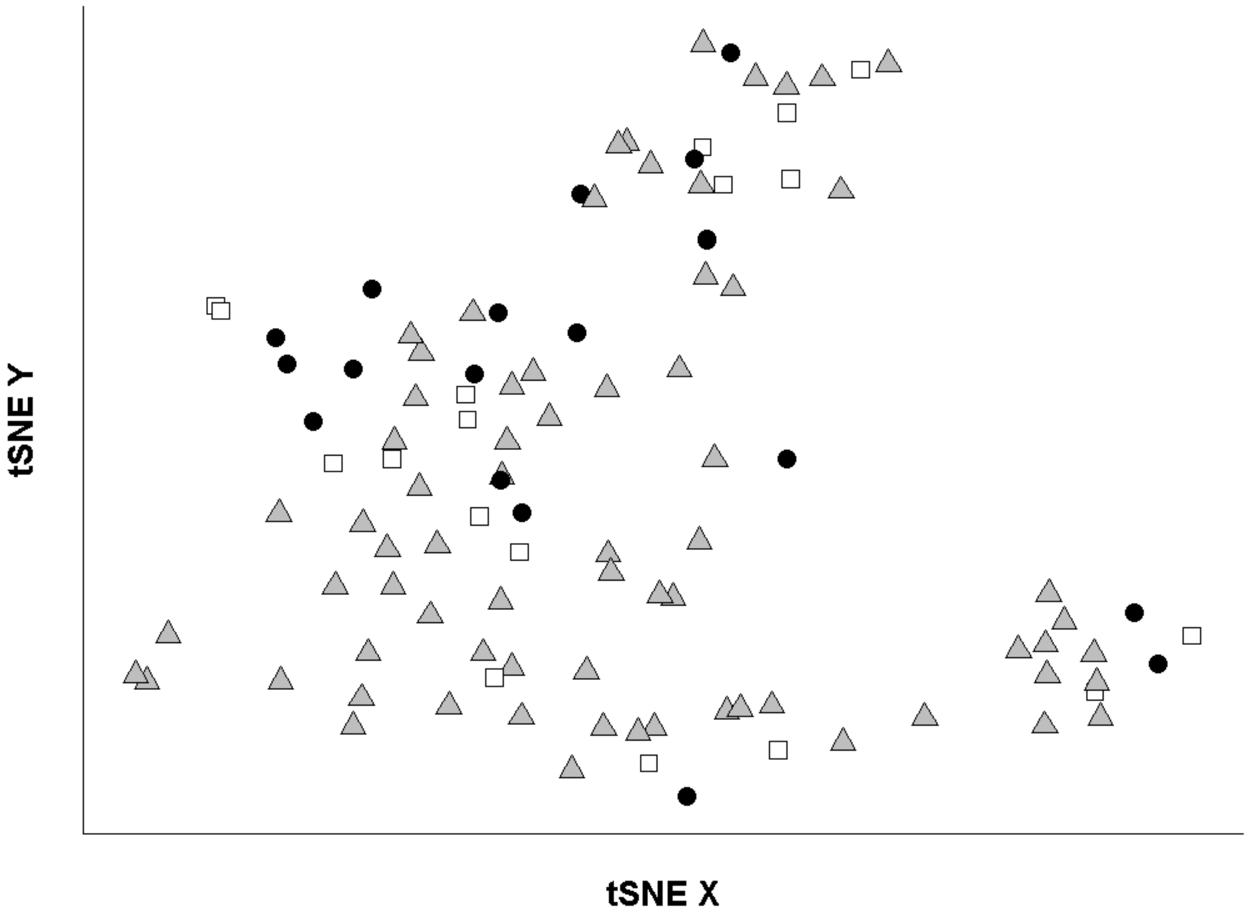

e.) Condition- & Region-Coded: Create a tSNE scatter plot where points are shaped according to the region of the visual cortex they were sampled from, and coloured based on which condition they were in.

```
col.shapes  <- ggplot(tsne.raw.data,
                          aes(x=V1,
                              y=V2,
                              shape = Region,
                              fill = Condition))+
                          geom_point(size = ptsize)+
                          xlab("\ntSNE X")+
                          ylab("tSNE Y\n")+
                          theme_classic()+
                          theme(axis.text.x = element_blank(),
                                axis.text.y = element_blank(),
                                axis.ticks.x=element_blank(),
                                axis.ticks.y=element_blank(),
                                axis.title.x = element_text(size = txtsize, f
ace = 'bold'),
                                axis.title.y = element_text(size = txtsize, f
ace = 'bold'),
                                legend.position = "none")+
                          scale_shape_manual(values = c(21,22,24))+
                          scale_fill_manual(values = c('green',
                                                       'red',
                                                       'grey',
                                                       'black',
                                                       'blue',
                                                       'pink'))
print(col.shapes)
```

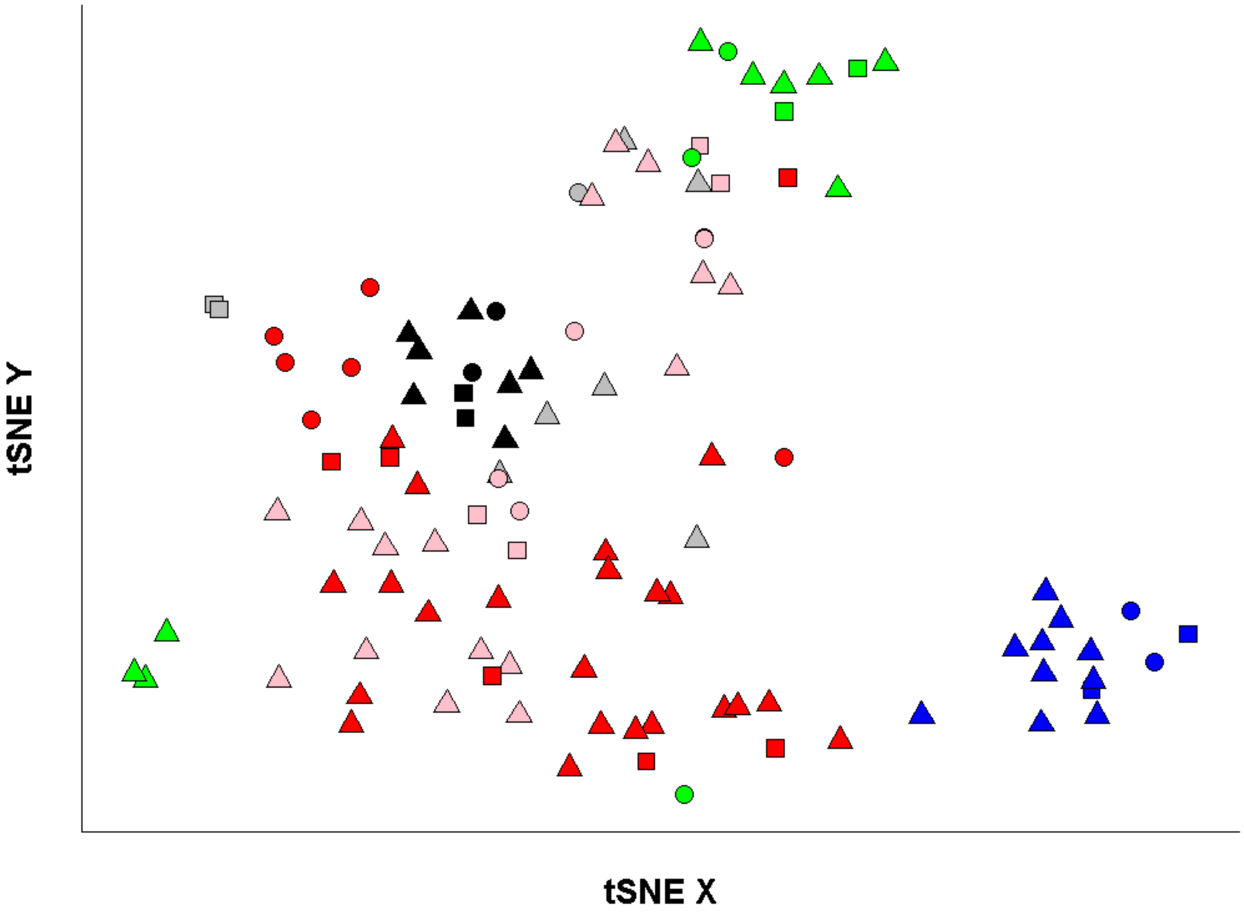

247

Cat_CorrHeatmap_Figure8

Table of Contents		
**Section Number**	**Title**	**Page Number**
1	Load Cat Correlation Data	64
2	Process the Cat tSNE Correlation Data	67
3	Identifying and Exploring Subclusters using Plasticity Features and Phenotypes – Create Heatmap	68

1.) Load Cat Correlation Data

Install the package, “PlasticityPhenotypes”.

```
devtools::install_github("visualneurosciencelab/PlasticityPhenotypes")
```

Load the package, “PlasticityPhenotypes”.

```
library(PlasticityPhenotypes)

## Loading required package: tidyverse

## -- Attaching packages -------------------

## v ggplot2 3.3.0     v purrr   0.3.3
## v tibble  2.1.3     v dplyr   0.8.5
## v tidyr   1.0.2     v stringr 1.4.0
## v readr   1.3.1     v forcats 0.5.0

## -- Conflicts --- tidyverse_conflicts() --
## x dplyr::filter() masks stats::filter()
## x dplyr::lag()    masks stats::lag()

## Loading required package: FactoMineR

## Loading required package: factoextra

## Welcome! Want to learn more? See two factoextra-related books at https://g
oo.gl/ve3WBa

## Loading required package: data.table

##
## Attaching package: 'data.table'

## The following objects are masked from 'package:dplyr':
##
##     between, first, last

## The following object is masked from 'package:purrr':
##
##     transpose

## Loading required package: ggpubr

## Loading required package: magrittr

##
## Attaching package: 'magrittr'
```

```
## The following object is masked from 'package:purrr':
##
##     set_names

## The following object is masked from 'package:tidyr':
##
##     extract

## Loading required package: corrplot

## corrplot 0.84 loaded

## Loading required package: psych

##
## Attaching package: 'psych'

## The following objects are masked from 'package:ggplot2':
##
##     %+%, alpha

## Loading required package: httr

library(gplots)

##
## Attaching package: 'gplots'

## The following object is masked from 'package:stats':
##
##     lowess

library(RColorBrewer)
library(Hmisc)

## Loading required package: lattice

## Loading required package: survival

## Loading required package: Formula

##
## Attaching package: 'Hmisc'

## The following object is masked from 'package:psych':
##
##     describe

## The following objects are masked from 'package:dplyr':
##
##     src, summarize
```

```
## The following objects are masked from 'package:base':
##
##     format.pval, units
```

Store the file path in a unique object.

```
raw.cat.corr <- "https://osf.io/5d4kt//?action=download"
```

Import the necessary CSV from OSF. The “raw.data” object consists of 10 columns – 1 attributes column (“Cluster.Name”) and 9 plasticity feature columns.

```
filename <- 'cat_correlation.csv'
GET(raw.cat.corr, write_disk(filename, overwrite = TRUE))
```

```
## Response [https://files.ca-1.osf.io/v1/resources/8a3kx/providers/osfstorag
e/5ecd57e117ac9e01fa62506b?action=download&direct&version=1]
##   Date: 2020-06-02 19:45
##   Status: 200
##   Content-Type: text/csv
##   Size: 17.9 kB
## <ON DISK>  C:\Users\dezia\Dropbox (Kathy Murphy)\JB-ProteinAnalysisWorkflo
w\JB ProteinAnalysisWorkflow Markdowns\RMD files\Cat Analysis\cat_correlation
.csv
```

```
raw.data <- read.csv(filename, row.names = 1)
head(raw.data)
```

```
##               Cluster.Name Protein.Sum. GlutR.Sum. GABA.U.1D00.R.Sum.
## 18d RO CVF 1          RO 2     9.326704   5.307563           2.142932
## 18d RO CVF 2          RO 2    10.556702   5.316230           2.694749
## 18d RO MVF 1          RO 2     9.658435   5.497712           2.100966
## 18d RO MVF 2          RO 2     8.259729   4.771556           1.727323
## 18d RO PVF 1          RO 2     9.216756   4.490773           2.830239
## 18d RO PVF 10         RO 2     8.754209   5.089096           1.522142
##               GABA.U.1D00.R.GlutR. GABA.U.1D00.a1.GluN2A. GluN2B.GluN2A.
## 18d RO CVF 1            0.10649975             0.11897191      0.2801335
## 18d RO CVF 2           -0.00684382            -0.02566512      0.3031057
## 18d RO MVF 1            0.13359050             0.06702033      0.2921762
## 18d RO MVF 2            0.16008720             0.09124979      0.3333868
## 18d RO PVF 1           -0.11522762            -0.17862038      0.2575936
## 18d RO PVF 10           0.25140999             0.29200612      0.3675670
##               GABA.U.1D00.a1.GABA.U.1D00.a3. GluN2B.GluA2. GluN2A.GluA2.
## 18d RO CVF 1                       0.02462795     0.3838222     0.1161806
## 18d RO CVF 2                       0.05244266     0.4242947     0.1390749
## 18d RO MVF 1                       0.06432353     0.5487068     0.3055096
## 18d RO MVF 2                       0.16977656     0.5336853     0.2436495
## 18d RO PVF 1                       0.10214623     0.4249325     0.1879072
## 18d RO PVF 10                      0.03174592     0.4604927     0.1118592
```

2.) Process the Cat tSNE Correlation Data

Store contents of "raw.data$Cluster.Name" as a data frame in the object, "ids.3".

```
ids.3 <- as.data.frame(raw.data$Cluster.Name)
```

Rename "ids.3" column header.

```
colnames(ids.3) <- 'sample.ids'
```

Calculate the median of each feature across subclusters, and store in 'med.df'.

```
all.df <- group_by(raw.data,ids.3$sample.ids)

med.df <- summarise_at(all.df,vars(colnames(raw.data)[-1]),median)
```

Rename the first column of "med.df" to "subcluster". Transform the "med.df$subcluster" from a column to individual row names. Store the output into the object,"med.df.2".

```
colnames(med.df)[1] <- 'subcluster'

med.df.2 <- column_to_rownames(med.df,var = 'subcluster')
```

Transpose the "med.df.2", and store in "t.med.df".

```
t.med.df <- as.matrix(t(med.df.2))
```

3.) Exploring Subclusters using Plasticity Phenotypes – Create Heatmap

Perform a Pearson's correlation against all subclusters to each other, and store in "data.correlation".

```
data.correlation <- rcorr(as.matrix(t.med.df),
                          type ="pearson")
```

Save correlations coefficients as a matrix in "CorMat".

```
CorMat <- as.matrix(data.correlation$r)
```

Create custom color palette for heatmap.

```
my_palette <- colorRampPalette(c(
                  "blue",
                  '#00CED1',
                  "green",
                  "yellow",
                  "orange",
                  "red"))(n = 6000)
```

Sets heatmap margins.

```
par(oma=c(rep(7,4)))
```

Create a character vector specifying the order of subclusters, called "clust_order".

```
clust_order <- c("Normal 1",
                 "LT BV 1",
                 "MD 1",
                 "LT BV 5",
                 "LT BV 4",
                 "RO 2",
                 "ST BV 3",
                 "MD 3",
                 "ST BV 5",
                 "ST BV 1",
                 "LT BV 6",
                 "BD 3",
                 "BD 6")
```

Creates heatmap.

```
heatmap.2(CorMat[clust_order,
                 clust_order],
          cexRow = 1,
          Colv = F,
          Rowv = F,
          colsep=c(5,
                   6,
                   10,
                   11,
                   12,
                   13),
          rowsep=c(5,
                   6,
                   10,
                   11,
                   12,
                   13),
          cexCol=1,
          sepcolor="black",
          density.info="none",
          trace="none",
          col = my_palette,
          revc = T,
          symm=T)
```

```
## Warning in heatmap.2(CorMat[clust_order, clust_order], cexRow = 1, Colv =
F, :
## Discrepancy: Rowv is FALSE, while dendrogram is `both'. Omitting row dendo
gram.
```

```
## Warning in heatmap.2(CorMat[clust_order, clust_order], cexRow = 1, Colv =
## F, : Discrepancy: Colv is FALSE, while dendrogram is `column'. Omitting co
lumn
## dendogram.
```

```
## Warning in plot.window(...): "revc" is not a graphical parameter
```

```
## Warning in plot.xy(xy, type, ...): "revc" is not a graphical parameter
```

```
## Warning in title(...): "revc" is not a graphical parameter
```

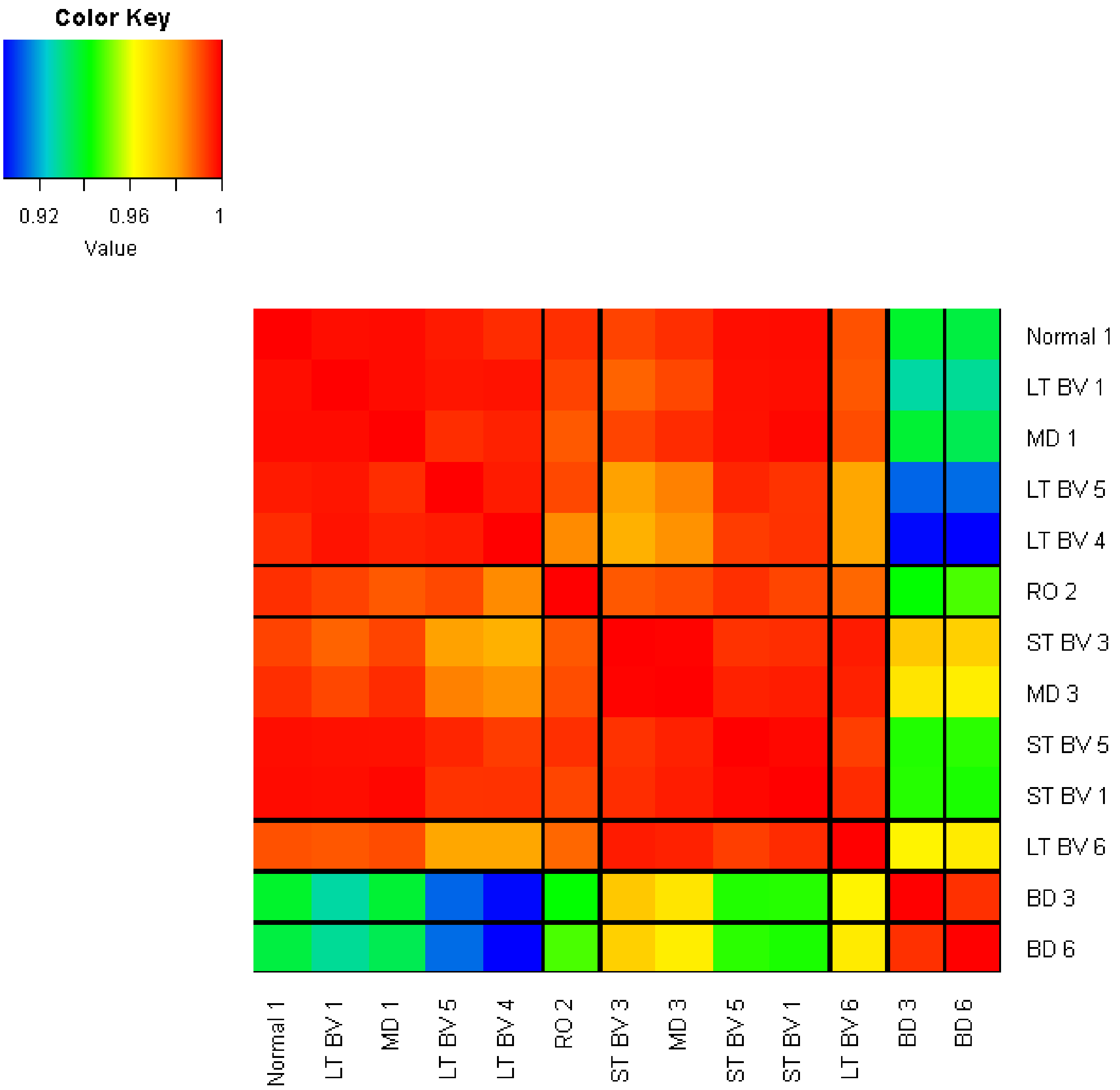
Color Key
0.92
0.96
1
Value
Normal 1
LT BV 1
MD 1
LT BV 5
LT BV 4
RO 2
ST BV 3
MD 3
ST BV 5
ST BV 1
LT BV 6
BD 3
BD 6

200

Rat_Markdown

Table of Contents		
**Section Number**	**Title**	**Page Number**
1	Load Rat Data	72
2	Rat Analysis	75
3	Rat Phenotype	91
4	Create Rat Boxplots	93
5	Rat Plasticity Phenotype Bootstrap Analysis	96

1.) Load Rat Data

Rat: Dimension Reduction using PCA – Data Processing

Install the package, “PlasticityPhenotypes”.

```
devtools::install_github("visualneurosciencelab/PlasticityPhenotypes")
```

Load the package, “PlasticityPhenotypes”.

```
library(PlasticityPhenotypes)

## Loading required package: tidyverse

## -- Attaching packages --------------------------------------------------- tid
yverse 1.3.0 --

## v ggplot2 3.3.0     v purrr   0.3.3
## v tibble  2.1.3     v dplyr   0.8.5
## v tidyr   1.0.2     v stringr 1.4.0
## v readr   1.3.1     v forcats 0.5.0

## -- Conflicts --------------------------------------------------- tidyverse
_conflicts() --
## x dplyr::filter() masks stats::filter()
## x dplyr::lag()    masks stats::lag()

## Loading required package: FactoMineR

## Loading required package: factoextra

## Welcome! Want to learn more? See two factoextra-related books at https://g
oo.gl/ve3WBa

## Loading required package: data.table

##
## Attaching package: 'data.table'

## The following objects are masked from 'package:dplyr':
##
##     between, first, last

## The following object is masked from 'package:purrr':
##
##     transpose

## Loading required package: ggpubr

## Loading required package: magrittr
```

```
##
## Attaching package: 'magrittr'

## The following object is masked from 'package:purrr':
##
##     set_names

## The following object is masked from 'package:tidyr':
##
##     extract

## Loading required package: corrplot

## corrplot 0.84 loaded

## Loading required package: psych

##
## Attaching package: 'psych'

## The following objects are masked from 'package:ggplot2':
##
##     %+%, alpha

## Loading required package: httr
```

Store the file path in a unique object.

```
raw.rat <- 'https://osf.io/d5pzt//?action=download'
```

Import the necessary CSV. The "raw.data" object consists of 5 attribute columns ("ID", "Condition", "Region", "Hemisphere", & "Run"), and 10 columns of distinct proteins' expression.

```
filename <- 'rat_protein.csv'
GET(raw.rat, write_disk(filename, overwrite = TRUE))

## Response [https://files.ca-1.osf.io/v1/resources/8a3kx/providers/osfstorag
e/5ece9211aeeb6d025a085f0d?action=download&direct&version=3]
##   Date: 2020-06-09 23:53
##   Status: 200
##   Content-Type: text/csv
##   Size: 12 kB
## <ON DISK>  C:\Users\dezia\Dropbox (Kathy Murphy)\JB-ProteinAnalysisWorkflo
w\JB ProteinAnalysisWorkflow Markdowns\RMD files June 2020\Rat Analysis\Curre
nt\rat_protein.csv

raw.data <- read.csv(filename)
head(raw.data)
```

```
##   Case_Code Condition Region Hemisphere Run     GluA2     GluN1    GluN2A
## 1       231  Prozac_N     VC       Cont   1 1.0365590 0.5287500 0.9606986
## 2       232  Prozac_N     VC       Cont   1 1.1490010 0.9744765 0.9863363
## 3       233  Prozac_N     VC       Cont   1 1.1218811 1.5784559 1.5190979
## 4       234  Prozac_N     VC       Cont   1 1.0465851 1.0153453 0.8924184
## 5       235  Prozac_N     VC       Cont   1 0.8387192 0.7336301 0.3954039
## 6       236  Prozac_N     VC       Cont   1 1.2681864 1.1609523 1.0274127
##      GluN2B   GABAAa1   GABAAa3     PSD95  Gephyrin     VGLUT      VGAT
## 1 0.6609931 0.6081925 1.0732763 0.5933443 1.0182930 0.6543510 0.6877782
## 2 0.8524244 1.0276800 1.0655188 2.4078841 2.5542893 0.8957831 0.9694615
## 3 0.8402657 1.8131427 1.1594168 0.6794247 1.1658637 0.8531914 0.9891639
## 4 0.6999245 1.1133509 0.8682642 1.0641081 2.0030711 0.6892650 0.8166134
## 5 0.4312522 0.8327478 0.5771284 0.9394761 1.4427779 0.3932702 0.6479182
## 6 1.3597457 1.2145710 1.0280006 0.6317986 0.9063774 0.9291042 1.2881240
```

2.) Rat Analysis

Rat: Dimension Reduction Using PCA – Additional Processing & Analysis

Rename the levels of “raw.data$Condition" into a manner consistent with (Beshara et al., 2015), and reassign to "raw.data$Condition”.

```
raw.data$Condition <- plyr::mapvalues(raw.data$Condition,
                                      unique(raw.data$Condition),
                                      c('flx',
                                        'flx + 1wk MD',
                                        'normal',
                                        '1wk MD'))
```

Rename protein data columns of “raw.data” that contain special characters.

```
colnames(raw.data)[10:11] <- c("GABA\u1D00\u03b11",
                               'GABA\u1D00\u03b13')
```

Retrieve cases without missing values in “raw.data[complete.cases(raw.data),]”, and reassign them to the object, “syn.prots”.

```
syn.prots <- raw.data[complete.cases(raw.data),]
```

Assign the protein columns of “syn.prots” to the new object, “my.data”.

```
my.data <- syn.prots[,c("GluA2",
                        "GluN1",
                        "GluN2A",
                        "GluN2B",
                        "GABA\u1D00\u03b11",
                        "GABA\u1D00\u03b13",
                        "Gephyrin",
                        "PSD95",
                        "VGLUT",
                        "VGAT")]
```

Concatenate attribute columns of "syn.prots" and assign as distinct row names of "my.data".

```
rownames(my.data) <- paste(syn.prots$Case_Code,
                           syn.prots$Condition,
                           syn.prots$Region,
                           syn.prots$Hemisphere,
                           syn.prots$Run, sep = " ")
```

Centre data and scale the contents of "my.data", and assign them to the object, "my.data.scaled".

```
my.data.scaled <- scale(my.data,
                     center = TRUE,
                     scale = TRUE)
```

Perform PCA on "my.data.scaled", and store in the object, "pca.scaled".

```
pca.scaled <- PCA(my.data.scaled,
                  ncp=ncol(my.data.scaled),
                  scale.unit=FALSE,
                  graph = FALSE)
```

Construct scree plot.

```
fviz_eig(pca.scaled,
         ylim = c(0, 60),
         xlim = c(0.5,7.5),
         ncp = 7, # Select number of principal components using 'ncp' parameter
         barfill = "grey",
         barcolor = "grey",
         geom = "bar"#, text
         )+
  scale_y_continuous(expand = c(0,0))+
  scale_x_discrete(expand = c(0,0))+
  theme(axis.line.y=element_line(),
        axis.line.x=element_line(),
        panel.grid=element_blank(),
        axis.text.x = element_text(size = 20),
        axis.text.y = element_text(size = 20),
        text = element_text(size = 25)
        )+
  xlab("\nDimensions")+
  ylab("Percentage of Explained Variance\n")+
  ggtitle("Scree Plot\n")
```

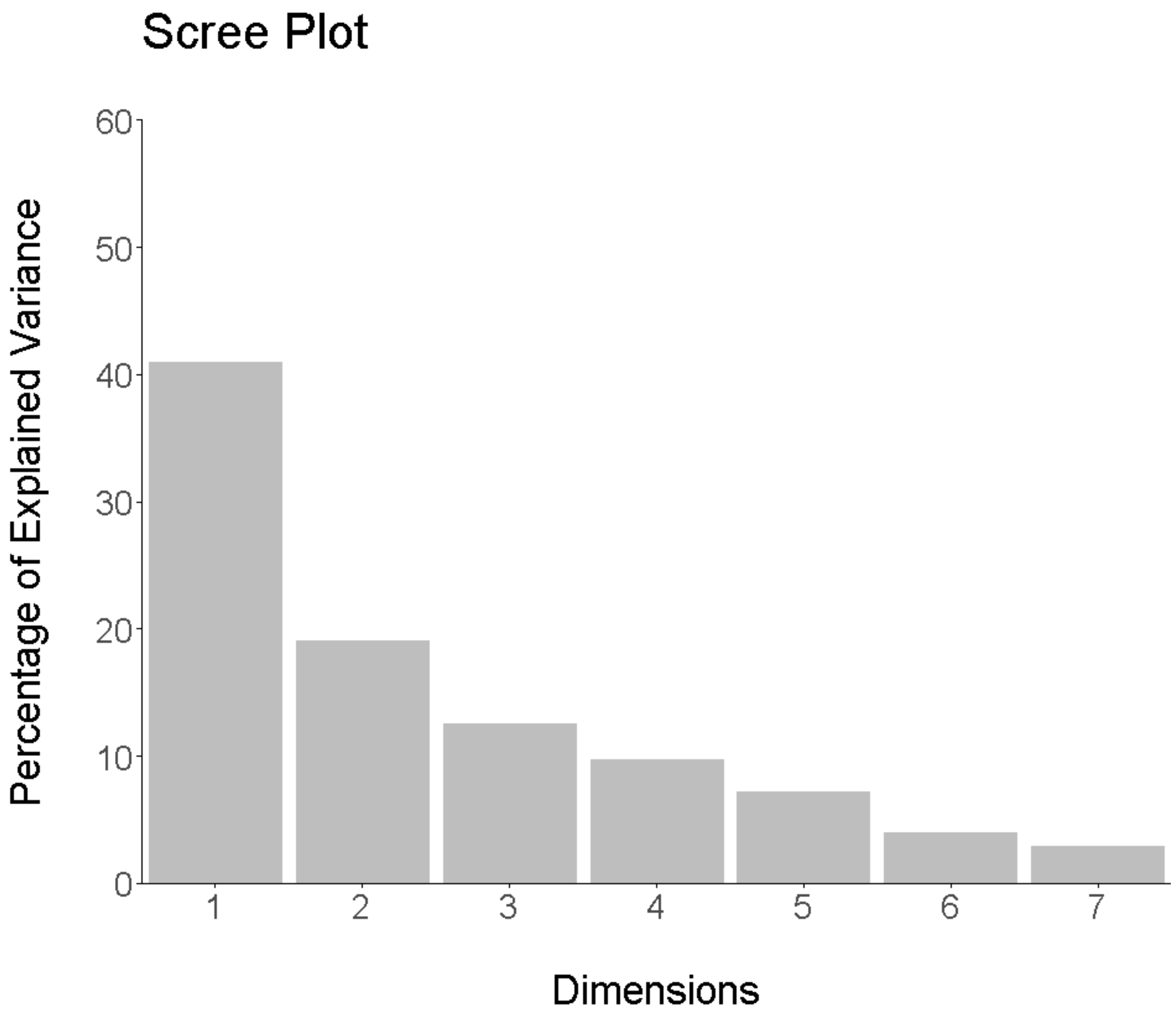

Rat: Identifying Candidate Plasticity Features

Construct plot of the cos^2 data stored in the object, “pca.scaled”.

```
pca.scaled$var$cos2
```

```
##                   Dim.1       Dim.2       Dim.3        Dim.4       Dim.5
## GluA2        0.76474184 0.030976705 0.004973224 3.038472e-02 0.004758510
## GluN1        0.74240651 0.002971197 0.022233983 6.479174e-03 0.088372864
## GluN2A       0.44898824 0.090426240 0.239947534 9.384183e-03 0.088170565
## GluN2B       0.48360386 0.179034865 0.069430784 2.961603e-03 0.144965377
## GABA<U+1D00>a1 0.06119843 0.072946897 0.040036126 7.031178e-01 0.112828020
## GABA<U+1D00>a3 0.63841652 0.010636313 0.144280232 6.277124e-07 0.009637956
## Gephyrin     0.15196515 0.686260112 0.054970569 1.328582e-02 0.036682070
## PSD95        0.06444387 0.825600954 0.017532551 2.944617e-02 0.005298097
## VGLUT        0.72309081 0.002824324 0.066308229 3.061224e-02 0.025621447
## VGAT         0.01576539 0.008665241 0.593194429 1.474058e-01 0.207785267
```

```
##                         Dim.6        Dim.7        Dim.8        Dim.9       Dim
.10
## GluA2        0.1032742691 0.0009677872 0.0222832077 0.0291530529 8.486683e
-03
## GluN1        0.0814380106 0.0001801498 0.0111629190 0.0434232666 1.331923e
-03
## GluN2A       0.0005045302 0.0940226829 0.0251046288 0.0014452982 2.006101e
-03
## GluN2B       0.0195524884 0.0680180183 0.0176440849 0.0131112895 1.677633e
-03
## GABA<U+1D00>a1 0.0026940292 0.0061893560 0.0003928626 0.0003983653 1.98077
9e-04
## GABA<U+1D00>a3 0.0885853506 0.0767075293 0.0261080521 0.0029823856 2.64503
0e-03
## Gephyrin     0.0032132792 0.0092461556 0.0094056857 0.0010813466 3.388981e
-02
## PSD95        0.0077746916 0.0002620205 0.0159275404 0.0016589820 3.205513e
-02
## VGLUT        0.0632995867 0.0317297777 0.0304004013 0.0208645253 5.248660e
-03
## VGAT         0.0243190008 0.0003393269 0.0020497922 0.0004751795 6.018825e
-07

corrplot(pca.scaled$var$cos2,is.corr=FALSE)
```

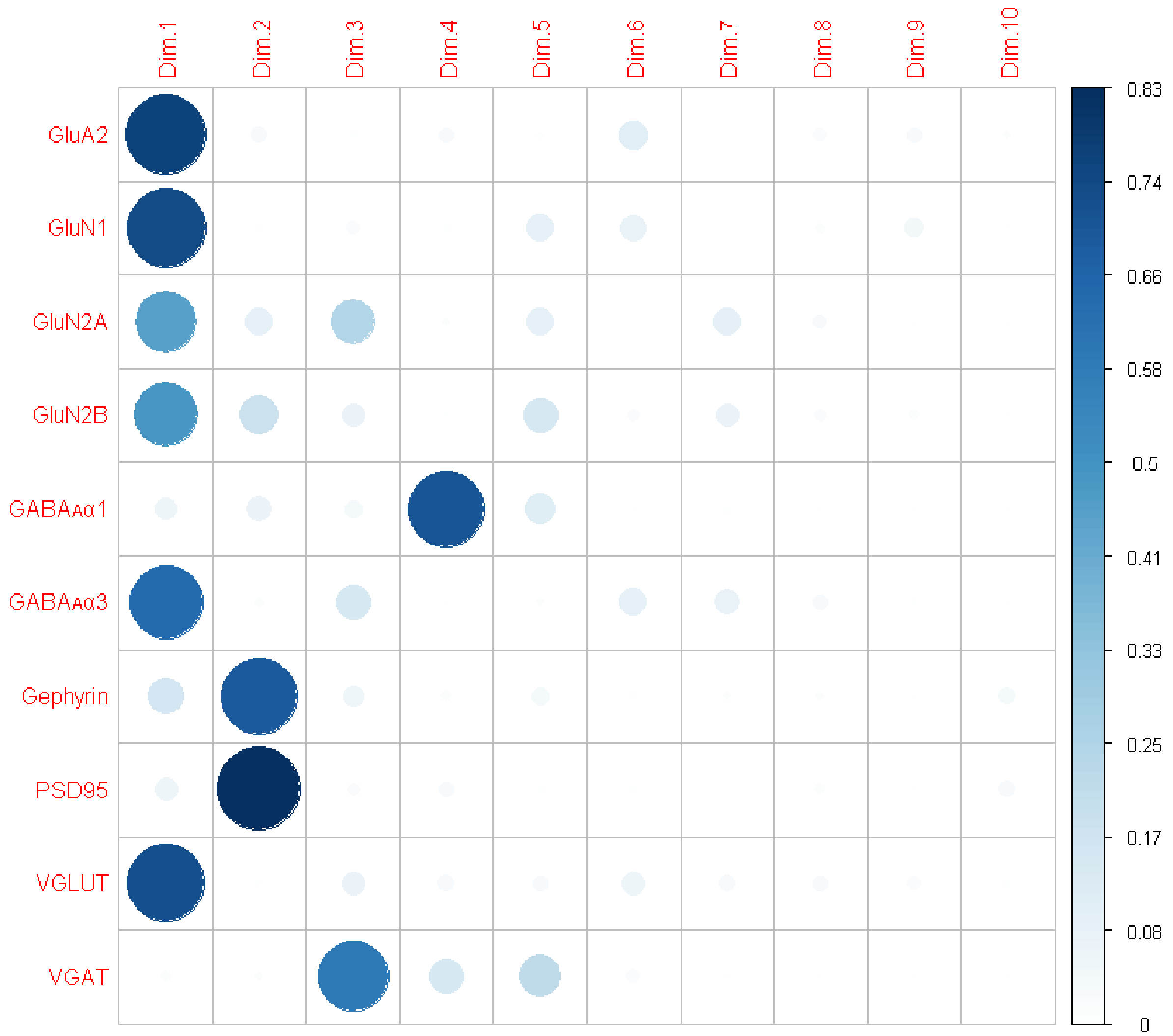

Construct a correlation plot of coord data stored in the object, “pca.scaled”.

```
head(pca.scaled$var$coord)
```

```
##                    Dim.1       Dim.2       Dim.3         Dim.4       Dim.5
## GluA2          0.8687981  0.17485540 -0.07006166 -0.1731765424  0.06853255
## GluN1          0.8560169 -0.05415358  0.14813922 -0.0799689248  0.29533907
## GluN2A         0.6657005  0.29875052 -0.48665319  0.0962408982 -0.29500083
## GluN2B         0.6908859  0.42036857 -0.26178061 -0.0540660776 -0.37826275
## GABA<U+1D00>a1 0.2457715  0.26832729 -0.19878676  0.8330584803  0.33371053
## GABA<U+1D00>a3 0.7938044 -0.10246062  0.37736781 -0.0007871216  0.09753352
##                    Dim.6       Dim.7       Dim.8       Dim.9      Dim.10
## GluA2         0.31926955 -0.03090661 -0.14830312  0.16963031 -0.09152304
## GluN1         0.28351433 -0.01333455  0.10496640 -0.20702495  0.03625776
## GluN2A        0.02231542  0.30463357  0.15741218  0.03776941  0.04449773
```

```
## GluN2B        -0.13891926 -0.25910358 -0.13196568 -0.11375857  0.04069209
## GABA<U+1D00>a1 -0.05156590 -0.07815993 -0.01969163  0.01982906 -0.01398233
## GABA<U+1D00>a3 -0.29569392  0.27515691 -0.16052721 -0.05425544 -0.05109481
```

```r
corrplot(pca.scaled$var$coord, is.corr= TRUE)
```

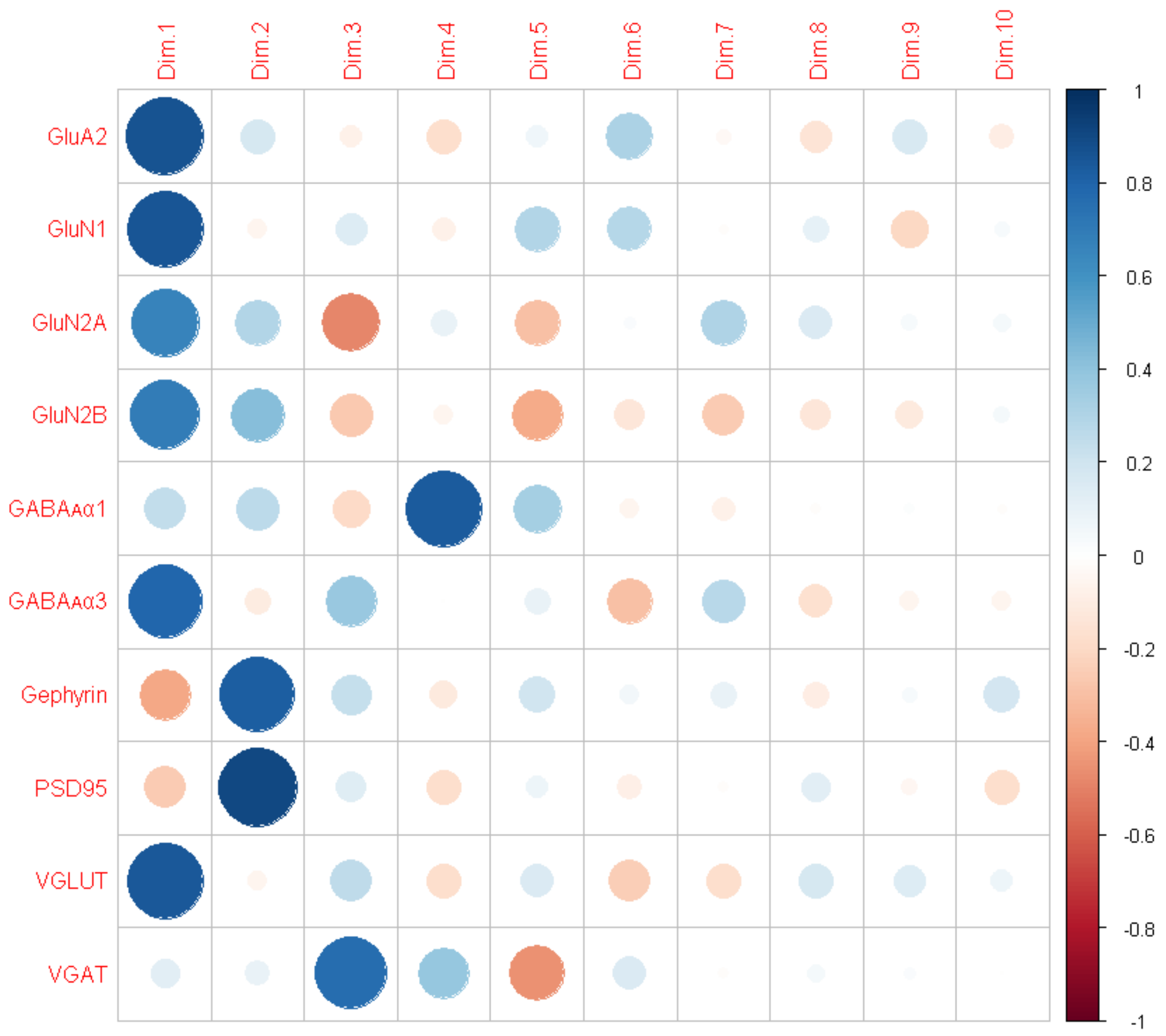

Calculate how many components are required to maintain 80% of the total variance. Store the output in the object, "cum.var".

```r
pca.scaled$eig[,3]
```

```
##    comp 1    comp 2    comp 3    comp 4    comp 5    comp 6    comp 7    c
omp 8
##  40.94621  60.04963  72.57871  82.30949  89.55069  93.49725  96.37387  97.
97867
##    comp 9   comp 10
##  99.12460 100.00000
```

```
cum.var <- cum_var(pca.eig.3 = pca.scaled$eig[,3], # "pca.scaled$eig[,3]" is
contained within object "pca"
                   thresh = 80) # Custom threshold value (in units percentage
)

cum.var
```

```
## [1] 4
```

Create PCA amplitude plots for each important component. Use amplitude plots to identify relevant plasticity features.

```
amplitude_plots(cum.var = cum.var,# Output of 'cum_var()' function
                pca.var.coord = pca.scaled$var$coord) # "pca.scaled$var$coord
" is contained within object 'pca'
```

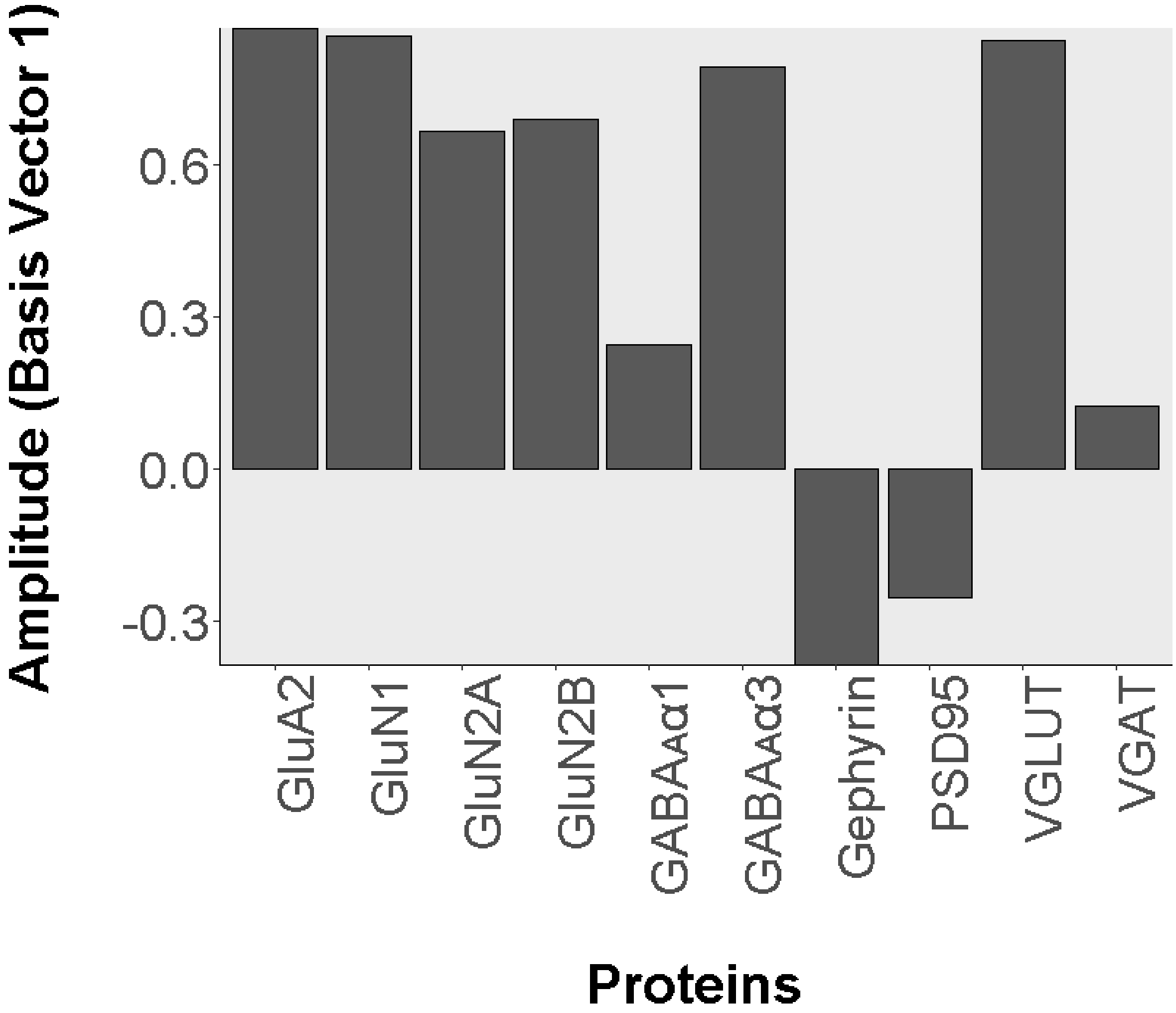
Amplitude (Basis Vector 1)
0.6
0.3
0.0
-0.3
GluA2
GluN1
GluN2A
GluN2B
GABAAα1
GABAAα3
Gephyrin
PSD95
VGLUT
VGAT
Proteins

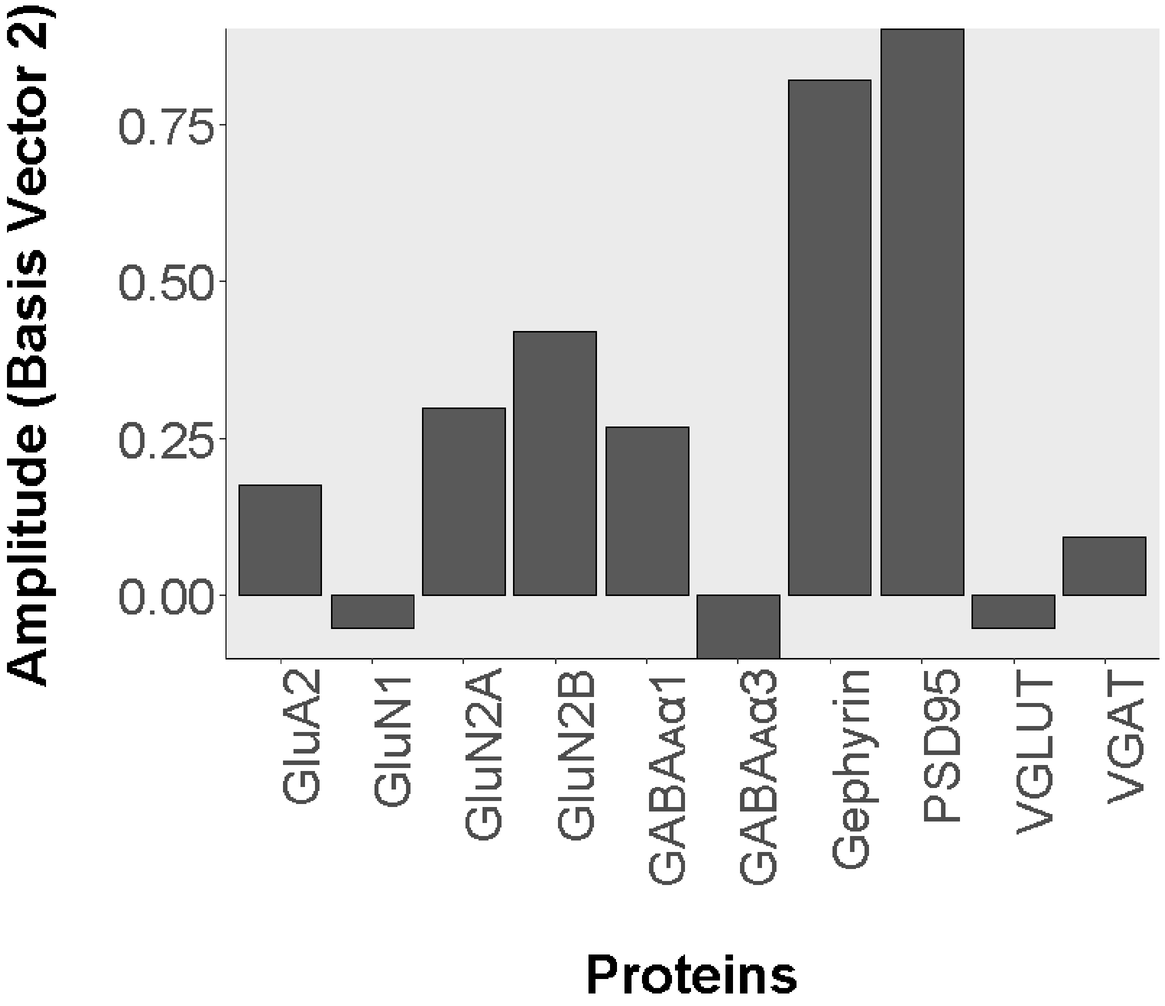

Amplitude (Basis Vector 2)
0.75
0.50
0.25
0.00
GluA2
GluN1
GluN2A
GluN2B
GABAAα1
GABAAα3
Gephyrin
PSD95
VGLUT
VGAT
Proteins

237

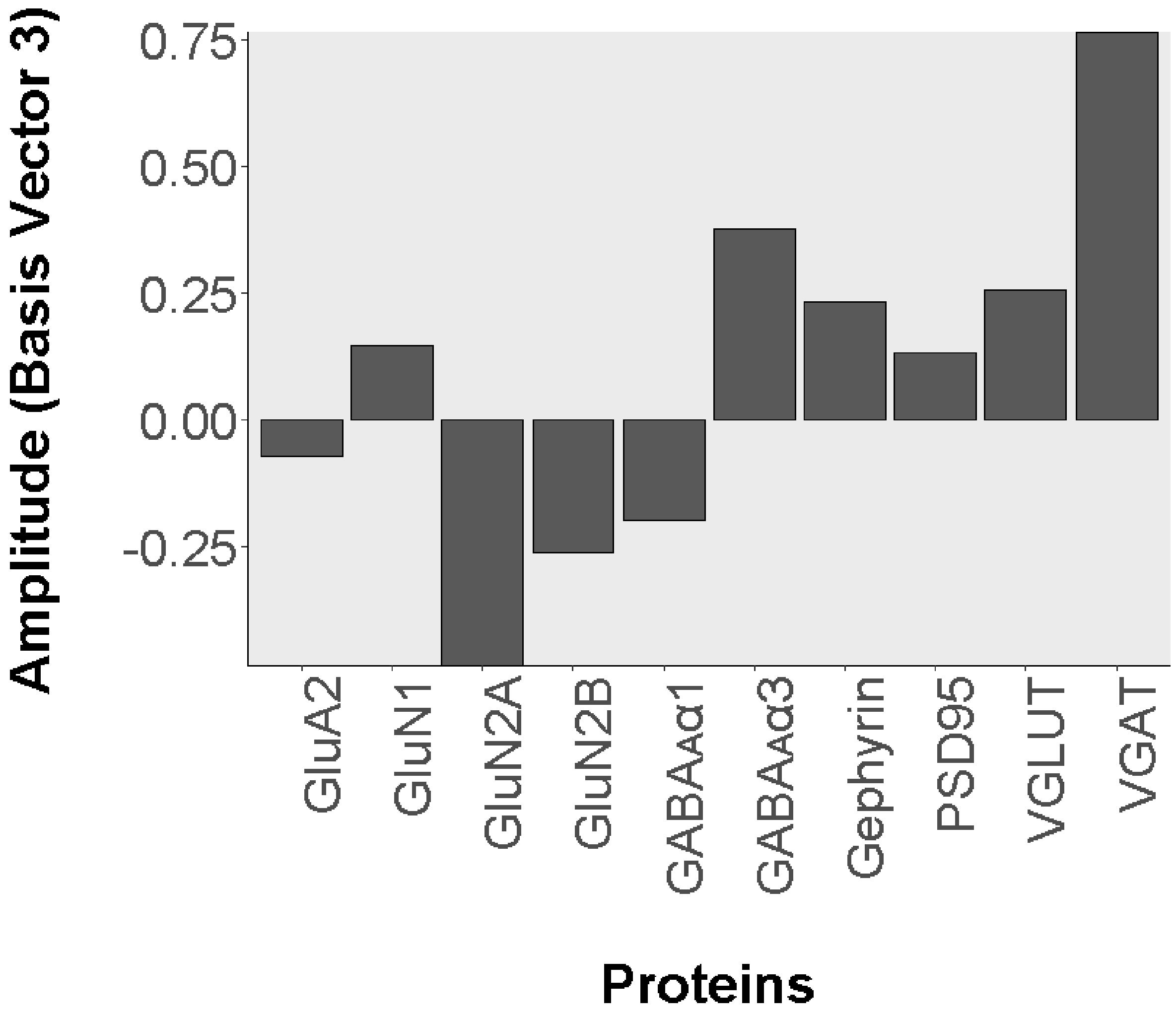
Amplitude (Basis Vector 3)
0.75
0.50
0.25
0.00
-0.25
GluA2
GluN1
GluN2A
GluN2B
GABAAα1
GABAAα3
Gephyrin
PSD95
VGLUT
VGAT
Proteins

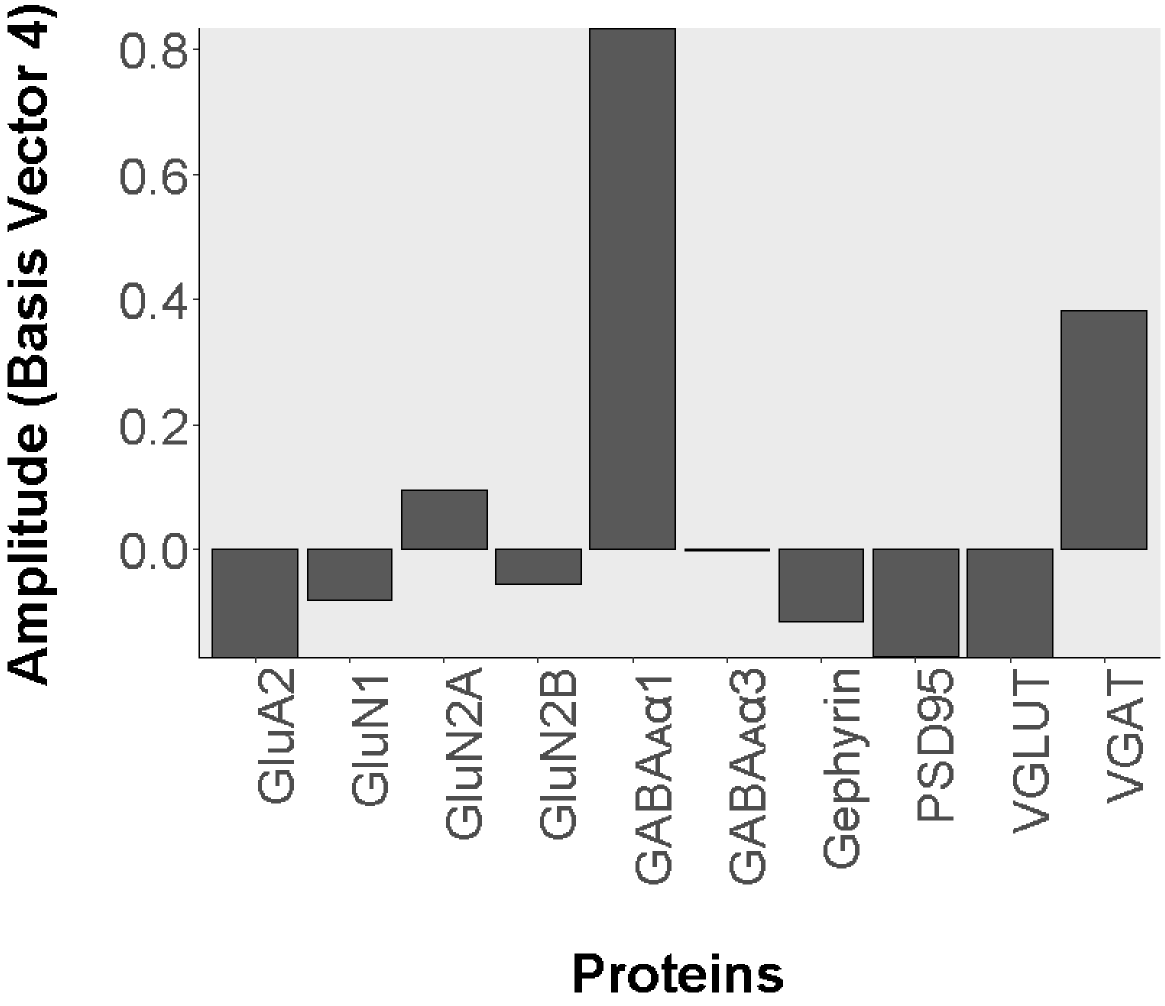

Rat: Application of the Heuristics to Identify Candidate Plasticity Features for Rat V1

Create dataframe, “NewFeatures”, to store plasticity features.

```
NewFeatures <- data.frame(matrix(nrow = nrow(my.data),
                                 ncol = 0))
```

Calculate plasticity features.

```
NewFeatures$'Receptors Sum' <- rowSums(my.data[,1:6])

      ave.rec <- NewFeatures$'Receptors Sum'/ ncol(my.data[,1:6])

NewFeatures$'VGLUT' <- my.data$VGLUT

NewFeatures$'Scaffolding Sum' <- rowSums(my.data[,7:8])
```

```
      ave.scaf <- NewFeatures$'Scaffolding Sum'/ncol(my.data[,7:8])

NewFeatures$'Receptors:Scaffolding' <- (ave.rec - ave.scaf)/
                                       (ave.rec + ave.scaf)

NewFeatures$'VGAT' <- my.data$VGAT

NewFeatures$"GABA\u1D00\u03b13:GABA\u1D00\u03b11" <-
            (my.data$'GABA\u1D00\u03b11' - my.data$'GABA\u1D00\u03b13')/
            (my.data$'GABA\u1D00\u03b11' + my.data$'GABA\u1D00\u03b13')

NewFeatures$'GluN2A:GluN2B' <- (my.data$GluN2A - my.data$GluN2B)/
                               (my.data$GluN2A + my.data$GluN2B)

NewFeatures$'GABA\u1D00\u03b11' <- my.data$'GABA\u1D00\u03b11'

NewFeatures$'VGLUT:VGAT' <- (my.data$VGLUT - my.data$VGAT)/
                            (my.data$VGLUT + my.data$VGAT)
```

Specify order of “NewFeature” columns in the object “NewFeatsCol”.

```
NewFeatsCol <- c("VGAT","VGLUT",'Receptors Sum',
                         'Scaffolding Sum',"GABA\u1D00\u03b11",
                         "GABA\u1D00\u03b13:GABA\u1D00\u03b11" ,
                         "GluN2A:GluN2B",
                         'Receptors:Scaffolding',
                         "VGLUT:VGAT")
```

Rat: Validating Candidate Plasticity Features

Reassign “pca.scaledindcord” object to “PCA.scores”.

```
PCA.scores <- pca.scaled$ind$coord
```

Perform a Bonferroni-corrected, pairwise Pearson’s correlation against the PCA scores and plasticity features. Store the results in the object, “corr.scores.bf”.

```
corr.scores.bf <- corr.test(PCA.scores[,1:cum.var],
                            NewFeatures[,NewFeatsCol],
                            use="pairwise",
                            method="pearson",
                            adjust="bonferroni")

## Warning in abbreviate(colnames(r), minlength = minlength): abbreviate used
with
```

```
## non-ASCII chars

## Warning in abbreviate(colnames(r), minlength = minlength): abbreviate used
with
## non-ASCII chars

## Warning in abbreviate(colnames(r), minlength = minlength): abbreviate used
with
## non-ASCII chars
```

Store correlation coefficients in the object, “corr.scores.rval”.

```
corr.scores.rval <- corr.scores.bf$r

corr.scores.rval
```

```
##              VGAT       VGLUT Receptors Sum Scaffolding Sum   GABA<U+1D00>a
1
## Dim.1 0.12556031  0.85034747     0.8912394      -0.3283217      0.2473832
## Dim.2 0.09308728 -0.05314437     0.2785196       0.9026116      0.2700868
## Dim.3 0.77019116  0.25750384    -0.1797255       0.1862151     -0.2000903
## Dim.4 0.38393459 -0.17496353     0.2982607      -0.1506626      0.8385212
##          GABA<U+1D00>a3:GABA<U+1D00>a1 GluN2A:GluN2B Receptors:Scaffoldin
g
## Dim.1                      -0.2040768    0.11725395            0.6212029
## Dim.2                       0.3033732   -0.03396026           -0.6809633
## Dim.3                      -0.4347533   -0.37020779           -0.2181736
## Dim.4                       0.6250398    0.17726754            0.1624328
##         VGLUT:VGAT
## Dim.1  0.47533836
## Dim.2 -0.08083291
## Dim.3 -0.50327931
## Dim.4 -0.35495764
```

Store adjusted p-values of correlation coefficients in the object, “corr.scores.bfpval”.

```
corr.scores.bfpval <- corr.scores.bf$p

corr.scores.bfpval
```

```
##                VGAT        VGLUT Receptors Sum Scaffolding Sum   GABA<U+1D0
0>a1
## Dim.1 1.000000e+00 4.733524e-21  6.508887e-26    1.279815e-01   1.000000e+
00
## Dim.2 1.000000e+00 1.000000e+00  5.103404e-01    1.279791e-27   6.307897e-
01
## Dim.3 9.569439e-15 8.555534e-01  1.000000e+00    1.000000e+00   1.000000e+
00
```

```
## Dim.4 2.040493e-02 1.000000e+00  3.031827e-01    1.000000e+00   6.535877e-
20
##             GABA<U+1D00>a3:GABA<U+1D00>a1 GluN2A:GluN2B Receptors:Scaffoldin
g
## Dim.1                         1.000000e+00     1.0000000          5.978854e-08
## Dim.2                         2.634037e-01     1.0000000          3.406416e-10
## Dim.3                         2.802401e-03     0.0331084          1.000000e+00
## Dim.4                         4.434046e-08     1.0000000          1.000000e+00
##          VGLUT:VGAT
## Dim.1 0.0004503966
## Dim.2 1.0000000000
## Dim.3 0.0001110059
## Dim.4 0.0553288372
```

Construct plasticity feature matrix.

```
feature_matrix(
  corr.scores.pval = corr.scores.bfpval, # Matrix of adjusted p-values
  corr.scores.rval = corr.scores.rval, # Matrix of correlation coefficients
  thresh = 0.05) # Significance threshold (acceptable values range from 0 - 1
)
```

```
## Warning: Removed 23 rows containing missing values (geom_text).
```

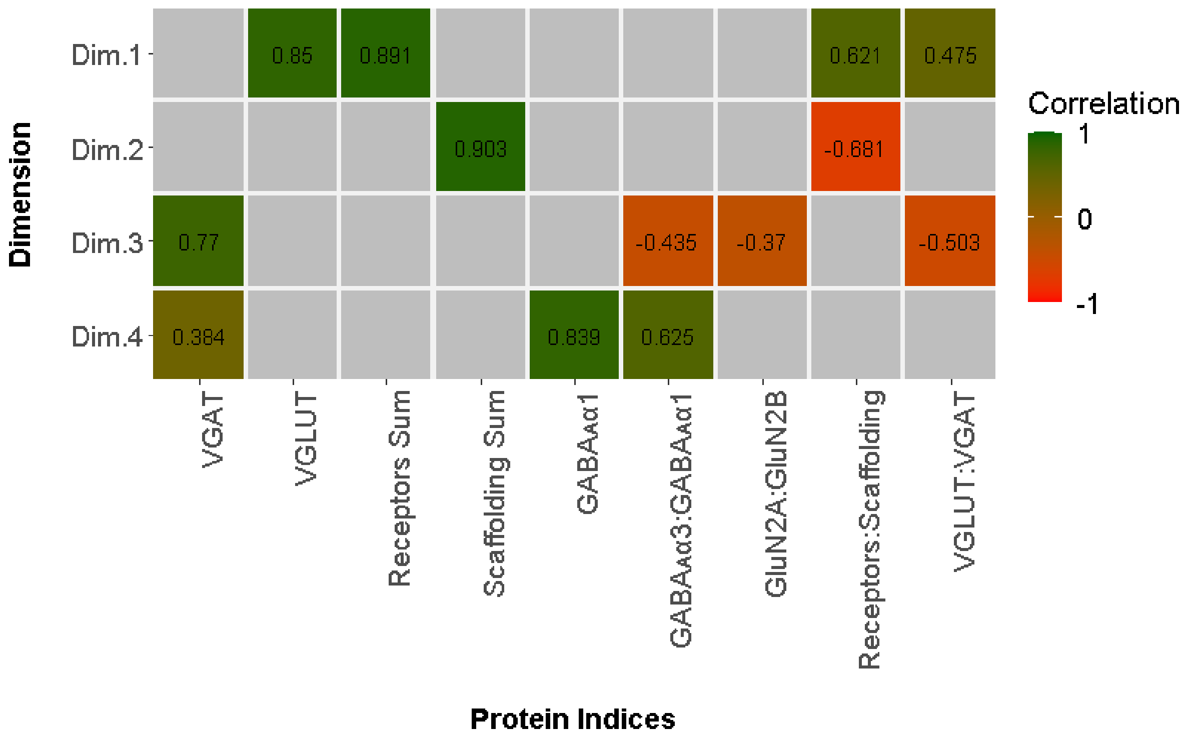

Rat: Using Plasticity Features to Construct a Plasticity Phenotype – Data Processing

Reorder the protein columns of "NewFeatures" according to the order of column names stored in "NewFeatsCol". Assign this reordered data frame to itself.

```
NewFeatures <-  NewFeatures[,NewFeatsCol]
```

Assign row names of "my.data" to row names of "NewFeatures". This is done to allow for the future creation of the bootstrap phenotype.

```
rownames(NewFeatures) <- rownames(my.data)
```

Reassign the "NewFeatures" row names as their own "ids" column. This is done to allow for the subsequent merging of "NewFeatures" with "syn.prots".

```
NewFeatures$ids <- rownames(NewFeatures)
```

Store "my.data" row names as their own column in "syn.prots". This is done to allow for the subsequent merging of "NewFeatures" with "syn.prots".

```
syn.prots$ids <- row.names(my.data)
```

Merge "syn.prots' and 'NewFeatures" data frame by their shared "ids" column, and store the output in the object, "merged.data".

```
merged.data <- merge(NewFeatures,
                syn.prots[,c("Case_Code",
                             "Condition",
                             "Region",
                             "Hemisphere",
                             "Run",
                             "ids")],
                by.x = "ids",
                by.y = "ids")
```

Calculate the median of each plasticity feature in “merged.features[,NewFeatsCol]” across all levels of “list(merged.data$Condition)”. Store the output in the object, “meds”.

```
meds <- aggregate(merged.data[,NewFeatsCol],
                  list(merged.data$Condition),
                  median)
```

Reorder the rows of the "meds" data frame according to the specified order of it "Group.1" column. Reassign the output into the object, "meds".

```
meds <- meds[match(c("normal",
                     "1wk MD",
                     "flx + 1wk MD",
                     "flx"),
             meds$Group.1),]
```

Assign contents of “Group.1” column to row names of object, “meds”.

```
rownames(meds) <- meds$Group.1
```

3.) Rat Phenotype

Rat: Using Plasticity Features to Construct a Plasticity Phenotype – Creating Phenotypes

Create a plasticity phenotype. Function assumes that the first few columns will be sums grouped together, followed by all indices grouped together.

```
plasticity_phenotype(
  df_list = list(meds[,-1]), # Median values data frame
  first_index_column =  6, # Index number of first index column in "meds" (in
dexes begin at 0)
  group_label = "\nRearing Conditions", # X-axis label
  translation = 'absolute') # Desired colour-scale

##                             normal  1wk MD flx + 1wk MD     flx
## VGAT                       #000000 #292929      #464646 #313131
## VGLUT                      #4D4D4D #000000      #7D7D7D #9F9F9F
## Receptors Sum              #383838 #000000      #595959 #545454
## Scaffolding Sum            #595959 #C1C1C1      #000000 #4E4E4E
## GABA<U+1D00>a1               #191919 #3C3C3C      #2C2C2C #000000
## GABA<U+1D00>a3:GABA<U+1D00>a1 #FFDC00 #FFC000      #FFF700 #EFFF00
## GluN2A:GluN2B              #FFC300 #E3FF00      #FFFF00 #FAFF00
## Receptors:Scaffolding      #DCFF00 #00FF00      #FFF800 #FFFF00
## VGLUT:VGAT                 #FCFF00 #B0FF00      #FFF500 #FFD700

## [[1]]
```

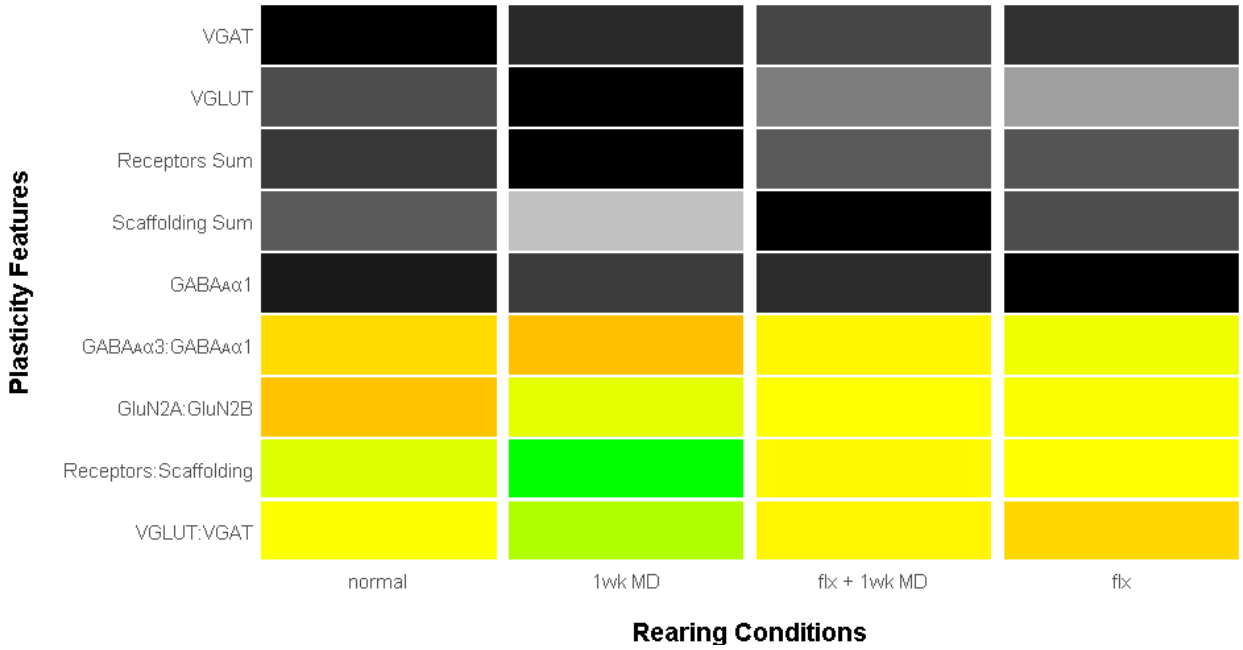

Assign the rat phenotype's colour-code to the object, "rat.cols".

```
rat.cols <- phen.cols[[1]]
```

4.) Create Rat Boxplots

Rat: Using Plasticity Features to Construct a Plasticity Phenotype – Creating Boxplots

Append contents of “NewFeatsCol” with an “\n” and store in “NewFeatsCol2”. This was done to avoid overwriting “NewFeatsCol”.

```
NewFeatsCol2 <- paste0(NewFeatsCol,"\n")
```

Store “merged.data” into “merged.data2”. This was done to avoid overwriting “merged.data”.

```
merged.data2 <- merged.data
```

Assign contents of “NewFeatsCol2” as the plasticity feature column headers of “merged.data2”.

```
colnames(merged.data2)[2:10] <- NewFeatsCol2
```

Order the levels of "merged.data2$Condition" and reassign the output into 'merged.data2$Condition'.

```
merged.data2$Condition <- factor(merged.data2$Condition,
                                 c("normal",
                                   "1wk MD",
                                   "flx + 1wk MD",
                                   "flx"),
                                   ordered = T)
```

Create multiple individual boxplots based on the rat fluoxetine plasticity phenotype.

```
head(merged.data2[,c("Condition",NewFeatsCol2)])
```

```
##   Condition    VGAT\n   VGLUT\n Receptors Sum\n Scaffolding Sum\n
## 1       flx 0.6877782 0.6543510        4.868469          1.611637
## 2       flx 1.0696206 0.8939942        6.091951          1.880370
## 3       flx 1.7799586 0.7829019        4.992009          1.868406
## 4       flx 0.9694615 0.8957831        6.055437          4.962173
## 5       flx 1.0398267 0.9726280        5.514919          2.049094
## 6       flx 2.7974660 0.9304105        7.646494          2.363624
##   GABA<U+1D00>a1\n GABA<U+1D00>a3:GABA<U+1D00>a1\n GluN2A:GluN2B\n
## 1        0.6081925                     -0.27659380      0.18481041
```

```
## 2        1.2728010                            0.04431119      0.13280069
## 3        0.6445332                           -0.28039590     -0.07655649
## 4        1.0276800                           -0.01807704      0.07282728
## 5        0.9234916                           -0.02424368     -0.03311775
## 6        2.3551698                            0.30931242     -0.03443097
##   Receptors:Scaffolding\n VGLUT:VGAT\n
## 1             0.003458318  -0.02490615
## 2             0.038424886  -0.08944036
## 3            -0.057864918  -0.38904056
## 4            -0.421693303  -0.03950063
## 5            -0.054223255  -0.03339143
## 6             0.037701603  -0.50083619
```

```
phenotype_boxplots(feature_df = merged.data2[,c("Condition",NewFeatsCol2)], #
Boxplot data frame
                   phenotype_cols = rat.cols, # Phenotype colour-code data fr
ame
                   max_sum = c(1.5,
                               max(merged.data2$'VGLUT\n'),
                               max(merged.data2$'Receptors Sum\n'),
                               max(merged.data2$'Scaffolding Sum\n'),
                               2),  # Maximum Y-axis values for feature sums'
boxplots
                   group_label = " ", # X-axis label
                   first_index_column = 6, # Index number of first index colu
mn in "merged.data2[,c("Condition",NewFeatsCol2)]" (indexes begin at 0)
                   point_size = 1.5, # Desired size of geom_jitter points
                   point_alpha = 1, # Desired transparency of geom_jitter poi
nts
                   aspect_ratio = 5/7, # Desired aspect ratio of each boxplot
                   text_size = 8) # Desired X- & Y-axis text size for each bo
xplot
```

Create a 3 x 3 matrix displaying all colour-coded boxplots on a single figure.

```
ggarrange(plotlist = plot_list,
          nrow = 3,
          ncol = 3,
          labels = LETTERS[1:9],
          font.label = list(size = 10),
          vjust = 1)
```

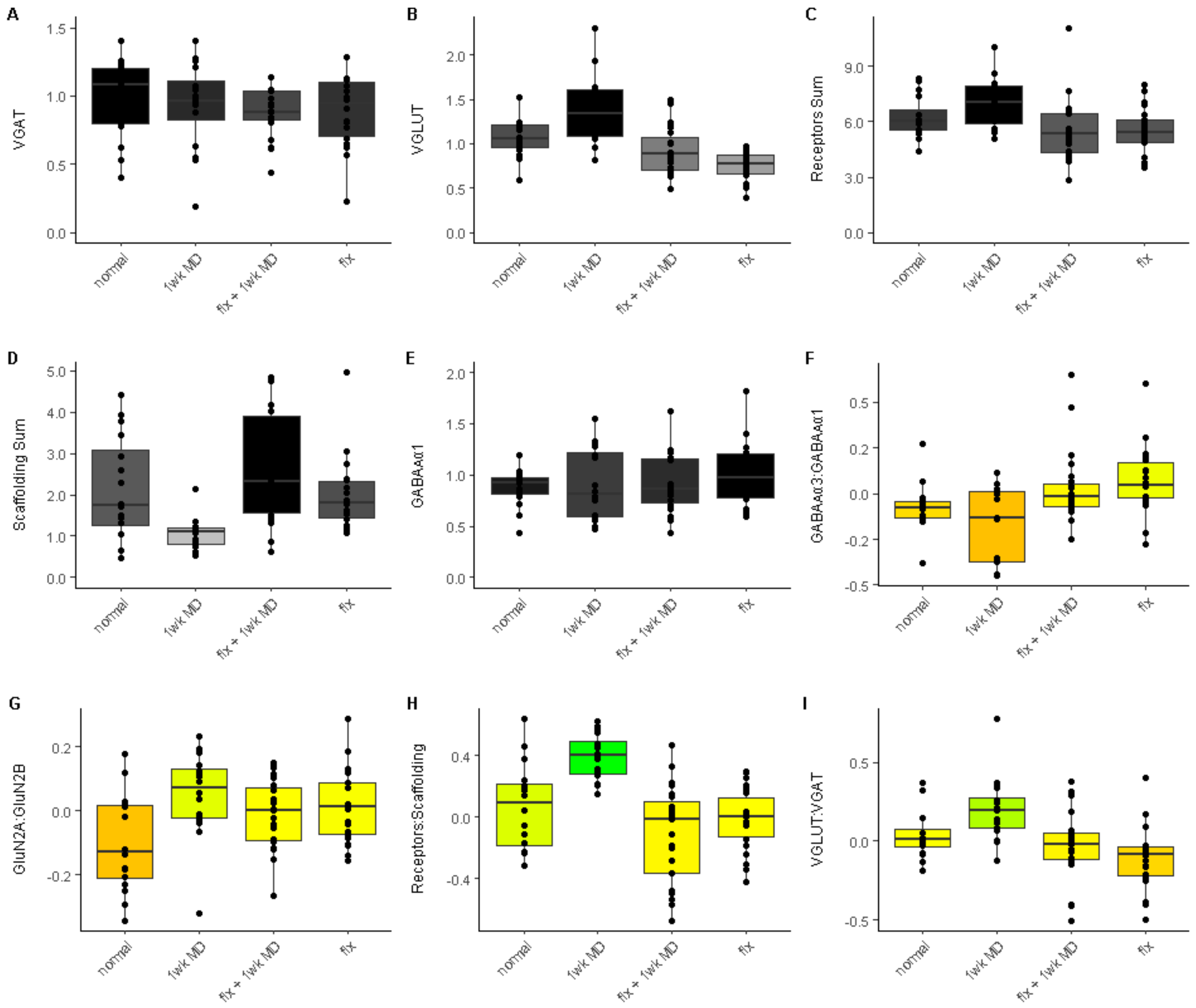
A
VGAT
B
VGLUT
C
Receptors Sum
D
Scaffolding Sum
E
GABAAα1
F
GABAAα3:GABAAα1
G
GluN2A:GluN2B
H
Receptors:Scaffolding
I
VGLUT:VGAT
normal
1wk MD
fix + 1wk MD
fix

5.) Rat Plasticity Phenotype Bootstrap Analysis

Create a phenotype displaying which features in which experimental groups are significantly higher than, lower than, or statistically non-significant in comparison to the “reference_group”.

```
head(NewFeatures[,NewFeatsCol])
```

```
##                            VGAT     VGLUT Receptors Sum Scaffolding Sum
## 231  flx  VC  Cont  1 0.6877782 0.6543510      4.868469        1.611637
## 232  flx  VC  Cont  1 0.9694615 0.8957831      6.055437        4.962173
## 233  flx  VC  Cont  1 0.9891639 0.8531914      8.032260        1.845288
## 234  flx  VC  Cont  1 0.8166134 0.6892650      5.635888        3.067179
## 235  flx  VC  Cont  1 0.6479182 0.3932702      3.808882        2.382254
## 236  flx  VC  Cont  1 1.2881240 0.9291042      7.058869        1.538176
##                       GABA<U+1D00>a1 GABA<U+1D00>a3:GABA<U+1D00>a1
## 231  flx  VC  Cont  1      0.6081925                   -0.27659380
## 232  flx  VC  Cont  1      1.0276800                   -0.01807704
## 233  flx  VC  Cont  1      1.8131427                    0.21992021
## 234  flx  VC  Cont  1      1.1133509                    0.12368031
## 235  flx  VC  Cont  1      0.8327478                    0.18130631
## 236  flx  VC  Cont  1      1.2145710                    0.08319483
##                       GluN2A:GluN2B Receptors:Scaffolding  VGLUT:VGAT
## 231  flx  VC  Cont  1    0.18481041           0.003458318 -0.02490615
## 232  flx  VC  Cont  1    0.07282728          -0.421693303 -0.03950063
## 233  flx  VC  Cont  1    0.28771834           0.183989663 -0.07380365
## 234  flx  VC  Cont  1    0.12088717          -0.240314546 -0.08456751
## 235  flx  VC  Cont  1   -0.04336542          -0.304672207 -0.24457434
## 236  flx  VC  Cont  1   -0.13921701           0.209394110 -0.16192279
```

```
bootstrap_phenotype(features_df_row = NewFeatures[,NewFeatsCol], # Data frame
for bootstrap analysis
                    condition_list = c('  normal  ',
                                       '  1wk MD  ',
                                       '  flx + 1wk MD  ',
                                       '  flx  '), # List of experimental con
ditions as they appear in row names of "features_df_row"
                    reference_group = '  normal  ', # Name of reference group
as it appears in the row names of "features_df_row"
                    group_label = "\nRearing Conditions") # X-axis label
```

535

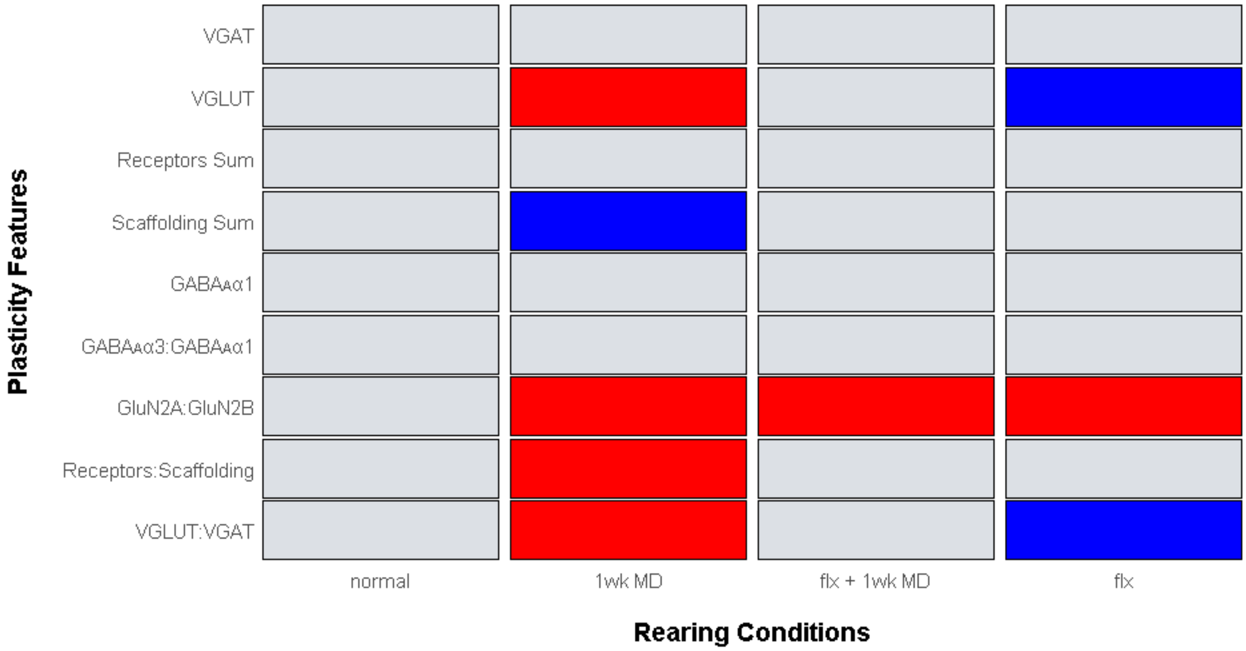
Plasticity Features
VGAT
VGLUT
Receptors Sum
Scaffolding Sum
GABAAα1
GABAAα3:GABAAα1
GluN2A:GluN2B
Receptors:Scaffolding
VGLUT:VGAT
normal
1wk MD
flx + 1wk MD
flx
Rearing Conditions

Rat_CorrHeatmap_Figure14

Table of Contents		
**Section Number**	**Title**	**Page Number**
1	Load Rat Correlation Data	99
2	Process the Rat Correlation Data	103
3	Studying Fluoxetine-enhanced Plasticity in Adult Rat V1 Using a Plasticity Phenotype – Create Heatmap	106

1.) Load Rat Correlation Data

Install the package, “PlasticityPhenotypes”.

```
devtools::install_github("visualneurosciencelab/PlasticityPhenotypes")
```

Load the package, “PlasticityPhenotypes”.

```
library(PlasticityPhenotypes)

## Loading required package: tidyverse

## -- Attaching packages --------------------------------------- tidyverse 1
.3.0 --

## v ggplot2 3.3.0     v purrr   0.3.3
## v tibble  2.1.3     v dplyr   0.8.5
## v tidyr   1.0.2     v stringr 1.4.0
## v readr   1.3.1     v forcats 0.5.0

## -- Conflicts ------------------------------------------ tidyverse_conflic
ts() --
## x dplyr::filter() masks stats::filter()
## x dplyr::lag()    masks stats::lag()

## Loading required package: FactoMineR

## Loading required package: factoextra

## Welcome! Want to learn more? See two factoextra-related books at https://g
oo.gl/ve3WBa

## Loading required package: data.table

##
## Attaching package: 'data.table'

## The following objects are masked from 'package:dplyr':
##
##     between, first, last

## The following object is masked from 'package:purrr':
##
##     transpose

## Loading required package: ggpubr

## Loading required package: magrittr

##
## Attaching package: 'magrittr'
```

```
## The following object is masked from 'package:purrr':
##
##     set_names

## The following object is masked from 'package:tidyr':
##
##     extract

## Loading required package: corrplot

## corrplot 0.84 loaded

## Loading required package: psych

##
## Attaching package: 'psych'

## The following objects are masked from 'package:ggplot2':
##
##     %+%, alpha

## Loading required package: httr

library(gplots)

##
## Attaching package: 'gplots'

## The following object is masked from 'package:stats':
##
##     lowess

library(RColorBrewer)
library(Hmisc)

## Loading required package: lattice

## Loading required package: survival

## Loading required package: Formula

##
## Attaching package: 'Hmisc'

## The following object is masked from 'package:psych':
##
##     describe

## The following objects are masked from 'package:dplyr':
##
##     src, summarize
```

```
## The following objects are masked from 'package:base':
##
##     format.pval, units
```

Store the file path in a unique object.

```
raw.rat.corr <- "https://osf.io/z2asv//?action=download"
```

Import the necessary CSV from OSF. The “raw.data” object consists of 10 columns - 1 attributes column (“ids”) and 7 plasticity feature columns.

```
filename <- 'rat_correlation.csv'
GET(raw.rat.corr, write_disk(filename, overwrite = TRUE))
```

```
## Response [https://files.ca-1.osf.io/v1/resources/8a3kx/providers/osfstorag
e/5ecd3179c7568601d42d2039?action=download&direct&version=1]
##   Date: 2020-06-02 20:17
##   Status: 200
##   Content-Type: text/csv
##   Size: 13.2 kB
## <ON DISK>  C:\Users\dezia\Dropbox (Kathy Murphy)\JB-ProteinAnalysisWorkflo
w\JB ProteinAnalysisWorkflow Markdowns\RMD files\Rat Analysis\rat_correlation
.csv
```

```
raw.data <- read.csv(filename)
head(raw.data)
```

```
##                             ids      VGAT     VGLUT Receptors.Sum
## 1 231  fluoxetine  VC  Cont  1 0.6877782 0.6543510      4.868469
## 2 232  fluoxetine  VC  Cont  1 0.9694615 0.8957831      6.055437
## 3 233  fluoxetine  VC  Cont  1 0.9891639 0.8531914      8.032260
## 4 234  fluoxetine  VC  Cont  1 0.8166134 0.6892650      5.635888
## 5 235  fluoxetine  VC  Cont  1 0.6479182 0.3932702      3.808882
## 6 236  fluoxetine  VC  Cont  1 1.2881240 0.9291042      7.058869
##   Scaffolding.Sum GABA.U.1D00.a1 GABA.U.1D00.a1.GABA.U.1D00.a3 GluN2A.GluN
2B
## 1        1.611637      0.6081925                   -0.27659380    0.184810
41
## 2        4.962173      1.0276800                   -0.01807704    0.072827
28
## 3        1.845288      1.8131427                    0.21992021    0.287718
33
## 4        3.067179      1.1133509                    0.12368031    0.120887
17
## 5        2.382254      0.8327478                    0.18130631   -0.043365
42
## 6        1.538176      1.2145710                    0.08319483   -0.139217
01
```

```
##   Receptors.Scaffolding  VGLUT.VGAT                        ids.1
## 1           0.003458318 -0.02490615 231  fluoxetine  VC  Cont  1
## 2          -0.421693303 -0.03950063 232  fluoxetine  VC  Cont  1
## 3           0.183989663 -0.07380365 233  fluoxetine  VC  Cont  1
## 4          -0.240314546 -0.08456751 234  fluoxetine  VC  Cont  1
## 5          -0.304672207 -0.24457434 235  fluoxetine  VC  Cont  1
## 6           0.209394110 -0.16192279 236  fluoxetine  VC  Cont  1
```

2.) Process the Rat Correlation Data

Split the character strings in “raw.data$ids” into 5 different columns, and assign them in the object, “ids”. Rename the “ids" column headers.

```
ids <- as.data.frame(str_split_fixed(raw.data$ids,
                                      " ",
                                      5))

colnames(ids) <- c('id','condition','location','hemisphere','run')

head(ids)
```

```
##    id  condition location hemisphere run
## 1 231 fluoxetine       VC       Cont   1
## 2 232 fluoxetine       VC       Cont   1
## 3 233 fluoxetine       VC       Cont   1
## 4 234 fluoxetine       VC       Cont   1
## 5 235 fluoxetine       VC       Cont   1
## 6 236 fluoxetine       VC       Cont   1
```

Rename the factor levels of “ids$condition” to be consistent with (Beshara et al., 2015) and reassign to itself.

```
ids$condition <- plyr::mapvalues(ids$condition,
                          unique(ids$condition),
                          c("flx",
                            "flx + 1wk MD",
                            "normal",
                            "1wk MD"))
```

Split the “ids” object into 5 columns (“V1 - V5”). Concatenate the “id” and “condition” columns and separate them using a “-”. Assign these newly created character strings in the novel column, “ids$rat_id”, and store full output in the object, “ids2”.

```
ids.2 <- unite(ids,rat_id,
               c(id,
                 condition),
               sep = "-",
               remove = FALSE)
```

Select both the “rat_id” and “runs” column of “ids.2”, and store in the object, “ids.3”.

```
ids.3 <- ids.2[,c('rat_id','run')]
```

Bind plasticity feature columns of “raw.data” and “ids.3$run” column, and store in the object, “raw.data.2”.

```
raw.data.2 <- cbind(raw.data[,-ncol(raw.data)],
                     ids.3$run)
```

Rename the last column of “raw.data.2” to “run”.

```
colnames(raw.data.2)[ncol(raw.data.2)] <- "run"
```

Calculate the median of each feature across multiple runs, and store in “med.df”.

```
all.df <- group_by(raw.data.2[,-ncol(raw.data.2)],
                   ids.3$rat_id)


med.df <- summarise_at(all.df,
                       vars(colnames(raw.data.2)[2:10]),
                       median)
```

Rename the first column of “med.df” to “rat_id”. Transform “med.df$rat_id” from a column to individual row names. Store the output into the object, “med.df.2”.

```
colnames(med.df)[1] <- 'rat_id'

med.df.2 <- column_to_rownames(med.df,
                               var = 'rat_id')
```

Transpose the “med.df.2”, and store in “t.med.df”.

```
t.med.df <- as.matrix(t(med.df.2))
```

Create empty data frame to store the “ids”, “treatments”, and “conditions” columns of “med.df.2” and store in “id.labels”.

```
id.labels <- as.data.frame(matrix(nrow = nrow(med.df.2), ncol = 2))
```

Rename columns of “id.labels”.

```
colnames(id.labels) <- c("ids",
                         "groups")
```

Assign rownames of “med.df.2” to ‘ids’ column of ‘ids.labels’.

```
id.labels$ids <- rownames(med.df.2)
```

Assign experimental conditions of each animal within the row names of “med.df.2” to the “groups” column of “id.labels”.

```
id.labels$groups <- as.data.frame(
  str_split_fixed(rownames(med.df.2), "-",4))[,2]
```

3.) Studying Fluoxetine-enhanced Plasticity in Adult Rat V1 Using a Plasticity Phenotype – Create Heatmap

Assign colors to each animal condition of “ids.labels” (flx = “#008000”/green, flx + 1wk MD = “#FF0000”/red, normal = “#000000”/black, 1wk MD = “#D3D3D3”/grey).

```
id.labels$colors <- plyr::mapvalues(id.labels$groups,
                                    unique(id.labels$groups),
                                    c("#008000",
                                      "#FF0000",
                                      "#000000",
                                      "#D3D3D3"))
```

Perform a Pearson’s correlation against all animals to each other, and store in ‘data.correlation’.

```
data.correlation <- rcorr(as.matrix(t.med.df),
                          type ="pearson")
```

Save the correlations coefficients as a matrix in “CorMat”.

```
CorMat <- as.matrix(data.correlation$r)
```

Convert the correlation matrix into a distance matrix (stored in “distance.row”), and perform hierarchical clustering upon this distance matrix. Store this hierarchical clustered object into “cluster.row”.

```
distance.row <- dist(as.matrix(CorMat),
                     method = "euclidean")

cluster.row <- hclust(distance.row,
                      method = "ward.D2")
```

Transform “cluster.row” into a dendrogram which is stored in “dd”.

```
dd <- as.dendrogram(cluster.row)
```

Create a color palette for the heatmap.

```
nb.cols <- 25

mycolors <- colorRampPalette(brewer.pal(8, "YlOrRd"))(nb.cols)
```

Sets the heatmap’s margins.

```
par(oma=c(rep(7,4)))
```

Create the heatmap.

```
heatmap.2(CorMat,
          cexRow=1,
          Colv = dd,
          Rowv = dd,
          cexCol=1,
          breaks = seq(0.9,1, length.out = 26),
          trace="none",
          col = mycolors,
          RowSideColors =
            as.character(id.labels$colors),
          ColSideColors =
            as.character(id.labels$colors),
          symm=T,
          colsep=c(2,11,16),
          rowsep=c(28 - c(2,11,16)),
          sepcolor = 'black')
```

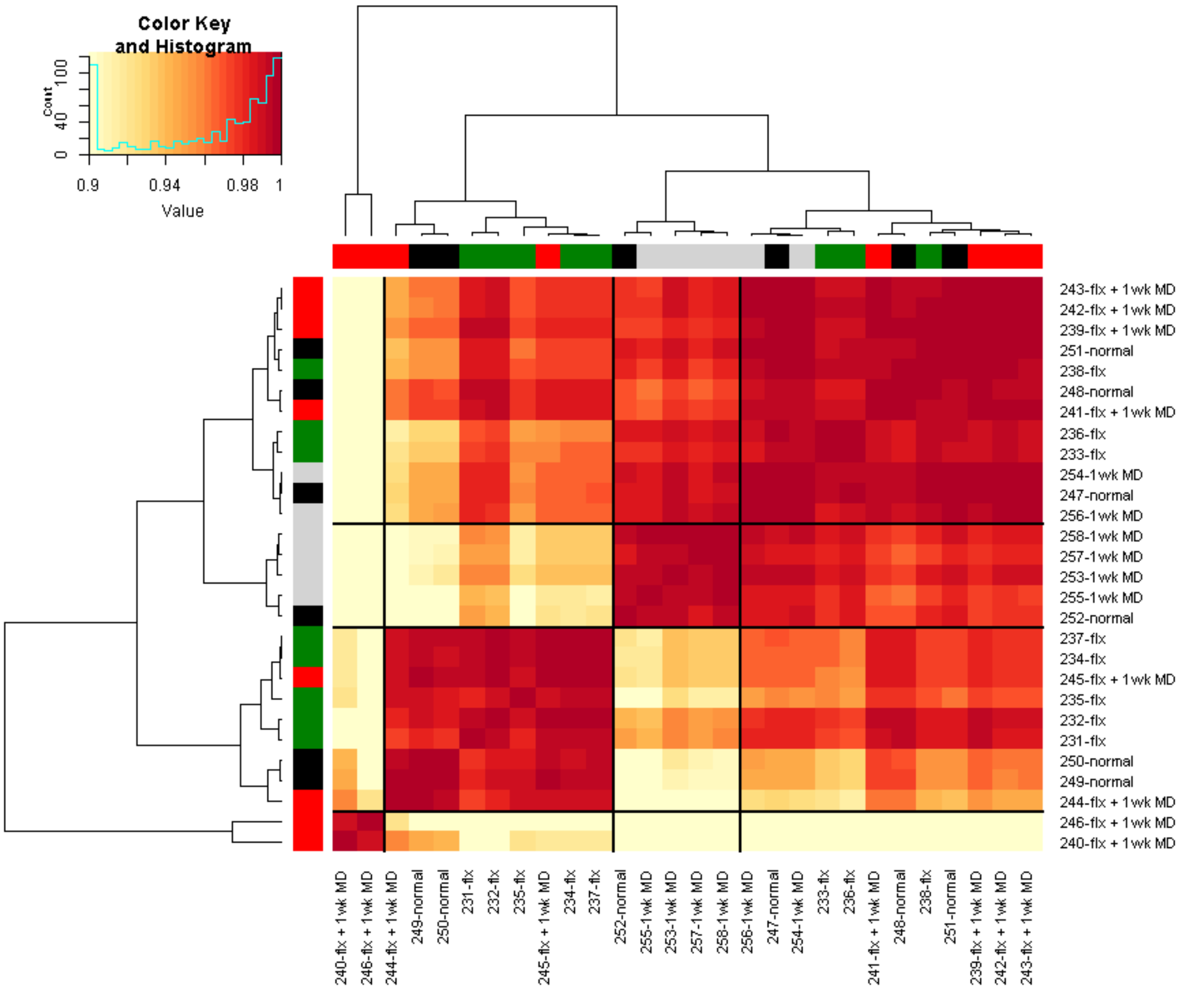
Color Key
and Histogram
Count
0
40
100
0.9
0.94
0.98
1
Value
243-flx + 1wk MD
242-flx + 1wk MD
239-flx + 1wk MD
251-normal
238-flx
248-normal
241-flx + 1wk MD
236-flx
233-flx
254-1wk MD
247-normal
256-1wk MD
258-1wk MD
257-1wk MD
253-1wk MD
255-1wk MD
252-normal
237-flx
234-flx
245-flx + 1wk MD
235-flx
232-flx
231-flx
250-normal
249-normal
244-flx + 1wk MD
246-flx + 1wk MD
240-flx + 1wk MD
240-flx + 1wk MD
246-flx + 1wk MD
244-flx + 1wk MD
249-normal
250-normal
231-flx
232-flx
235-flx
245-flx + 1wk MD
234-flx
237-flx
252-normal
255-1wk MD
253-1wk MD
257-1wk MD
258-1wk MD
256-1wk MD
247-normal
254-1wk MD
233-flx
236-flx
241-flx + 1wk MD
248-normal
238-flx
251-normal
239-flx + 1wk MD
242-flx + 1wk MD
243-flx + 1wk MD
